# Practice recommendations for physical activity promotion in exercise therapy, physical therapy, and other movement-based therapies: a co-design project (PRO-BT) in German medical rehabilitation

**DOI:** 10.1186/s13102-026-01638-4

**Published:** 2026-03-04

**Authors:** Wolfgang Geidl, Gorden Sudeck, Leon Matting, Andrés Jung, Klaus Pfeifer

**Affiliations:** 1 Department of Sport Science and Sport, Friedrich-Alexander University Erlangen-Nürnberg (FAU), Gebbertstraße 123b, Erlangen, 91058 Germany; 2 Education & Health Research, Institute of Sports Science, Faculty of Economics and Social Sciences, Eberhard Karls Universität Tübingen, Tübingen, 72074 Germany; 3 Department of General Practice and Primary Care, Ulm University Hospital, Ulm, 89081 Germany

**Keywords:** Behaviour change, Physical activity-related health competence, Physical literacy, Noncommunicable diseases

## Abstract

**Background:**

Physical activity (PA) benefits individuals with noncommunicable diseases, yet its promotion is inconsistently implemented in medical rehabilitation. In Germany, exercise and physical therapists are central to PA promotion, but a lack of practical, evidence-based guidance has hindered integration into therapy. The *PRO-BT* project addresses this gap by developing consensus-based, evidence-informed practice recommendations for movement-based therapies.

**Methods:**

The project employed a structured four-step approach: (1) evidence update via scoping review, overview of systematic reviews on intervention effectiveness, and national guidelines analysis; co-design phase with (2) iterative draft development through expert workshops and patient focus groups; (3) nationwide stakeholder consultation with rehabilitation professionals to evaluate completeness, clarity, and practical relevance; (4) a final consensus meeting for approval.

**Results:**

The process resulted in 15 evidence-based practice recommendations for PA promotion in movement-based therapies across German medical rehabilitation, organised into 3 sections: overarching competence-oriented guidance; didactic-methodological orientation; and specific therapeutic processes (e.g., assessment, goal setting, delivery, and monitoring). Each recommendation includes background text providing theoretical rationale, examples, and implementation advice. Stakeholder feedback showed strong approval of the recommendations (86%–100% full endorsement) and positive ratings for background texts in terms of completeness (93%), clarity (89%), and practical relevance (78%).

**Conclusion:**

The *PRO-BT* project provides nationally endorsed practice recommendations for PA promotion in movement-based therapies in German rehabilitation. These recommendations provide guidance for movement-based therapists to integrate PA promotion into routine practice, particularly for individuals with noncommunicable diseases. The co-design process underpins broad acceptance and applicability, offering an internationally adaptable methodological model for developing context-specific rehabilitation guidance.

**Supplementary Information:**

The online version contains supplementary material available at 10.1186/s13102-026-01638-4.

## Background

Physical activity (PA) is widely recognised for its substantial health benefits, particularly for individuals with noncommunicable diseases (NCDs), such as cardiovascular, metabolic, and mental health conditions [1, 2]. Despite this, physical inactivity remains highly prevalent among individuals with NCDs [3–5], contributing to poorer health outcomes including decreased quality of life [6], lower life expectancy [7], and increased healthcare costs [8].

The healthcare sector holds considerable potential for promoting PA, with healthcare professionals increasingly being seen as key actors in supporting people with NCDs to adopt active lifestyles. This perspective is reflected in international policy documents, such as the World Health Organization’s (WHO) Global Action Plan on Physical Activity (GAPPA) [9] and the Physical Activity Strategy for the WHO European Region 2016–2025 [10], which call for the systematic integration of PA promotion into healthcare systems.

Several initiatives are aimed at firmly anchoring the promotion of PA in the healthcare system. For instance, there have been calls for PA promotion-related competencies to be core components of all health professional training programmes [11]. National initiatives, such as *Moving Medicine*, provide profession-specific guidance and practical tools to support health professionals, particularly physicians, in delivering effective PA counselling [12]. Key steps for integrating PA promotion into the curricula of healthcare professionals have also been proposed [13, 14].

However, the healthcare sector’s potential to promote PA remains underutilised. While many healthcare professionals increasingly recognise PA promotion as part of their professional responsibility [15], and certain health disciplines, —such as physical therapy—are aligning more closely with this goal [16–18], the practical implementation of PA promotion continues to be inconsistent and often suboptimal [19]. This gap can be partly attributed to barriers on the part of healthcare professionals, including limited knowledge about and lack of skills in PA promotion, low confidence in their ability to implement it effectively, and insufficient familiarity with the most effective evidence-based strategies for promoting PA [15, 20].

The discrepancy between the benefits of PA promotion and its limited practical implementation is also evident in the German medical rehabilitation system, which provides services to approximately 1 million individuals annually [21]. Two core components of these medical rehabilitation services include physical therapy and the so-called *Sport- und Bewegungstherapie* (hereinafter referred to as sport therapy) [22], which we both conceptually subsume, as introduced previously [23], under the broader category of movement-based therapies. Sport therapy encompasses structured, whole-body physical activities to improve both physical and psychological capacities, while promoting sustained engagement in PA. It is predominantly delivered in group settings, facilitating social interaction and peer support. The therapy is implemented in various formats, including ergometer training, circuit training, running exercises, gymnastics, swimming, and age- and condition-adapted games [22]. Its delivery involves multiple professional groups, such as sport therapists, physical therapists, exercise physiologists, and kinesiologists. According to mandates from major healthcare payers, such as the German Pension Insurance (DRV), one of the primary goals of the sport therapy in this context is to strengthen individuals’ competencies for initiating and maintaining a physically active lifestyle [22]. Quality-management policy documents for German medical rehabilitation [22, 24] suggest that competencies described in the Physical Activity-related Health Competence (PAHCO) model [25–27] may serve as a useful reference when shaping PA promotion strategies. The PAHCO model conceptualises the individual prerequisites for a healthy and physically active lifestyle across three interrelated sub-competencies: movement competence (the ability to perform and participate in physical activities), control competence (the ability to regulate exercise intensity and load in a health-enhancing manner), and self-regulation competence (the motivational and volitional capacity to initiate and maintain physical activity over time) [25–27].

However, findings of a national survey conducted within the German medical rehabilitation system (the “BewegtheReha” project) indicate substantial variability in the implementation of PA-promoting practices across rehabilitation centres [28–30]. Although movement-based therapists in Germany generally acknowledge the importance of promoting PA, consistent PA-promoting practices are implemented in only approximately half of therapy departments [31].

Improving the quality of PA promotion in German medical rehabilitation requires structural changes at multiple levels. Organisational development, staff training, and resource allocation have been identified as key areas for improvement [28]. A major barrier to progress is the lack of practical, consensus-based recommendations to guide therapy teams in delivering effective and consistent PA promotion.

The current project, funded by the German Pension Insurance, seeks to address this gap by developing evidence-based, consensus-driven practice recommendations for PA promotion within sport therapy in German medical rehabilitation settings. Specifically, the project aims to define how PA promotion by movement-based therapists can be optimally designed in this context. To achieve this aim, the recommendations were developed building on an existing analysis of the current state of PA promotion in German medical rehabilitation derived from a previous research project [28], combined with a synthesis of existing interventions and theoretical frameworks from exercise therapy, physical therapy, sport therapy, and related movement-based disciplines, and a review of the available evidence on intervention effectiveness. This multi-step, evidence-informed process was completed by structured expert involvement from both research and clinical practice to ensure relevance, feasibility, and acceptance, resulting in recommendations intended to support movement-based therapists in integrating PA promotion into routine rehabilitation practice.

## Methods

The development of these recommendations was guided by the current evidence base and considerations of practical feasibility in real-world settings. Building on an evidence update, we conceptualised the project as a co-creation approach, defined as the active involvement of stakeholders across the research process [32, 33]. Within this overarching co-creation framework, co-design guided the collaborative development of the recommendations. Co-design is characterised by the active collaboration of stakeholders in designing solutions to a prespecified problem, emphasising participants’ needs, expertise, and knowledge as essential resources, and fostering reciprocal relationships that support the development of context-sensitive and practically relevant solutions [32]. Consistent with Vargas et al. [32] and Leask et al. [33], co-design in this project focused on the collaborative development and iterative refinement of solutions to a predefined problem, while agenda setting and evidence synthesis remained the responsibility of the academic research team.

The primary target users of the recommendations are movement-based therapists working in German medical rehabilitation, including physical therapists, sport and exercise therapists, and related professionals involved in delivering movement-based therapies. Secondary target users include multidisciplinary rehabilitation teams, rehabilitation service providers, and organisations involved in the planning, implementation, and evaluation of physical activity promotion.

**Fig. 1:**
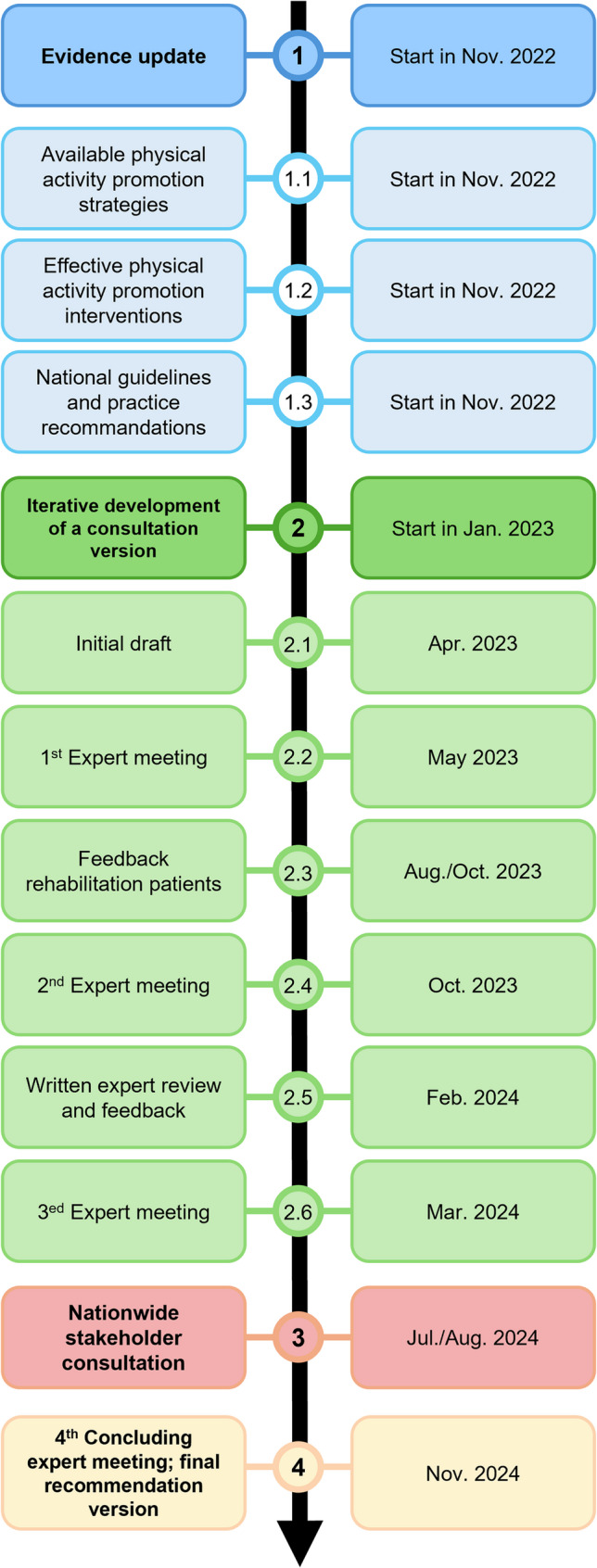
Timeline and sequence of steps involved in development of the practice recommendations for physical activity promotion in movement-based therapies

A structured four-step process was followed (Fig. 1): (1) evidence updates by the academic research team on available PA promotion strategies (1.1), effective interventions (1.2), and related national guidelines and practice recommendations (1.3); (2) co-design of the recommendations and background text through iterative drafting of the recommendations, structured expert workshops, drawing on the expertise of movement-based therapists and other stakeholders as well as focus groups with rehabilitands; (3) a nationwide stakeholder consultation; and (4) a final consensus meeting to formally approve the recommendations.

### Step 1: Evidence update

To establish a robust scientific foundation for these recommendations, a comprehensive evidence update was conducted using 3 complementary approaches.

A scoping review of intervention studies and theoretical works (Step 1.1). This scoping review aimed at offering a broad, detailed exploration of available PA promotion intervention studies and theoretical works within movement-based therapies. The primary research question was as follows: What concepts are available for movement-based therapists (e.g., physical, exercise, and sports therapists) to promote PA in individuals with noncommunicable diseases? A combined deductive-inductive content analysis was conducted, guided by the cybernetic therapy process model proposed by Werle et al. [34], focusing on components such as assessment tools, behaviour change techniques (BCTs) coded according to Michie et al.‘s taxonomy [35], PA practice elements, and didactic-methodological principles.

An overview of systematic reviews (Step 1.2). A synthesis of systematic reviews was performed to evaluate the effectiveness of movement-based interventions aimed at promoting PA in individuals with NCDs. This synthesis was conducted according to recent guidelines [36, 37].

The scoping review (Step 1.1) and the overview of systematic reviews (Step 1.2) were prospectively registered on the Open Science Framework (OSF; registrations DOI 10.17605/OSF.IO/AXZSJ and 10.17605/OSF.IO/JFGHM, including detailed descriptions of research questions, eligibility criteria, search strategies, and analytic methods.

A review of national guidelines and practice recommendations (Step 1.3). As part of the development process, national guidelines and practice recommendations relevant to the rehabilitation setting and PA promotion, including grey literature, were analysed as a separate methodological step. The documents were screened for references to PA and its promotion. Relevant excerpts were extracted and categorized according to the cybernetic therapy process model by Werle et al. [34], assigning content to the domains of assessment, therapy goals, content, implementation, and evaluation. The analysis of national guidelines and practice recommendations (Step 1.3), followed a structured approach but was not preregistered. Findings from the evidence update informed the subsequent development of the recommendations by identifying relevant intervention components and implementation gaps. Not all intervention elements and behaviour change techniques identified were translated into explicit recommendations or tables. Instead, components were synthesised based on their relevance for the three competence facets of the PAHCO model (movement competence, control competence, and self-regulation competence), as well as their feasibility and applicability within movement-based therapy in medical rehabilitation. Identified implementation gaps and heterogeneity in current practice (regarding interdisciplinarity, patient-centeredness, the application of theory- and evidence-based biopsychosocial therapy concepts, and the degree of manualisation and standardisation) [28] were used to prioritise recommendation topics and to inform how intervention components should address and combine these competence facets. These evidence-informed inputs formed the basis for the subsequent co-design and consultation processes.

### Step 2: Iterative co-design of a consultation version

Steps 2–4 followed participatory research principles [32, 33] and involved rehabilitation professionals, rehabilitation patients, representatives from professional societies, and representatives of the service provider (DRV). Particular emphasis was placed on integrating the perspectives of sport therapists and physical therapists, as well as other members of the rehabilitation team (e.g., physicians, psychologists), and rehabilitation patients.

Participants for Step 2 were selected using a purposeful sampling approach. Inclusion criteria for professionals were (a) practical and/or scientific expertise in medical rehabilitation and (b) experience or demonstrated expertise in PA promotion. No formal exclusion criteria were defined. For rehabilitation patients, inclusion focused on experience with movement-based therapies in medical rehabilitation and the ability to provide feedback on clarity, relevance, and usability of the recommendations.

Target sample sizes for the different participatory formats (expert workshops, written feedback, and focus groups) were defined pragmatically based on feasibility and methodological considerations typical for co-design processes, with the aim of achieving sufficient diversity of perspectives rather than statistical representativeness.

First, the research team (2 professors, 1 postdoctoral researcher, and 2 doctoral students), developed an initial draft (Step 2.1). For this development, we drew on the evidence update and findings from the German *BewegtheReha* project [28, 30, 31, 38], which identified substantial heterogeneity and implementation gaps in current PA-promoting practices within movement-based therapies in the context of medical rehabilitation.

The prioritisation of perspectives during this phase was guided by the intended target users of the recommendations. As the recommendations are primarily designed to support movement-based therapists in delivering PA promotion within routine rehabilitation practice, particular emphasis was placed on the perspectives of sport and physical therapists and other rehabilitation professionals.

To develop a consultation version for the nationwide stakeholder consultation, the initial draft was revised through iterative steps in a participatory process, allowing for continuous refinement based on 2 expert workshops (Steps 2.2 and 2.4) as well as written review and feedback (Step 2.5) from clinicians, therapists, and researchers to evaluate content completeness, practicality and feasibility, clarity and comprehensibility of the practical implications. Focus groups with rehabilitation patients (2.3) were conducted selectively to assess the clarity, relevance, and usability of the draft recommendations from a service user perspective. Subsequently, a final meeting with the expert group from the workshops was held to achieve consensus on the recommendations and the content of the accompanying background documents (2.6).

Health benefits of PA promotion were informed by the available evidence on effectiveness, while potential risks and side effects were considered implicitly through the rehabilitation setting, existing clinical standards, and delivery by qualified health professionals. No separate formal risk–benefit assessment was conducted for individual recommendations.

Overall, the formulation and revision of the recommendations followed an iterative co-design process informed by the evidence update, expert judgement, and stakeholder feedback. No predefined decision rules or quantitative thresholds were applied; instead, revisions were guided by structured discussion, consensus-oriented deliberation, and considerations of clarity, feasibility, and relevance for the intended end users.

### Step 3: Nationwide stakeholder consultation

The co-designed consultation version was disseminated as part of a nationwide online consultation. The consultation was conducted using an online survey tool (LimeSurvey) and was distributed via an information letter through established dissemination channels, including the German Pension Insurance (DRV) newsletter.

All leading movement-based therapists and chief physicians of adult medical rehabilitation departments in Germany (approximately 1,200 clinics) were invited to participate, corresponding to a full population approach rather than a targeted sample. Practitioners were asked to indicate their level of agreement with each recommendation using a 4-point Likert scale and to review the accompanying background texts regarding completeness, clarity, and practical usefulness. In addition, open-text fields allowed participants to provide written comments and suggestions for revision.

The nationwide consultation was conducted between 10 July and 31 August 2024, with one reminder sent on 5 August 2024. The consultation aimed to ensure broad stakeholder engagement and validation of the recommendations within routine clinical practice contexts.

### Step 4: Concluding meeting to approve the recommendations

Following the nationwide consultation, the research team systematically reviewed and synthesised the quantitative ratings and qualitative feedback. A concluding consensus workshop was then held with experts involved in the preceding co-design and consultation phases to review the results of the national consultation, discuss proposed revisions, and formally approve the final version of the recommendations.

All experts who had participated in the earlier consensus workshops were invited to this final meeting. During the workshop, participants were informed about the changes made based on the consultation feedback and were given the opportunity to discuss and rate their agreement with the revised recommendations using an online voting tool. The workshop resulted in the final approval of the practice recommendations.

No fixed update cycle was predefined for these recommendations; future updates are intended to be guided by emerging scientific evidence and developments within the German medical rehabilitation system.

## Results

The primary objective was to develop practical, evidence-based recommendations for promoting PA in movement-based therapies in German medical rehabilitation settings. The subsequent subsections delineate the principal outcomes of these four methodological steps.

### Evidence update (step 1)

The scoping review [23] (Step 1.1) systematically mapped 57 PA promotion concepts implemented by movement-based therapists, including physiotherapists, sports therapists, and exercise physiologists, for individuals with noncommunicable diseases. Of the included records, 77% were empirical intervention studies and 23% were theoretical or conceptual contributions. Most concepts were developed in the contexts of orthopaedics/rheumatology (23%), neurology (21%), and oncology (9%), with 12% representing approaches not limited to specific medical conditions. The analysis identified the use of 66 biopsychosocial assessment tools and 60 distinct behaviour change techniques. Common didactic-methodological principles included tailoring/individualization, active participation, collaborative communication, and patient self-responsibility and independence. This comprehensive examination—addressing intervention components, didactic-methodological strategies, and underlying conceptual frameworks—provides a structured and detailed overview of existing options for PA promotion in movement-based therapeutic settings in the literature. As a scoping review, this component was intended to map and systematise available concepts and did not aim to assess the strength or certainty of evidence.

The overview of 14 systematic reviews [39] (Step 1.2) demonstrated that PA-promoting interventions in movement-based therapy can effectively increase both short- and long-term PA behaviour in individuals with NCDs. A subgroup analysis indicated that certain behaviour change techniques may hold particular promise for enhancing effectiveness. These include prompts and cues, use of a credible source, adding objects to the environment, generalization of the target behaviour, monitoring of behaviour by others without feedback, self-monitoring of outcome(s) of behaviour, graded tasks, behavioural practice/rehearsal, action planning, goal setting (behaviour), behavioural contract, social support (unspecified), and non-specific reward. However, despite these generally supportive findings, definitive conclusions are limited by the low to critically low methodological quality of many included reviews and the resulting low certainty of evidence.

We conducted an analysis of 10 national documents (Step 1.3), comprising four existing practice recommendations in the field of rehabilitation [40–43] and six clinical guidelines [44–49]. The analysis revealed that the promotion of PA is accorded only a minor role in most documents. Most statements within the 10 national documents pertained to therapy content (*n* = 38), therapy goals (*n* = 17), and implementation (*n* = 13), with fewer addressing evaluation (*n* = 8) and assessment (*n* = 6). Particularly significant for the development of our draft recommendations were aspects related to action and coping planning within the therapy content, as well as the formulation of therapy goals in alignment with the national PA recommendations [50].

### Iterative development of recommendations (step 2)

An initial draft comprising 15 recommendations structured across four thematic areas (A–D) was developed (Step 2.1): A – General Recommendations, B – Didactic-Methodological Principles, C – Therapy Content, and D – Interprofessional Collaboration. Further details on the overall development process and the initial draft of the recommendations are available in the final project report (in German) [51].

The inaugural expert workshop (6 h; Step 2.2) was convened on 23 May 2023, in Frankfurt am Main, Germany, to discuss and adapt the initial draft of the practice recommendations. Twelve rehabilitation professionals, including sport therapists and physical therapists (*n* = 5), members of the scientific Working Group “Movement-based therapy” of the German Society of Rehabilitation Sciences (DGRW; *n* = 3), a representative of the German Association for Health Sports and Sports Therapy (DVGS), a psychologist, a physician, and a representative of the DRV, along with five members of the research team, participated. Participants provided feedback on the content, completeness, and practicality of the material and made suggestions for adjustments to the wording, which was collected on a pinboard during a moderated discussion. The group also addressed the feasibility of implementing the recommendations in practice and discussed the specific needs of the target population.

Focus groups were conducted (Step 2.3) at 3 rehabilitation centres: in Oldenburg (26 September 2023), Bad Füssing (2 October 2023), and Bad Krozingen (18 October 2023). Each group comprised 6 to 8 rehabilitation patients with various NCDs. The practice recommendations were simplified for clarity and to underscore their relevance to rehabilitation patients. Overall, the participants found the recommendations appropriate and meaningful, although they offered a few suggestions. Their feedback was considered during the revision process, particularly in refining the content of the background texts, rather than the recommendations themselves. For instance, group therapy was reframed as optional rather than mandatory, and new activity formats were suggested for those unfamiliar with traditional offerings. Key requests included: (a) more options for independent PA during rehabilitation, (b) structured post-rehabilitation follow-ups (e.g., monthly check-ins), (c) greater exercise variety and choice of equipment, (d) individualized support within group therapy, (e) more theory-practice links through frequent educational sessions, (f) voluntary sessions to develop realistic goals with therapists, (g) goal tracking and adjustment over time, and (h) stronger interdisciplinary collaboration for specific needs, such as overcoming mental barriers to physical activity. All original anonymous patient comments (in German) and their integration into the background texts are documented in the final report [51].

The outcomes of Steps 2.2 and 2.3 primarily necessitated structural and linguistic modifications. For instance, in Section C (intervention elements), seven recommendations concerning specific therapy content were formulated and organized in accordance with the PAHCO model [25–27]. The second expert workshop (Step 2.4) was conducted in person on 16 October 2023, in Frankfurt am Main, Germany. The workshop comprised 17 participants, encompassing all intended perspectives from research and practice. The attendees included 12 sport therapists and physical therapists, 2 psychologists, 2 physicians, 1 representative from the German Pension Insurance with a background in sports science, and 5 researchers. Feedback and evaluations concentrated on the linguistic clarity and comprehensibility of the recommendations’ implications for rehabilitation practice, and suggestions for adjustments of the recommendations were discussed and implemented.

The practice recommendations were subsequently revised by the research team. In parallel, the research group developed a structured draft of the corresponding background texts. These supplementary documents provide additional information, concrete examples, and practical guidance to assist movement-based therapists in implementing these recommendations. The revised recommendations and draft structure of the background texts were then re-evaluated by rehabilitation professionals through a written consultation process in Step 2.5 (23 January to 14 February 2024, via MS Forms). During this consultation, every recommendation received strong content approval: for each one, 65–94% of respondents gave full agreement, while the remainder provided conditional agreement accompanied by suggestions for slight improvements.

In response to this input, a consultation version comprising 15 practical recommendations and background texts was formulated. The recommendations are divided into 3 sections: Sections A and B include overarching recommendations that primarily serve as guiding statements rather than concrete practice instructions. Section A addresses the relevance of PA promotion in rehabilitation, cross-cutting aspects such as individualization and interdisciplinary collaboration, and a competence-oriented approach to movement-based therapies within medical rehabilitation (A1 to A4); Section B addresses fundamental didactic-methodological orientations and guiding principles for therapeutic action (B1 and B2); Section C focuses on concrete content-related and methodological implications for key therapeutic processes, such as assessment, goal setting, therapy delivery, and monitoring in PA-promoting movement therapy (C1 to C9). Each recommendation is supported by background text providing a theoretical rationale, illustrative examples, and practical implementation guidance.

This consultation version was subsequently reviewed and approved during a third expert meeting held online on 5 March 2024 (Step 2.6), with 18 participants: 10 sport therapists and physical therapists, 3 members of the DGRW working group on movement-based therapies, 2 psychologists, 2 physicians, and 1 representative from the DRV. This process results in 259 full (“I fully agree”) and 8 conditional approvals (“I partially agree”) for the 15 recommendations. Table 1 summarises the 15 final practice recommendations from Step 2 and the main content of the accompanying background text. A detailed presentation of the background texts can be found in Additional file 1.

**Table 1:** Full text of the practice recommendations and summary of key content of the background texts

Practice recommendations	Summary of the key contents of the background texts
*A: Basic principles for PA promotion in movement-based therapies*	
A1: Support individualized PA behaviour. PA helps rehabilitation clients to improve or maintain their physical and mental functions, as well as their activities and participation (in accordance with the biopsychosocial health model of the ICF). All rehabilitation clients should be supported in being physically active on a regular and long-term basis, within the scope of their individual capabilities and needs.	Rehabilitation as a health strategy to optimise functional capacity; PA as key to improving functional capacity and to achieve rehabilitation goals; promotion of regular PA as central goal in current rehabilitation concepts; PA recommendations, adapted to prerequisites of persons with a non-communicable disease
A2: Empower clients to continue health-enhancing PA. Therapeutic actions in movement-based therapies should aim to empower rehabilitation clients to independently continue engaging in health-enhancing PA. This means using content and methods that support clients in: a) coping with immediate PA-related demands (movement competence), b) controlling their PA towards positive effects on health and well-being (control competence), and c) ensuring regular PA over time (self-regulation competence).	Development of individual skills in dealing with the disease as central element of rehabilitation; competence orientation in the classification of therapeutic services (KTL) of the German Pension Insurance; physical-activity-related health competence (PAHCO) as a resource-oriented goal for movement-based therapy; movement competence, control competence, PA-specific self-regulation competence
A3: Apply a person-centred approach. Movement-based therapy aimed at promoting PA follows the overarching principle of person-centeredness. This includes: (a) considering the individual goals, motives, and preferences related to PA, as well as the physical, psychological, social, and environmental capacities of the rehabilitation clients, (b) using appreciative and activating communication, and (c) promoting an active and participatory role for the clients in the therapeutic process.	Person-centered approach as central principle of modern rehabilitation in line with the ICF’s biopsychosocial understanding of health; explicit consideration of the movement-related experiences, knowledge, preferences and individual perspectives of the rehabilitants
A4: Foster interprofessional cooperation. Promoting PA is an overarching rehabilitation goal that should be addressed through interprofessional collaboration. Movement-based therapists should regularly coordinate with the various professional groups involved in medical rehabilitation regarding PA-related goals and appropriate therapeutic approaches.	Interprofessional collaboration as a basis of a comprehensive biopsychosocial treatment approach; PA promotion as an interprofessional therapy goal; high demand for coordination between professional groups: speaking a common language when it comes to movement; use of all diagnostic information relevant to promoting PA; coordinating general rehabilitation goals with the specific movement-related goals; coordination of the use of behaviour change techniques; interdisciplinary team meetings
*B: Didactical-methodological approach*	
B1: Combine exercise, learning, and experiencing. Movement-based therapy that aims to empower rehabilitation clients for self-determined, health-enhancing PA is based on the principle of linking exercise with learning and experience. This approach takes into account perspectives from exercise and movement science, medicine, education, psychology, and social-ecological frameworks.	Empowerment approach; PA-relatedhealth competence model (PAHCO); action model with systematic linking of practice & training, learning, and experiencing; multidisciplinary perspectives of movement-based therapy: training and movement science, medical, educational, psychological, socio-ecological
B2: Acknowledge previous PA experiences. Movement-based therapy aimed at promoting PA should take into account the previous positive and negative movement experiences of rehabilitation clients. This includes not only their motor abilities, but also their cognitive evaluations and emotional-affective experiences related to PA.	Consideration of prior movement experiences in therapy; role of cognitive and emotional associations in supporting PA; positive and negative experiences as influencers of PA perception; relevance of positive movement experiences and positive affective responses for sustainable PA
C: Therapeutic content	
*C1: Base therapy on biopsychosocial assessment.* Movement-based therapy should be planned and implemented based on an assessment. The assessment can also be used to monitor the course and outcomes of therapy, ideally including follow-up care. To promote PA, the assessment should particularly consider clients´ previous PA behaviour as well as key physical, psychological, social, and environmental factors that influence regular activity.	Definition of assessment and its role in therapy. Content and methods: suggestions for suitable instruments to measure PA behaviour and influencing physical, psychological, social and environmental factors
C2: Set and review PA goals together. PA-related therapy goals should be developed collaboratively and through dialogue with rehabilitation clients. The degree to which these goals are achieved should be reviewed and reflected upon together with the clients.	Goals in rehabilitation and therapy (ICF-based). Content and methods: PA goals targeting capacity, behaviour, self-regulation, and participation; setting SMART goals; Collaborative goal-setting
C3: Improve physical and motor prerequisites. In movement-based therapy, the physical and motor prerequisites for health-enhancing PA should be optimised.	Physical-motor dimensions. Content and methods for enhancing motor abilities (endurance, strength, flexibility, coordination), motor skills (every day and health-related movement skills), and body and movement awareness
C4: Strengthen confidence. Movement-based therapy should strengthen confidence in the independent realisation of health-enhancing PA. This includes the perception of one’s own motor abilities and skills, as well as the enhancement of self-efficacy for specific movement tasks and for maintaining regular PA behaviour.	Movement-related self-efficacy. Content and methods to enhance self-efficacy and body/self-awareness: mastery experiences with graded tasks; vicarious experiences through role models; verbal persuasion and supportive feedback; exposure to alternative, adapted movement options; learning challenging but manageable physical tasks; practicing self-directed, health-enhancing exercises; teaching self-regulation strategies and usage of self-monitoring tools; contrasting movements and proprioceptive exercises.
C5: Provide knowledge and foster control competencies. Movement-based therapy should enable rehabilitation clients to structure their PA in a way that optimises physical and mental health benefits while minimising health risks. This also includes understanding how PA can be individually used to cope with health problems and discomfort.	Biopsychosocial health effects of PA; risk and side effects. Content and methods to optimise health effects and minimise risks: educating on the physical and psychological effects of PA; teaching action knowledge; guiding subjective and objective load monitoring; providing guidance for managing PA during illness flare-ups or health decline; educating on emergency behaviour and risk situations; promoting flexible, individualised goal setting; encouraging reflection on emotional outcomes and affect regulation.
C6: Support motivation through positive experiences with PA. Movement-based therapy should promote rehabilitation clients’ motivation for self-determined, health-enhancing PA. In particular, it should enable and raise awareness of positive movement experiences.	Supporting motivational states and the role of positive movement experiences; positive affect during and after activity as main supporter of sustained PA engagement. Contents and methods: educating on inactivity risks and PA benefits; encouraging recognition of achievements, praise, and self-reward; using FITT-based strategies to promote positive affect; reflecting on emotional outcomes and affect regulation; tailoring activities to individual preferences, motives, and abilities; facilitating peer support and sharing of barriers, strategies, and success stories.
C7: Align PA with individual motives, preferences and prerequisites. Movement-based therapy should enable rehabilitation clients to appraise, choose, and engage in forms of PA that align with their motives, preferences, and individual capabilities.	Movement-related motives and preferences; motivational competence as supporter to select and engage in personally meaningful activities. Contents and methods: identifying individual motives and reflecting on intrinsic vs. extrinsic incentives; providing diverse movement experiences and opportunities to explore different activity types; reflecting on personal preferences and aligning activities accordingly.
C8: Support action planning and coping planning. Movement-based therapy should enable rehabilitation clients to translate their intentions regarding PA into actual, regular behaviour. This includes overcoming internal and external barriers and shielding their PA intentions from competing activities.	Volitional perspective and the PAHCO model; the role of goal setting, planning, and overcoming internal/external barriers to PA. Content and methods: developing activity goals during and after rehabilitation; action and coping planning; creating commitment (e.g., behavioural contracts); (self-)monitoring and feedback on physical function and symptoms; reflecting on goal progress and adjusting plans; encouraging social support
C9: Facilitate continuation after rehabilitation. In movement-based therapy, rehabilitation clients should receive comprehensive counselling about PA opportunities for the period following their medical rehabilitation. This includes information on follow-up rehabilitation programmes, participation in health enhancing PA programme, as well as other suitable PA options.	Post-rehabilitation PA options; appropriate follow-up programmes, local opportunities, and information portals. Content and methods: providing information on local PA options after discharge; facilitating contact with suitable programmes and providers

The list reflects the English translation of the final wording of the recommendations established following the nationwide consultation in Step 3 and the concluding consensus meeting in Step 4. However, 3 recommendations (A1, A3, and C1) were worded slightly differently prior to Step 3 and Step 4 and were subsequently subject to minor revisions based on the feedback received

*Abbreviations*: *PA* Physical activity

Across Steps 2.2, 2.4, 2.6, and the final consensus meeting, participation was partially overlapping but not identical. Core professional stakeholder groups were represented throughout to ensure continuity, while additional experts contributed to specific steps depending on availability and the focus of each meeting.

All supporting materials related to the participatory and consensus-building processes (e.g. meeting agendas, focus group guides, consultation surveys) are documented in the final project report (in German) [51].

### Nationwide consultation and consensus (step 3)

After internal revisions based upon the feedback from Step 2, 15 recommendations, along with their respective background texts were submitted to a nationwide stakeholder consultation. The consultation targeted movement-based therapists and clinical leads (chief physicians) from adult medical rehabilitation clinics across Germany.

A total of 255 people took part in the online survey to comment on the recommendations. Between 145 and 255 responses were received on the individual recommendations. The survey asked respondents for their general agreement and for an assessment of the clarity, completeness and practical relevance of the recommendationsGeneral agreement rates ranged from 86% to 100%, with most of recommendations receiving near-unanimous endorsement. Slightly lower levels of agreement were observed for the following items: A4 – Foster interprofessional cooperation (96%); B2 – Acknowledge previous physical activity experiences (95%); C1 – Base therapy on biopsychosocial assessment (86%); C2 – Set and review PA goals together (96%); and C7 – Align PA with individual motives, preferences, and prerequisites (93%).

**Fig. 2:**
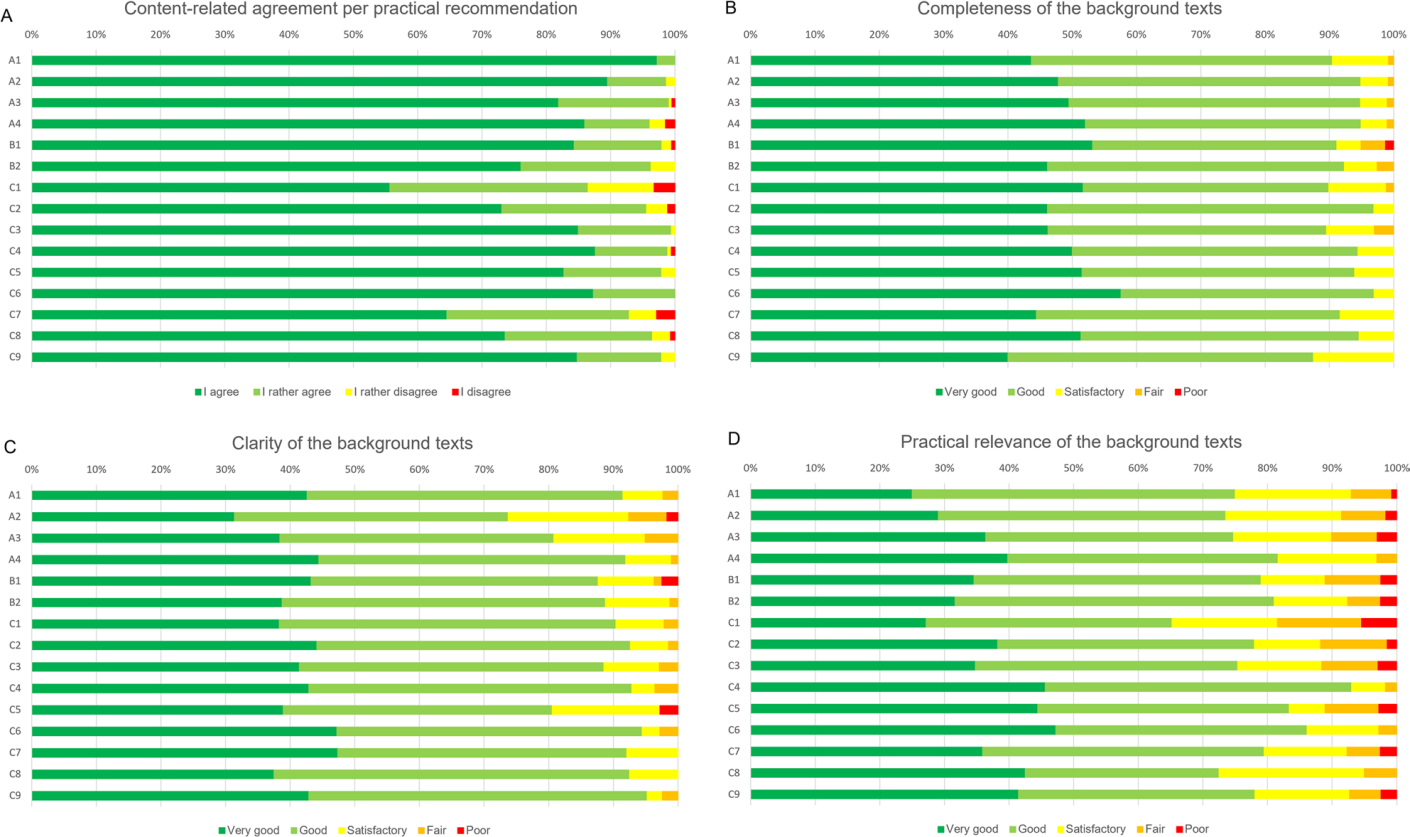
Results of nationwide consultation of practical recommendations and background texts

With regard to the 3 aspects of clarity, completeness, and practical relevance, the background texts were rated by the movement-based therapists with 40–130 ratings per chapter. While clarity and completeness were mostly rated as good or very good, practical relevance was evaluated somewhat more critically. In addition to the quantitative ratings, the participants were invited to provide open-ended qualitative feedback. These free-text responses were primarily used to refine and adapt the background texts. Figure 2 presents the results of the nationwide consultation concerning the evaluations of both practical recommendations and background texts.

### Concluding expert meeting to approve the recommendations (step 4)

Based on the nationwide consultation, selected recommendations with lower agreement (A1, A3, C1) were further refined in a final online consensus workshop held on 13 November 2024. Seventeen participants attended: 5 movement-based therapists, 1 psychologist, 2 physicians, 2 members of the DGRW-working group on movement-based therapy, 2 representatives from DRV, and 5 researchers.

Following a presentation of the consultation results and their implications for the recommendations, the last key adjustments were discussed, particularly those related to C1 (*Assessment*). Participants emphasized the importance of maintaining a standardised, repeated (pre-post) assessment as a benchmark for patient-centred quality assurance. It was agreed that a clearly formulated and binding recommendation would support quality development in this field.

**Fig. 3:**
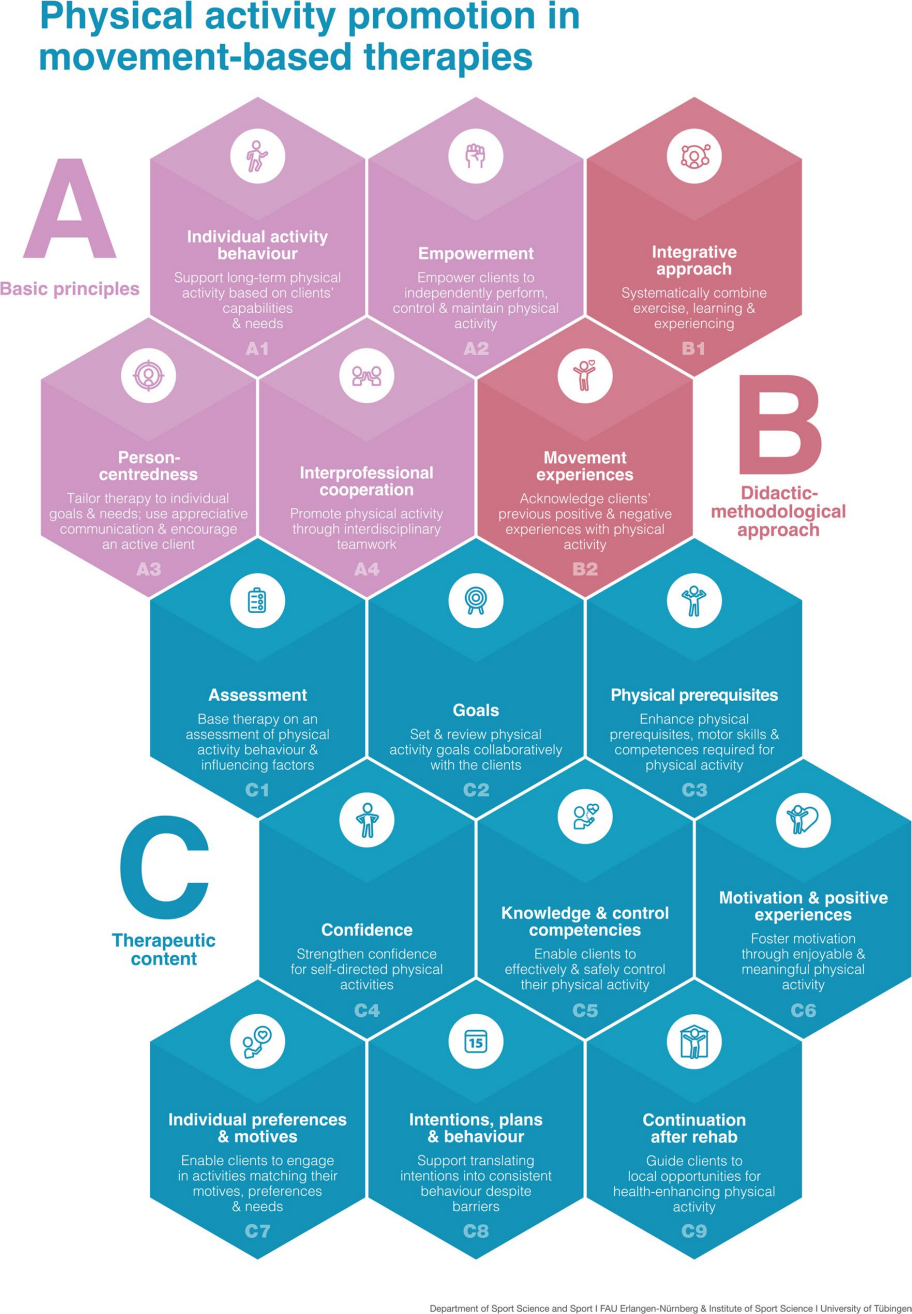
Structured overview of the 15 practical recommendations for physical activity promotion in movement-based therapies

Minor wording revisions were made to recommendations A1 and A3. In the final online vote, both A1 and A3 received 10 votes of “ I completely agree” and 1 “I partially agree.” For recommendation C1, 4 participants responded with “I completely agreed,” 5 with “ I somewhat agree,” and 2 with “I somewhat disagree.” Based on these results and the accompanying discussions, minor linguistic changes were made to the respective recommendations. The final version was then converted into a graphically edited short version (Fig. 3).

## Discussion

The applied co-design process resulted in 15 evidence-informed practice recommendations for promoting PA within movement-based therapy across German medical rehabilitation settings, which received broad consensus during a nationwide consultation. The recommendations cover both the overarching principles of PA promotion in rehabilitation settings and the key therapeutic processes integral to movement-based therapies. These include assessment, goal setting, content and method selection, delivery strategies, and therapy monitoring. Each recommendation is accompanied by background information that outlines the theoretical rationale, offers practical examples, and provides implementation guidance. The results provide clear orientation and practical support for professionals involved in movement-based therapies, including sport therapists, physical therapists, exercise physiologists, and kinesiologists, in integrating PA promotion into routine rehabilitation care, particularly for individuals with NCDs.In Germany, the recommendations address the considerable variability observed in current movement-based rehabilitation practices related to PA promotion [29–31]. They respond directly to the challenges identified in the national survey of movement-based therapies in medical rehabilitation, which include the lack of a clear focus on PA promotion and limited awareness and use of evidence-based and theory-driven interventions [28]. By publishing these practice recommendations, the German pension insurance, the funder of this project, provides a significant impetus for quality development in movement-based therapies within the rehabilitation setting. From an implementation perspective, the recommendations were deliberately developed to be applicable within existing personnel and time resources of medical rehabilitation, in line with the funding framework of the German Pension Insurance. Additional resource requirements related to implementation were examined in a separate needs assessment and will be addressed in subsequent implementation planning.

Promoting PA is a central goal of rehabilitation [52], with long-term success outcomes closely tied to sustained PA adherence. Effective behaviour change strategies should be grounded in sound behavioural theory [53]. Biomedical approaches and utilitarian views that frame PA merely as a means to achieve physical health outcomes have shown limited success [54], as they often neglect the social, emotional, and environmental factors influencing health behaviour [52, 54]. Integrative approaches that address emotional, psychological, and social development alongside physical recovery appear to be more promising. Our recommendations are underpinned by the PAHCO model [26, 27, 55], a holistic, integrative framework explaining and predicting PA behaviour in relation to health and well-being. Rather than focusing solely on meeting guidelines (e.g., 150 min of moderate PA per week), the PAHCO model emphasises empowering individuals to lead physically active lifestyles, thereby optimising both physical and psychological health and well-being. It comprises 3 domains: movement competence, control competence, and PA-specific self-regulation competence. Accordingly, our recommendations encompass both strategies to promote motivation through enjoyable and meaningful PA and volitional approaches that help clients translate intentions into action by overcoming internal and external barriers (self-regulation competence). They also support clients in selecting and managing activities to maximise health and well-being (control competence), as well as enhancing the physical and motor foundations necessary for health-enhancing movement (movement competence). Grounded in PAHCO, these recommendations support a shift from traditional biomedical models towards holistic, patient-centred approaches, aligning with the WHO’s Global Action Plan on Physical Activity [9], which promotes not only lifelong health but also physical literacy, enjoyment, and participation according to individual capacity. The recommendations, especially those addressing person-centredness and the value of positive movement experiences (e.g., A3, B2, C6, C7), also resonate with concepts such as affect-based exercise prescriptions [56], “exercise is recreation, not medicine” [57], and the Four Domains for Development for All (4D4D4All) approach [54].

Internationally, our recommendations complement existing international position statements, such as Alsop et al.’s consensus on core PA promotion competencies for healthcare professionals [11], by offering a concrete action framework specifically tailored to movement-based therapists. While Alsop et al. summarized 11 core competencies (e.g., using a person-centred approach, effective communication, or considering common barriers and facilitators to movement behaviours) [11], our recommendations illustrate how these competencies can be operationalised through specific content and methods. For example, regarding the use of a person-centred approach, our recommendations provide practical guidance on how to align PA promotion with individual motives, preferences, and prerequisites (see Recommendation C7). Similarly, for effective communication, concrete examples are provided for the collaborative and dialogue-oriented formulation of therapy goals, emphasising how movement-related goals can be jointly developed with patients in a meaningful way (see Recommendation C2). Together with the background information, practical examples, and implementation guidance for each recommendation, this equips movement-based therapists with the knowledge and confidence to promote PA effectively, addressing the most commonly reported barriers faced by healthcare professionals, such as insufficient knowledge and skills, and limited perceived success in influencing patient behaviour [15].

In addition to providing concrete intervention content, our recommendations offer broader didactic and methodological guidance (see Section B). This includes, for example, recommendations on how to connect training and practice, learning, and experiential elements in therapeutic settings (Recommendation B1), drawing from interdisciplinary perspectives in exercise science, human movement science, medicine, education, psychology, and socioecological frameworks. A particular focus is placed on acknowledging and incorporating patients’ previous positive and negative movement experiences into the therapeutic process (Recommendation B2), thereby supporting more meaningful and individualised behavioural change strategies.

While concrete, practice-oriented recommendations have been available for other professional groups, such as physicians, who can draw on resources like “Moving Medicine“ to support patient counselling on PA [12], comparable guidance for movement-based therapists has been lacking until now. Although developed within the context of the German rehabilitation system, these recommendations may also serve as a valuable resource for movement-based professionals in other healthcare systems. They can be adapted to reflect national needs and contextual factors, for example, by applying the GRADE-ADOLOPMENT framework [58], a structured approach that supports the adoption, adaptation, or de novo development of recommendations by systematically integrating existing guideline evidence with contextual considerations such as feasibility and acceptability.

Most of our recommendations received high levels of agreement during the co-design and consultation phases of development process. However, a few recommendations sparked controversial discussions. One particularly debated recommendation was the use of a standardised biopsychosocial assessment (Recommendation C2). While workshop participants strongly emphasised its relevance for individualised, high-quality therapy, the broader consultation revealed more critical views regarding its feasibility and practical relevance. This discrepancy likely reflects the current lack of validated multidimensional PA-related assessments in rehabilitation practice, both nationally [30] and internationally [23]. Future research and development efforts should therefore focus on creating practical, psychometrically sound tools that can support needs-based planning and evaluation of PA promotion in movement-based therapies. Another recommendation that prompted discussion was Recommendation A1, which addresses the support of individualised PA behaviour as a general guiding principle. Here, discussions focused on the use of ICF terminology—particularly the concept of functioning and its links to activities and participation—and its alignment with the national physical activity recommendations for people with noncommunicable diseases. These discussions concerned conceptual clarity and terminology rather than fundamental disagreement with the recommendation itself.

### Strengths and limitations

A key strength of the PRO-BT project lies in its systematic and evidence-informed co-creation approach, incorporating principles of co-design [32] through the active collaboration of academic and non-academic stakeholders in developing the recommendations. Drawing on current evidence and grounded in the realities of clinical practice, the process ensured strong stakeholder engagement and acceptability, as reflected by the high agreement rates during the nationwide consultation. The structured, multiphase development, based on methods from guideline development, enabled repeated stakeholder feedback, ensuring that the evidence was translated into practically feasible recommendations. In addition, facilitators and barriers to implementation, as well as perceived support needs, were systematically assessed in a separate needs assessment conducted at the end of the PRO-BT project; the results are currently being prepared for publication.

At the same time, the project does not fulfil the criteria of full co-production or integrated knowledge translation [59], as stakeholders were not involved as research partners across all stages of the research process, including the initial formulation of the research question and the first drafting of the recommendations. Consistent with recent conceptual distinctions, the project is therefore more appropriately described as an applied co-design process embedded within a broader co-creation framework, rather than as full co-production. Although the overall development followed a participatory and co-design-oriented approach [32, 33], a limitation is that the initial draft of the recommendations was developed by the academic research team without direct stakeholder involvement. From a strict co-production perspective, earlier engagement of stakeholders in the initial framing and drafting stages would have been necessary to ensure shared decision-making and power-sharing throughout the process.

The involvement of rehabilitation patients was intentionally limited to providing feedback on draft recommendations and background texts, rather than participating directly in drafting the recommendations. This reflects the intended target users of the recommendations, which are movement-based therapists rather than patients, as well as practical constraints related to patients’ limited availability during medical rehabilitation. Nevertheless, this limited involvement of people with lived experience represents an important limitation when considering the depth of participatory engagement.

In addition, participants in the co-design workshops and consensus meetings were primarily rehabilitation professionals and researchers with substantial expertise in PA promotion and a generally positive orientation towards the topic. Movement-based therapists without academic backgrounds or with different therapeutic foci were underrepresented, which may have constrained the diversity of professional perspectives included.

Overall, the PRO-BT process may serve as a methodological example of good practice for co-design-oriented guideline development in complex health system contexts.

A notable limitation of the co-creation process is that the participants involved in the co-design workshops tended to be academically educated and positively predisposed toward PA promotion. Movement-based therapists without academic backgrounds or with different therapeutic foci were underrepresented, potentially limiting the breadth of perspectives included.

### Dissemination and future directions

The formal adoption and publication of these recommendations by the German Pension Insurance represent an important first step. However, effective implementation will require targeted dissemination efforts and the provision of supportive structures. To this end, the PRO-BT project also identified specific areas in which therapists require additional resources and support, such as educational media, materials, and implementation tools. In parallel, and in line with the approach taken by Wingood et al. [20], we are investigating the determinants of successful implementation of PA promotion in movement-based therapies as part of a related study [60]. This research will form the basis for developing concrete implementation strategies for the recommendations. Future research should focus on evaluating the degree to which the recommendations are adopted in clinical practice and their measurable impacts on therapeutic processes and patient outcomes. These insights will inform subsequent phases of dissemination, capacity building, and the sustainable integration of PA promotion into routine rehabilitation care.

## Conclusion

The interdisciplinary co-creation process, grounded in co-design principles and reflecting the integration of scientific evidence and practitioner and rehabilitant perspectives, led to the development of 15 practice-oriented recommendations aimed at promoting PA within movement-based therapies in the context of medical rehabilitation. The recommendations provide evidence-informed and practically relevant guidance for movement-based therapists (such as physical therapists, exercise therapists, sports therapists, exercise physiologists, and kinesiologists) that covers both overarching principles and specific therapeutic processes. Based on the PAHCO model, the recommendations support a holistic, patient-centred approach to PA behaviour. By integrating scientific evidence with the practitioner and patient perspectives, the PRO-BT project contributes to strengthening the role of rehabilitation professionals, especially movement-based therapists, in supporting sustainable changes in PA behaviour in people with NCDs.

## Supplementary Information

Supplementary Material 1. Additional file 1 EnglischTranslation Recommendations and Background

## Data Availability

Nearly all datasets used to develop the practice recommendations are directly available within the appendix of the final German-language report . Additional datasets beyond those included therein can be provided upon reasonable request to the corresponding author.

## Abbreviations

NCDs: Noncommunicable Diseases
PA: Physical activity
PAHCO: Physical Activity-related Health Competence
