# Supplementary material for: Practice recommendations for physical activity promotion in exercise therapy, physical therapy, and other movement-based therapies: a co-design project (PRO-BT) in German medical rehabilitation
Source: BMC Sports Sci Med Rehabil. 2026 Mar 4;18:175. doi: 10.1186/s13102-026-01638-4 (PMC13063569; doi:10.1186/s13102-026-01638-4)
Supplement: Supplementary file 1 — Supplementary Material 1. Additional file 1 EnglischTranslation Recommendations and Background [file 13102_2026_1638_MOESM1_ESM.pdf]

Physical Activity Promotion in Medical Rehabilitation

--

Practical recommendations for movement-based therapies[1]

Prof. Dr. Klaus Pfeifer

Prof. Dr. Gorden Sudeck

PD Dr. Wolfgang Geidl

Andrés Jung, M.Sc. PT

Leon Matting, M.Sc. Sportwiss.

[1] English translation of the final version of the recommendations and background texts (12.08.2025), see https://www.sport.fau.eu/das-institut/forschung/bewegung-und-gesundheit/abgeschlossene-forschungsprojekte/pro-bt/

Table of Contents

Background ... 3
Aims of the Practical Recommendations ... 3
Cross-Indication Orientation ... 4
Development of the Practical Recommendations ... 4
Structure of the Practical Recommendations ... 4
Glossary ... 6
Practical Recommendations Part A: Basic Principles of Physical Activity Promotion in Movement-Based Therapies ... 8
A1: Supporting individualized physical activity behavior ... 8
A2: Empowering rehabilitants ... 10
A3: Person-centered approach ... 13
A4: Interdisciplinary Cooperation ... 16
Practical Recommendations Part B: Didactic-methodical implementation ... 18
B1: Linking training and practice, learning and experience ... 18
B2: Importance of prior movement experiences ... 21
Practical Recommendations Part C: Concrete Therapy Design ... 23
C1: Assessment ... 23
C2: Physical activity-related therapy goals ... 27
C3: Improve physical and motor prerequisites ... 32
C4: Strengthen confidence ... 34
C5: Provide knowledge and foster control competence ... 37
C6: Support motivation through positive experiences with physical activity ... 43
C7: Align physical activity with individual motives, preferences and prerequisites ... 48
C8: Support action planning and coping planning ... 50
C9: Facilitate continuation after rehab ... 53

Background

Movement-based therapy [2]is a substantial component of the medical rehabilitation provided by the German Pension Insurance (DRV). Across all indication areas, movement-based therapy services account for the largest share of all services provided as part of medical rehabilitation (Brüggemann et al. 2018). One central objective of movement-based therapy is to promote physical activity based on the development of individual competencies for initiating and maintaining a physically active lifestyle (Deutsche Rentenversicherung 2014).

Promoting physical activity as a goal of medical rehabilitation presents both a promise and a challenge. The promise presented by promoting physical activity lies in the sustained benefits provided by the comprehensive health-promoting effects of regular physical activity, which have been proven for a large number of non-communicable diseases (Pedersen and Saltin 2015; Dibben et al. 2024). However, physically inactive lifestyles are widespread, especially among people with non-communicable diseases (Brawner et al. 2016; Barker et al. 2019; Sudeck et al. 2021; Marks-Vieveen et al. 2024), highlighting the importance of physical activity promotion. The challenge lies in the demanding task of promoting the physical activity behaviour of rehabilitation patients in a sustainable manner..

Measures to promote physical activity in movement-based therapy, as with rehabilitation as a whole, are based on a biopsychosocial understanding of health and sickness – an understanding which also provides the basis for the functional health model and the International Classification of Functioning, Disability and Health (ICF) of the World Health Organization (WHO) (WHO 2001). This holistic, biopsychosocial approach makes it possible to include the many personal and environmental factors that influence physical activity behavior. This framework allows for movement-related attitudes, beliefs and knowledge as well as social and structural environments surrounding the home to be taken into account alongside the physical conditions of the rehabilitants.

Building on this, competence-oriented intervention approaches in movement-based therapy aim to enable rehabilitants to develop and maintain a physically active lifestyle, develop and maintain a physically active lifestyle, strengthen health resources and cope better with their disease (Deutsche Rentenversicherung 2014). Structured as such, movement-based therapy is characterized by a systematic combination of training and practicing, learning and experiencing (Pfeifer and Sudeck 2020). Movement-based therapy that promotes physical activity means learning and advising, motivating and discussing, practicing and training, supporting and planning, playing and laughing, experiencing and reflecting. Similar biopsychosocially-oriented concepts are increasingly being discussed and demanded in healthcare around the world, sought after to provide sustainable access to the effects of movement-based therapy and the positive health effects of physical activity (Dean 2009; Dean et al. 2011; Elvén et al. 2015; McGrane et al. 2015; Lein et al. 2017; Rethorn et al. 2022).

Aims of the Practical Recommendations

These practical recommendations for promoting physical activity are intended to help improve the planning, implementation and quality assurance of movement-based therapies (Geidl et al. 2022). They aim to support rehabilitation teams and movement-based therapists in the design, implementation and evaluation of physical-activity-promoting movement-based therapy. This applies especially to the following core processes and elements (Werle et al. 2006): individual assessment;

[2] Movement-based therapists and the therapies they provide are often described using various terms. In this document, we include physical therapy, physiotherapy, exercise therapy, and sport therapy under the umbrella of *movement-based therapy*. A detailed rationale can be found in Matting et al. 2025.

formulation of therapy goals; selection of content, methods and media; therapy implementation and therapy monitoring.

Cross-Indication Orientation

Promoting physical activity is an important overarching rehabilitation goal. All rehabilitants should be supported in being physically active on a regular and long-term basis according to their needs and abilities. The practical recommendations therefore relate to all indication areas of medical rehabilitation and are therefore formulated across all indications. They address central and cross-indication relevant problems of rehabilitants (e.g. reduced fitness, movement-related insecurity and fears or avoidance beliefs, low motivation, etc.) and enable the targeted use of suitable therapy elements.

Development of the Practical Recommendations

The practical recommendations were developed in a multi-stage process based on methodologies for systematic guideline development. In addition to taking current scientific evidence into account, the inclusion of the expertise of actors intimately involved in rehabilitation practices as well as the perspectives of rehabilitants greatly influenced the recommendations.

Two systematic literature reviews on the concepts (Jung et al. 2023, Matting et al. 2025) and the effect (Jung et al. 2024) of physical-activity-promoting movement-based therapies as well as the optimization potential of movement-based therapies in medical rehabilitation identified in a previous project (Geidl et al. 2022) served as a starting point. A draft version of the practical recommendations developed on this basis was discussed and further developed in two expert workshops together with a total of 26 practical and academic rehabilitation experts. Feedback from three focus groups with a total of 21 rehabilitants was also incorporated into the development process. In a subsequent online survey process and a third online meeting with academic and practical actors, the practical recommendations were agreed upon and converted into a consultation version with background texts. Finally, the practical recommendations and background texts were made available to the leading movement-based therapists and chief physicians of all (approx. 1200) specialist departments of medical rehabilitation for adults for evaluation and comment in a nationwide consultation process. Based on subsequent feedback, further adjustments were made, and a final consensus was reached in the conclusive expert workshop.

Structure of the Practical Recommendations

The practical recommendations are divided into three sections (see Figure 1).

- Part A contains overarching recommendations on the importance of promoting physical activity and the competence-oriented focus of movement-based therapies within medical rehabilitation (recommendations A1 to A4).
- Part B addresses basic methodological and didactic orientations and operational principles (recommendations B1 to B2).
- Part C addresses specific content-related and methodological implications for central therapeutic processes, such as assessment, therapy goals, therapy implementation and the monitoring of physical-activity-promoting movement-based therapies (recommendations C1 to C9).

Each of the practical recommendations is accompanied by an explanatory background text. These background texts contain in-depth information and, particularly in Part C, examples of how to promote the individual skills of rehabilitants in the context of movement-based therapeutic practice.

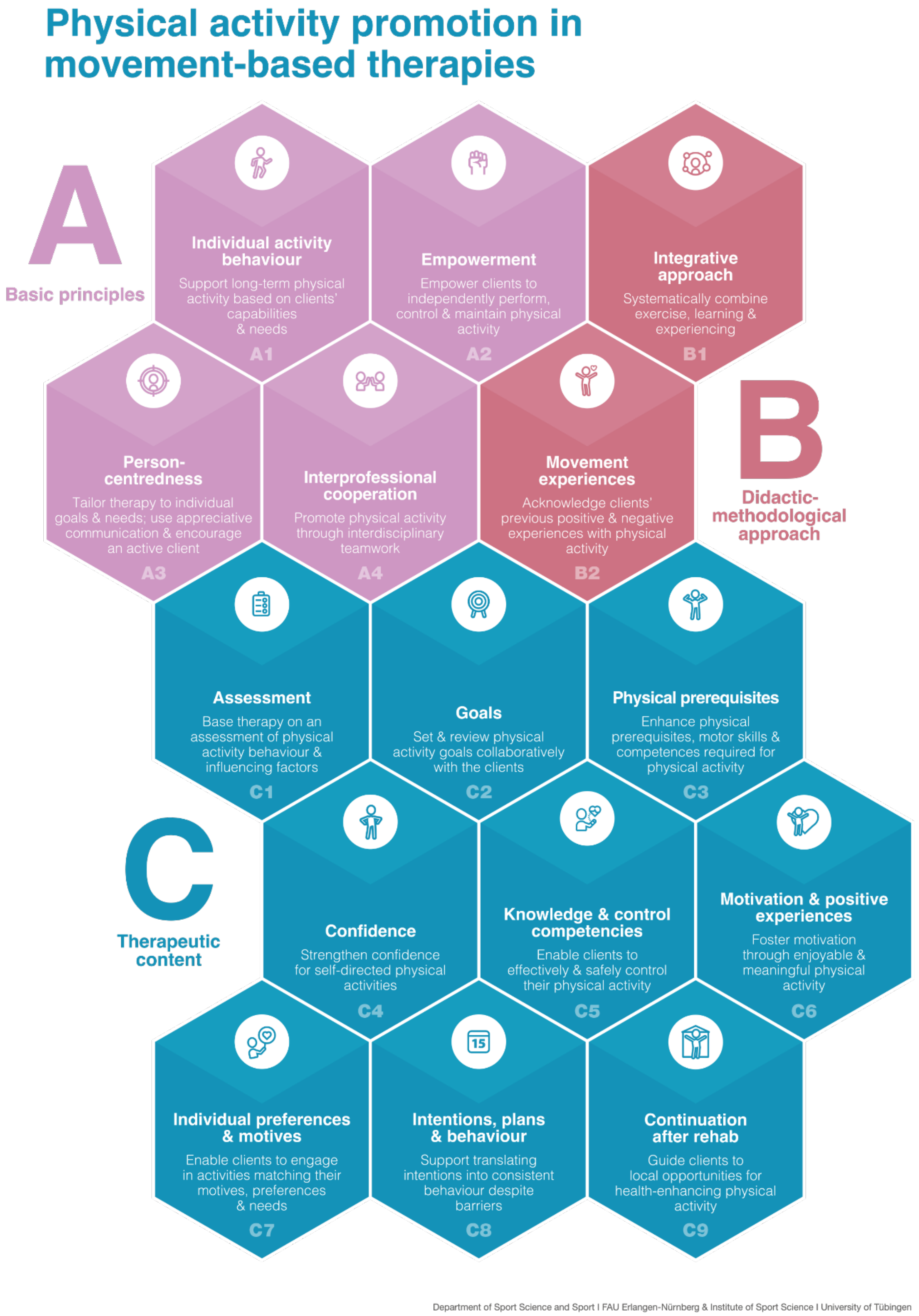

Figure 1: Content structure of the practical recommendations for promoting physical activity

Glossary

Term	Description
Affective attitudes	Emotional reactions or feelings towards a certain behavior, e.g. enjoyment of exercise or aversion to physical activity.
Affective-emotional	Refers to feelings and emotions that influence an action or decision.
Physical-activity-related health competence (PAHCO)	Interplay of cognitive and motor skills and abilities that are necessary to be able to carry out health-enhancing physical activities, as well as the associated motivational, volitional and social readiness and abilities to successfully and critically embed health-enhancing physical and sporting activity in a variety of everyday situations.
Movement competence	Motor skills and abilities, including body and movement perception and sensorimotor control necessary for coping with the motor requirements of health-enhancing physical activity. People with a high level of movement competence are able to perform a variety of health-enhancing physical and sporting activities
Effect knowledge	Knowledge about the effects and benefits of physical activity on health and well-being.
External motivation	Motivation that is determined by external factors such as rewards, pressure or social recognition.
Action knowledge	Practical knowledge on how to implement a certain (physical activity) behavior in everyday life.
Identified motivation	Form of motivation in which a behavior is recognized as personally important and is therefore carried out.
International Classification of Functioning, Disability and Health (ICF)	WHO classification system for describing health and health-related conditions.
Internal motivation	Motivation that arises from one's own interest or pleasure in the action itself.
Cognitive-rational attitudes	Attitudes towards a behavior based on considerations and evaluations (e.g. “exercise is healthy”).
Physical activity[3]	Any movement generated by skeletal muscles that leads to an increase in energy expenditure. Physical activity covers the entire spectrum from sporting activities to everyday leisure activities, all forms of self-locomotion, housework, gardening etc.
Physically active lifestyle	A lifestyle that integrates regular physical activity into everyday life.
Motivation	Inner drive that initiates, directs and maintains behavior.
Motivational behavior determinants	Factors that influence the development of motivation for a certain behavior (e.g. attitudes, goals).
Self-regulation competence	The ability to set personal goals for health-enhancing physical activity based on intrinsic motivation and then implement these goals regularly in everyday life, even when obstacles (e.g. bad

[3] The terms physical activity and movement are often used interchangeably in the text.

	weather) and competing intentions (e.g. watching favorite TV show) arise. This sub-competence of the PAHCO builds on motivational and volitional determinants of physical activity behavior (e.g. attitudes, motivational structure, self-efficacy).
Self-efficacy / self-efficacy beliefs	Conviction of being able to perform a certain action successfully.
Control competence	The ability to manage physical exertion based on knowledge of physical and sporting activities (e.g. knowledge of training methods, exertion dosage, effects on health and well-being) and with the help of refined bodily and movement awareness (e.g. movement execution, heart rate, perceived exertion, affective state), thereby avoiding over- or under-exertion as well as discomfort or inappropriate physical exertion.
Volition	Will processes that are necessary to successfully carry out a motivated action.
Volitional behavioral determinants	Factors that influence the implementation of behavior, e.g. action planning or dealing with barriers.

Practical Recommendations Part A: Basic Principles of Physical Activity Promotion in Movement-Based Therapies

A1: Supporting individualized physical activity behavior

(A1) *Physical activity helps rehabilitation clients improve or maintain their physical and mental functions, as well as their activities and participation (in accordance with the biopsychosocial health model of the ICF). All rehabilitation clients should be supported in being physically active on a regular and long-term basis, within the scope of their individual capabilities and needs.*

Background

Rehabilitation as a health strategy aims to optimize the functional capacity of people living with a disease in accordance with the biopsychosocial health model of the ICF (Deutsche Rentenversicherung Bund 2009). This includes reducing the impact of the disease on the everyday lives of those affected and supporting them to remain as independent as possible despite limitations and to maintain their professional functions and roles in family and society to the best of their ability (ibid.). This includes slowing down the progression of the disease, reducing any impairments of functions and activities that have already occurred and preventing long-term disadvantages in terms of social participation (ibid.).

Physical activity is key to improving functional capacity and achieving rehabilitation goals. People with a wide range of diseases benefit from physical training and a physically active lifestyle. For the non-communicable diseases that dominate rehabilitation, such as cardiovascular diseases, cancer, chronic respiratory diseases, type 2 diabetes mellitus, depression, back pain and osteoarthritis, physical activity improves the course of the disease, the symptoms and the risk of secondary and concomitant diseases as well as the risk of mortality (Pedersen and Saltin 2015; U.S. Department of Health and Human Services 2018; Geidl et al. 2020b; Dibben et al. 2024).

Physically inactive lifestyles are particularly prevalent among people with non-communicable diseases (Brawner et al. 2016; Barker et al. 2019; Sudeck et al. 2021; Marks-Vieveen et al. 2024). Therefore, the promotion of regular physical activity is a central goal in current rehabilitation concepts seeking to exploit the potential for achieving long-term ICF-based rehabilitation goals (i.e. maintaining bodily functions and structures, activities and participation of the rehabilitants).

The National Recommendations for Physical Activity and the Physical Activity Promotion (Rütten and Pfeifer 2017) provide a general point of reference for the promotion of physical activity. For people with a non-communicable disease - as well as for the adult population as a whole – the following is recommended:

- at least 150 minutes/week of aerobic physical activity at a moderate intensity (e.g. 5x30 minutes/week) or
- at least 75 minutes/week of higher-intensity aerobic physical activity or
- an appropriate combination of aerobic physical activity of both intensities and
- additionally performing muscle-strengthening exercises twice a week.

However, not every rehabilitant will always be able to fulfill these general movement recommendations. For some individuals, and in certain life situations, a lower level of exercise may be more adequate. Accordingly, the recommendations also call to take into account the uniqueness of each person with a non-communicable disease and their potential adaptation needs (Pfeifer and Geidl 2017; Geidl et al. 2020a):

- Adults with a chronic disease should be as active as their current circumstances allows during periods when they are unable to exercise as recommended (e.g. due to disease severity, symptoms or physical functioning).

- When starting a physically active lifestyle or a physical training program, they should work with someone in a related physical activity profession to individually adjust the dose of exercise (i.e. type of exercise, exercise intensity, duration and frequency) to ensure the safety and effectiveness of physical activity.

- In phases of disease progression, lack of disease control or deterioration of health, they should seek professional advice from healthcare professionals, as, for example, changes in physical activity or even a break in activity may be necessary.

A2: Empowering rehabilitants

(A2) *Therapeutic actions in movement-based therapies should aim to empower rehabilitation clients to independently continue engaging in health-enhancing physical activity. This means using content and methods that support clients in:*

a) coping with immediate physical activity-related demands (movement competence),

b) controlling their physical activity toward positive effects on health and well-being (control competence), and

c) ensuring regular physical activity over time (self-regulation competence).

Background

Physical activity-related health competence (PAHCO) as a goal of movement-based therapy

An important objective of rehabilitation is to enable rehabilitants to deal with their disease appropriately and independently. Therefore, the development of individual skills in dealing with the disease is a central element of rehabilitation.

Followingly, a competence-oriented approach is also important for movement therapy. In the current version of the classification of therapeutic services (KTL) of the German Pension Insurance (Deutsche Rentenversicherung 2014), movement-related objectives are described. Aspects of competence orientation are linked to the promotion of physical activity and the management of health impairments:

> "Sport therapy services focus explicitly on physical as well as emotional, social and behavioral objectives. Focus is placed on the development of individual competences that promote the initiation and maintenance of a physically active lifestyle and thus make the effects of physical activity sustainable while additionally contributing to a positive coping process in dealing with chronic diseases and restrictions on participation." (ibid., p. 45)."

Against this background, physical-activity-related health competence (PAHCO) can be considered a resource-oriented goal for movement-based therapy measures (Pfeifer et al. 2013; Pfeifer and Sudeck 2022). This refers to individual competencies that are important for initiating and maintaining health-effective physical activity behavior (Sudeck et al. 2020). Accordingly, when formulating the goals of individual movement-based therapy services, the KTL of the German Pension Insurance refers to PAHCO or its sub-competencies (i.e. movement competence, control competence, self-regulation competence). These three sub-competencies also form the content-related reference points of practice recommendation A2.

What exactly is physical-activity-related health competence (PAHCO)?

PAHCO comprises three sub-competencies, which can be derived from the personal prerequisites for independent, health-enhancing movement (Pfeifer and Sudeck 2016; Pfeifer and Sudeck 2022):

Movement competence for coping with the immediate motor requirements of everyday physical activities (e.g. cycling or climbing stairs) and sporting activities (e.g. Nordic walking, jogging, muscle-strengthening exercises, ball games). Movement competence is made up of motor skills and abilities as well as body and movement perception and sensorimotor control (Pfeifer et al. 2013). People with a high level of movement competence are able to carry out a wide range of healthy physical and sporting activities.

Control competence for the adequate design and control of physical stress, such that biopsychosocial health gains are optimized and health risks minimized. This area of competence is primarily concerned with the use of information from processes of body and movement perception as well as the simultaneous targeted use of knowledge for independent control of physical stress, garnering a positive effect on health and well-being. Concretely, people with a high level of control competence have knowledge of actions and effects (e.g. knowledge of training methods, exercise dosage and the effects of physical activity on health and well-being; see also recommendation C5). In addition, they can control and manage load with the aid of well-developed body and movement perception (e.g. in relation to movement execution, heart rate, feeling of exertion or affective state) and in this way avoid incorrect loads and over- or under-exertion.

Self-regulation competence to ensure the necessary regularity of physical and sporting activity that is beneficial to health. People with a high level of self-regulation skills are able to set personal goals for healthy activities on the basis of intrinsic motivation. They can also shield their goals from competing interests (e.g. favorite TV series) and achieve them when barriers arise (e.g. bad weather). This area of competence includes motivational and volitional behavioral determinants, which can be described in close alignment with psychological models of health behavior (e.g. Pfeffer and Wagner 2020). The model emphasizes positive cognitive-rational attitudes (e.g. perceived benefits), affective attitudes (e.g. enjoyment of exercise), a supportive motivational structure and self-efficacy beliefs as characteristics that promote action.

Figure 1: Model of physical activity-related health competence (adapted from Pfeifer et al. 2013)

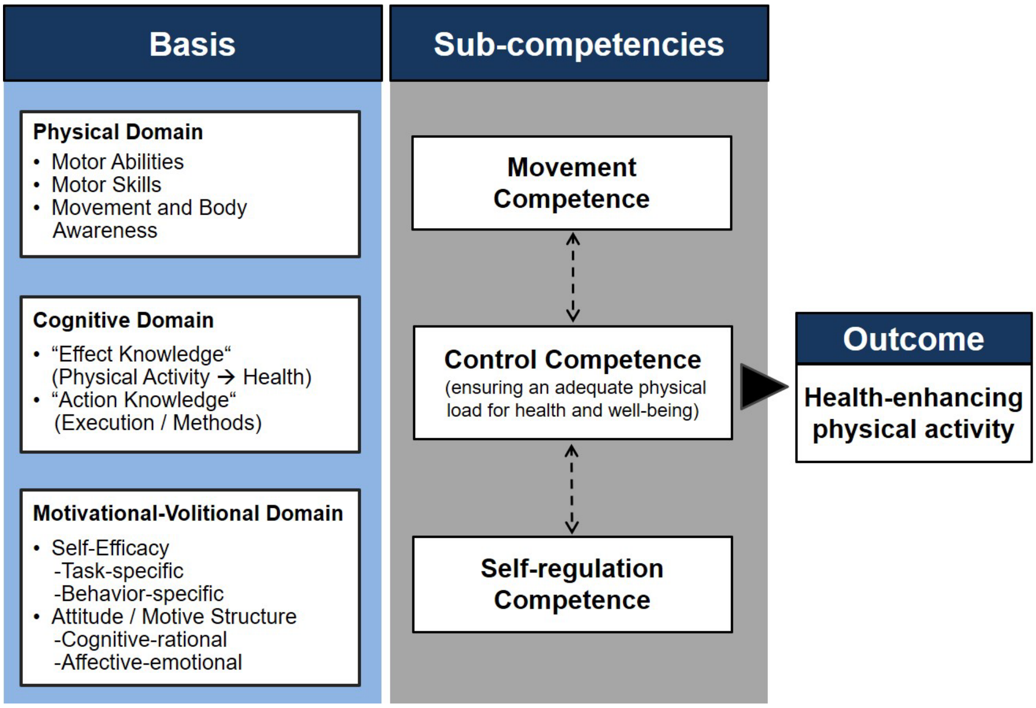

The skills described are neither purely motoric, nor purely cognitive or purely motivational. Rather, competent behavior requires an interplay of various basic elements (see Fig. 1), all important for the respective "function" of the sub-competencies. The respective importance of the sub-competencies is briefly described in practical recommendation A2.

This basic idea is illustrated in the following table using the example of independent strength training (Sudeck et al. 2020).

Example: Promoting physical-activity-related health competence for independent strength training
- *Movement competence:* Competent movement execution in strength training (on equipment or in exercises with your own body weight) is based on practicing (motor learning) the respective forms of exercise. This creates the motor prerequisites for people to be confident in performing the movement (task-specific self-efficacy) while simultaneously not being inhibited by fears when performing the movement.
- *Control competence:* A high level of control competence requires knowledge of the effects and methods of health-oriented strength training (knowledge of effects and actions). People who have been taught this knowledge can optimize the health benefits of their physical and sporting activities and minimize health risks. This requires awareness of one's own psychophysical state and the processing of body signals under stress (e.g. local muscle fatigue as an important adaptation stimulus or dysfunctional posture with increasing fatigue) as well as the ability to adapt individual load dosage (e.g. depending on the training goal and perception of exertion).
- *Self-regulation competence:* The regular integration of independent strength training into everyday routines requires a flexible interplay of motivational and volitional processes. Motivational processes refer to the inner motivations, desires and goals that stimulate a person's behavior. Volitional processes refer to a person's ability to translate their motivation into actual actions. The communication of knowledge elements (e.g. knowledge about the effects of strength training or about volitional techniques for self-observation of training) can be closely linked to the development of a positive attitude towards strength training and a high level of self-efficacy. In terms of motivational competence (Rheinberg and Engeser 2010), it is advantageous to become familiar with the variety of possibilities for physical training and its different stimuli and requirements. In this way, a comparison can be made with one's own motivational and goal structure and, ideally, the intrinsic choice of strength training can be pursued in accordance with one's own goals and values.

A3: Person-centered approach

(A3) *Movement-based therapy aimed at promoting physical activity follows the overriding principle of the person-centered approach. This includes:*

a) considering the individual goals, motives, and preferences related to physical activity, as well as the physical, psychological, social, and environmental capacities of the rehabilitation clients,

b) using appreciative and activating communication, and

c) promoting an active and participatory role for the clients in the therapeutic process.

Background

The person-centered approach is a central principle of modern rehabilitation approaches (Farin 2014; Kayes and Papadimitriou 2023). This approach not only considers the disease of the respective rehabilitants and its direct consequences, but also considers the individual personal and environmental context factors in line with the ICF's biopsychosocial understanding of health. A person-centered rehabilitation approach explicitly takes into account the experiences, knowledge, preferences and individual perspectives of the rehabilitants (Farin 2014). Individual needs form the starting point for the selection and use of therapeutic interventions.

To expand upon aspects a), b) and c) of recommendation A3:

Aspect a) *"the consideration of the individual movement-related goals, motives and preferences as well as the physical, psychological, social and environmental possibilities of the rehabilitants"*

Individual movement-related goals, motives and preferences play a key role in promoting physical activity as part of medical rehabilitation. The assessment of relevant psychological factors (see assessment in recommendation C1) and the development of individual movement-related goals (see specific content and methods in recommendation C2) can serve as the basis for planning movement-based therapy. Suitable movement-based therapy content and methods can be selected for the period of medical rehabilitation, accounting for individual motivations and preferences for movement (see specific content and methods in recommendation C8) as well as physical and psychological capabilities (see assessment in recommendation C1).

In continuing regular physical activity following medical rehabilitation, rehabilitants' social and environmental parameters should be accounted for. These play an important role in the development of concrete courses of action and coping strategies for physical activity behavior as well as in advising on exercise offers and opportunities (see recommendation C9).

Aspect b) *"appreciative and encouraging communication"*

Appreciative and encouraging communication promotes a trusting relationship between the rehabilitant and their respective therapists (Härter and Dirmaier 2022). A high level of trust facilitates good cooperation and helps to achieve therapy goals more effectively. Appropriate communication also creates a positive therapeutic environment, improving the well-being of the rehabilitant and increasing motivation to participate in therapy (Laitakari and Asikainen 1998; Elsman et al. 2014).

In person-centered care, the members of the therapeutic team see themselves not only as researchers and care providers - they also engage in "real" interaction with the rehabilitants. According to Farin et

al. (2014), this includes a) asking questions, b) responding to queries, c) exploring unspoken concerns and d) engaging in an exchange about participation in the treatment process.

Examples of the implementation of appropriate interaction and communication within the framework of movement-based therapy concepts can be found in Table 1.

Table 1: Examples of content and methods of appropriate interaction and communication.

Conducting open, informal conversation	Encourage open and informal conversations about movement, additionally bolstering confidence-building and relationship-building (Laitakari and Asikainen 1998).
Listening and empathetic behavior	Actively listen to the rehabilitant on the topic of movement and empathize with their perspective (Elsman et al. 2014).
Personalization ("Tailoring")	Providing opportunities for rehabilitants to have personal questions about movement and exercise answered and to receive instructions and feedback according to their individual progress (Laitakari and Asikainen 1998; Elsman et al. 2014).
Cooperation with cllients	Discuss assessment results on physical activity behavior with the rehabilitant, explore their willingness to change and develop a therapy plan (Stevens et al. 2018).
Activating communication	Encouraging the expression of ideas to determine movement goals and planning (Elvén et al. 2015; Stevens et al. 2018).
Supporting decision-making	Communicating precise, evidence-based information (e.g. on effect knowledge and practical knowledge in the context of movement) in understandable language to facilitate informed decision-making (Moore and Kaplan 2018).
Supportive language	Use of supportive forms of communication and conversational topics on movement to strengthen the rehabilitant's feelings of competence, autonomy and connectedness (Elsman et al. 2014; Moore and Kaplan 2018)

Aspect c) *"the active and participatory role of the rehabilitants"*

The active participation of rehabilitants is important for high-quality rehabilitative therapy (Härter and Dirmaier 2022). Within movement-based therapy, this applies to an active and co-determining role in various treatment situations, e.g. in the development of therapy goals (see recommendation C2), in therapy selection and planning, in training content and information (see recommendation C5), in experiencing and reflecting on different physical activities (see recommendation C6) or in the independent control of movement and training (see recommendation C5). The movement-based therapy group situation also offers a wide range of activating opportunities and potential interactions between the rehabilitants (e.g. group discussions, joint practice and training, movement-related exchange of experiences).

Facilitating co-determination means, for example, that the members of a person-oriented therapeutic team

- inform the rehabilitant transparently and comprehensively,
- discuss with the rehabilitant the extent to which he or she wishes to be involved in treatment planning,
- respond appropriately to the experiences and prior knowledge of the rehabilitant (Farin 2014; Moore and Kaplan 2018)

- aim to gradually increase the rehabilitants' personal responsibility for their physical activity and thus reduce their dependence on therapeutic support over time (Focht et al. 2004).

This does not necessarily mean that the rehabilitant must actively participate in all decisions. Person-centered care can also mean accepting that the rehabilitant is prepared to relinquish responsibility and decision-making authority.

In this context, certain requirements are placed on the rehabilitant with regard to shaping the therapy process. In order to express preferences and participate in treatment decisions, the rehabilitant must have certain cognitive and social-communicative skills. A certain inequality in the relationship remains insofar as these skills cannot necessarily be expected of the rehabilitant (in contrast to the members of the therapeutic team, for whom such skills are professionally assumed). For example, there are rehabilitants who are unable to actively participate in the decision-making process due to cognitive or psychological limitations, or who deliberately do not wish to display such behavior (Farin 2014).

A4: Interdisciplinary Cooperation

(A4) *Promoting physical activity is an overarching rehabilitation goal that should be addressed through interprofessional collaboration. Movement-based therapists should regularly consult with the various professional groups involved in medical rehabilitation with regard to movement-related rehabilitation goals and appropriate therapeutic approaches.*

Background

In medical rehabilitation, different professional groups work together in an interprofessional team. This generally requires complex organizational and coordinative effort (Deutsche Rentenversicherung Bund 2023). Interprofessional collaboration enables the implementation of a comprehensive biopsychosocial treatment approach and can both improve treatment outcomes for the rehabilitants and increase team performance and satisfaction in the therapeutic team (Körner 2022; Worringen et al. 2024).

Physical activity promotion is an objective that should be addressed on an interprofessional basis and for which the various benefits from different areas within the KTL are described (Deutsche Rentenversicherung Bund 2014). In particular, services from KTL chapters A (sport therapy), C (information, motivation, training) and L (recreational therapy) are aimed directly at changing physical activity behavior. Physiotherapy services (KTL Chapter B) can play a supporting role in guiding patients towards a physically active lifestyle, insofar as they aim to improve or restore physical functions and reduce pain. In the case of psychological comorbidity and problems in coping with disease, services from KTL chapters F (clinical psychology, neuropsychology) and G (psychotherapy) can also support a sustainable health-oriented change in behavior. In addition, individual services from KTL chapters E (occupational, occupational and other functional therapy), H (rehabilitation care and education) and D (clinical social work, social therapy) can support the introduction to self-reliant movement.

Actors from various healthcare professions are involved in the provision of therapeutic services. In addition to people from sport therapy and physiotherapy, psychologists, doctors and, where appropriate, people from other qualified professional groups (e.g. social work or social education) work together towards the goal of promoting physical activity. While some services can only be provided by a narrowly defined professional group (e.g. medical advice only by doctors or sport therapy only by qualified exercise specialists), other services can be provided by all qualified professional groups (e.g. services for information, motivation, training). In addition, the content of individual KTL services may overlap; for example, movement-related learning content is addressed in both KTL chapter C and chapter A. This particular constellation in the provision of services relating to physical activity promotion leads to a high demand for coordination between the professional groups working in rehabilitation.

Movement-based therapists play a central role in promoting physical activity. Only they can provide the physical activity-promoting therapeutic services listed in KTL chapters A, B, and L. They are often able to simultaneously provide appropriate services from chapter C (information, motivation, training) on the topic of movement. Movement-based therapists should consult with other rehabilitation professionals on the following aspects of promoting physical activity:

- Speaking a common language when it comes to movement, i.e., conveying a coordinated, shared message.

- Using all diagnostic information relevant to promoting physical activity from the various areas of therapy (see recommendation C1).

- Coordinating general rehabilitation goals with the specific movement-related goals of the patients undergoing rehabilitation (see recommendation C2).

- Coordinate the physical activity-promoting content provided by sport therapy and physiotherapy with the therapeutic content provided by other professions (in particular, coordinating the learning content from KTL Chapter A (Sport Therapy) with that from Chapter C (Information, Motivation, Training)).

- Coordination of the use of behavior modification techniques that are established in both movement-based therapy and psychological interventions (e.g., the development of action plans and coping strategies (see recommendation C8).

- Therapeutic approach for promoting physical activity in specific problem circumstances, such as psychological and other comorbidities (e.g., depression, fatigue, chronic pain).

Formats and venues that can be used for interprofessional coordination can be found in the current DRV guide “Professional groups in medical rehabilitation and their interprofessional cooperation.” These include, among others:

- Interdisciplinary team meetings,

- Further education and training, or

- Development and coordination of treatment concepts for individual specialist departments and the overarching training and therapy concept.

Practical Recommendations Part B: Didactic-methodical implementation

B1: Linking training and practice, learning and experience

(B1) *Movement-based therapy aimed at enabling rehabilitation patients to engage in self-determined, health-enhancing physical activity is based on the principle of combining practice and training, learning and experience. This approach accounts for perspectives from exercise science, movement science, medicine, education, psychology, and social ecology.*

Background

Enabling rehabilitation patients to engage in competent, health-oriented physical and sporting activities does not happen incidentally during physical training and exercise - it requires a targeted intervention approach. (Huber 2012) The intervention model for promoting physical-activity-related health competence (PAHCO) (Pfeifer et al. 2013; Pfeifer und Sudeck 2020; Sudeck et al. 2020) describes the link between the content and methods of training and practice with elements of learning (i.e., cognitive and motor) and personal experience (i.e., cognitive and affective-emotional). This link is considered a fundamental prerequisite for competence development (see Fig. 2).

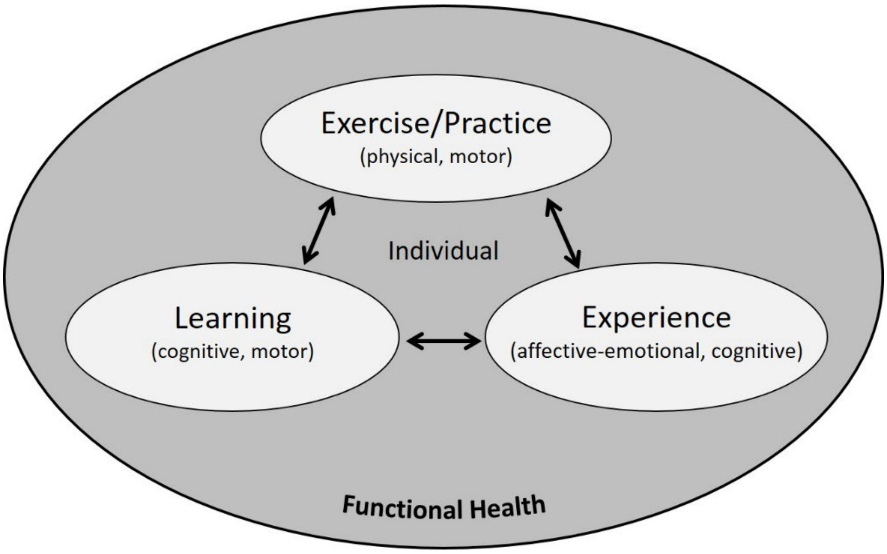

Figure 2: Intervention model for promoting physical-activity-related health competence (adapted from Pfeifer et al. 2013)

For movement-based therapy practice, this means combining and arranging content and methods at all three intervention levels (i.e., practice and training, learning, experiencing and discovering) didactically and methodologically such that the successful development of PAHCO is realized.

This means, for example, that

a) exercise forms for independent functional gymnastics and the methodology of training control (i.e., intensity, repetitions, series) of submaximal strength training are learned,

b) this training is then carried out using self-observation via written documentation of performed repetitions and perceived exertion, and

c) individuals or groups are encouraged to reflect on their training experiences, successes, and/or how they dealt with any difficulties.

If such an approach succeeds in promoting PAHCO, this will lead to an improvement in individuals' ability to cope with health problems and strengthen their biopsychosocial health status.

The target areas listed in Table 1 characterize the three areas of practice and training, learning, and experience and discovery. They illustrate the multidimensional nature of resource-oriented exercise and movement-based therapy (Schüle und Huber 2012) and underscore the ways in which PAHCO is linked. (Pfeifer und Sudeck 2022).

Part C of the practical recommendations provides further details on content and methods that describe implementations and connections between the three areas.

Table 1: Overarching target areas and specific goals for practice and training, learning, experiencing in movement therapy

**Area**	**Ziele**
Practice and training.<br>(Psycho-)physical and motor skills; strengthening physical resources and body awareness and movement perception; developing movement competence.	- Generation of structural and physiological adaptations (e.g., musculoskeletal, sensorimotor, cardiopulmonary, hematological, metabolic, endocrinological, immunological) to adequate suprathreshold stresses on the organ system through training; improvement of motor skills<br>- Improvement of coordination and sensorimotor control for everyday and health-related movement tasks/requirements; initiation of the teaching of motor skills as a basis for independent movement<br>- Improvement of body and movement perception
Learning.<br>Motor, cognitive, and motivational-volitional target area; development of motor skills, control skills, and self-regulation skills	- Teaching motor skills for independent health-enhancing physical/sporting activities (e.g., movement techniques for Nordic walking, cycling, or swimming, exercises for independent fitness training)<br>- Communicating knowledge about the effects of movement (known as “effect knowledge”) and about (movement-related) ways of coping with health problems<br>- Communicating knowledge about the implementation (i.e., planning, implementation, control) of physical/sports activities or training (known as “action knowledge”)<br>- Communicating knowledge and behavioral change techniques to regularly incorporate exercise into one's own daily routines at work and during leisure time (e.g., planning techniques, training documentation, and self-observation for exercise-specific self-regulation)
Experiencing.<br>Affective-emotional, cognitive, and motivational-	- Facilitating positive experiences of movement and the joy of movement

volitional objectives; development of control skills and self-regulation skills.	- Facilitating movement- and exercise-related experiences of self-efficacy<br>- Building motivation and volition for health-enhancing physical activity (e.g., internalizing motivations for regular sports activities)

Successful implementation in the three target areas (i.e., practice and training, learning, and experiencing; see Fig. 2) requires different perspectives and the use of methods and theories from different fields of knowledge. Content from training science, movement science, and medicine is fundamental to the safe and effective use of exercise and training forms. In addition, movement-based therapy that promotes physical activity also integrates educational, psychological, and socio-ecological perspectives.

Movement-based therapy from various perspectives:

Training and exercise science perspective:
- Focus on optimizing body functions and structures
- Use of methods and content to teach and improve motor skills
- Use of training science concepts to develop optimal exercise doses and training plans
- Use of exercise science knowledge to enable optimal motor learning

Medical perspective:
- Focus on physical health and the medical aspects of movement-based therapy
- Use of medical concepts and findings (especially from sports medicine) to achieve biomedical health goals and ensure the safe use of movement for people with non-communicable diseases

Pedagogical and psychological perspective:
- Focus on educational content relevant to independent physical activity and the associated teaching/learning methods, as well as on ways of influencing the motivational and volitional factors that influence a physically active lifestyle
- Use of pedagogical and psychological concepts to account for individual behavioral changes, motivation, and emotional aspects connected to movement-based therapy
- Consideration of the mental health effects and increases in physical and mental well-being that can be achieved through physical activity
- Examination of the interaction between physical and psychological aspects and how they influence each other (e.g., affective-emotional assessments of different levels of stress)

Social-ecological perspective:
- Focus on the environmental factors that influence physical activity
- Consideration of the site-specific conditions at the rehabilitation patients' places of residence
- Consideration of relevant social environmental factors (e.g., relationships within the family, with friends, or at work) in terms of their beneficial and detrimental influences on a physically active lifestyle

B2: Importance of prior movement experiences

(B2) *Movement-based therapy aimed at promoting physical activity should consider the previous positive and negative movement experiences of rehabilitation clients. This includes not only their motor abilities, but also their cognitive evaluations and emotional-affective experiences related to physical activity.*

Background

Rehabilitation patients each have a unique movement history, as they have usually already participated in sporting activities in various areas (e.g., school sports, club sports, health sports, informal self-organized activities) in the course of their lifetime and have acquired a diverse pool of experience.

These experiences form the basis for physical and motor characteristics (e.g., motor skills, coordination), which in turn are important prerequisites for future physical and sporting activities. However, current sports psychology research increasingly shows that the affective and emotional evaluation of previous movement experiences is still not sufficiently considered in targeted physical activity promotion. Historically, primary focus has often been placed on cognitive-rational decision-making processes to positively influence physical activity behavior (Rhodes et al. 2019), e.g., on the potential health benefits of physical activity. However, reviews show that such measures tend to have small and short-lived effects on physical activity behavior (McEwan et al. 2016; Howlett et al. 2018). Convincing physically inactive people utilizing rational argumentation seems ineffective. The affective experience of the activity (e.g., discomfort due to physical exertion or motor overload or underload) or the social context (e.g., shame due to unsuccessful movement execution) should therefore be addressed much stronger and more systematically to successfully promote physical activity (Rhodes und Kates 2015; Ekkekakis et al. 2024).

The role of affective experience and the anchoring of movement experiences in emotional memory is often discussed against the backdrop of “hedonistic theories” (Ekkekakis et al. 2021; Ekkekakis et al. 2024). The basic principle of hedonism states that people tend to avoid activities associated with unpleasant experiences and are more likely to (re)engage in activities associated with pleasant, positive experiences. Thus, attempting to convince an overweight person to exercise primarily utilizing an argument related to weight loss may fall short if previous experiences with exercise were highly unpleasant (Thiel et al. 2020).

Future physical activity behavior also finds relevancy in the potential connectivity between prior movement experiences and automatic affective evaluations of physical activity options (e.g., a call for more activity or a specific physical activity option) (Brand und Ekkekakis 2018). Such automatic affective processes can compete directly with cognitive-rational considerations (e.g., the assessment of positive health benefits). Thus, previous movement experiences and their affective evaluation can give rise to very different motivational tendencies, ranging from strong rejection to strong acceptance of sports activities for an individual (Ekkekakis et al. 2021).

With the help of a current model of affective movement experiences (known as “affective exercise experiences” or AFFEXX) (ebd.), relevant areas of experience can be illustrated. First, core affective experiences with movement are described, i.e., states of being that are fundamentally associated with movement:

- comfortable/positive vs. uncomfortable/negative state of being;

- energetic vs. energy-lacking state of being;
- calm/relaxed vs. restless/tense state of being.

In addition, affective movement experiences can be linked to individual assessments, which can be described in terms of opposing experiences or evaluations for sport and exercise:

- enjoying vs. disliking group sports
- remaining shy vs. wanting to be seen in a group
- fearing physical injury vs. valuing physical resilience gained through movement
- feeling competent vs. feeling incompetent in sports activities
- finding physical activity boring vs. finding it interesting
- feeling ashamed of one's own inactivity vs. being proud of one's physical activity behavior

The AFFEXX model is intended to illustrate how the positive and negative experiences of movement mentioned in the practical recommendations can be formulated more specifically. They emphasize the importance of affective-emotional experiences with movement, which can be linked to cognitive evaluations. Movement-based therapy that promotes physical activity therefore requires therapists to be sensitive to different experiences and, in principle, to be able to accept aversions to exercise or see them as a starting point for therapeutic action.

A practical consequence can thus be derived: it is important not to place all focus on rational, reflexive processes (in the sense of cognitive persuasion), but also to address both positive and negative previous experiences with movement. Positive experiences with movement can be used as a basis for designing therapy or exercise guidance and emphasis can be placed on facilitating positive exercise experiences.

From a methodological perspective, this requires an experience-centered approach that enables structured practical movement experiences, cultivates reflection on these movement experiences, and reflects on them with potential learning objectives in mind (Kolb 2015; Schön 2017). The categories of affective movement experiences described in the AFFEXX model can provide relevant points of reference for an experience-centered approach in movement-based therapy. Consequently, positive movement experiences and movement enjoyment can be specifically targeted as important starting points for an increasingly intrinsic basis for motivation.

Further information on implementation can be found in Part C of the practical recommendations for specific therapy design (see C4, C6, C7). In addition, interprofessional exchange (e.g., with psychologists) may be useful if strongly negative experiences in sport and exercise settings cannot be addressed in exercise and movement-based therapy alone (see also recommendation A4).

Practical Recommendations Part C: Concrete Therapy Design

C1: Assessment

(C1) *Movement-based therapy should be planned and implemented based on assessment. Assessment can also be used to monitor the course and outcomes of therapy, ideally including follow-up care. To promote physical activity, assessment should particularly consider clients´ previous physical activity behavior as well as key physical, psychological, social, and environmental factors that influence regular activity.*

Background

Assessment involves systematic recording and evaluation of individual aspects of the functional abilities and disabilities of a rehabilitation patient. An initial assessment is carried out at the start of rehabilitation. This forms the basis for further therapy steps, in particular planning and managing the content of movement-based therapy. Repeated assessments during and after rehabilitation help to adapt interventions and analyze the results of therapy.

Assessment should be carried out using quality-assured test procedures wherever possible. Quality-assured test procedures meet certain quality criteria. The main scientific quality criteria are objectivity, reliability, and validity. Objectivity means that the test result is independent of the examiner. Reliability means that the test measures accurately and reliably. Validity means that the test measures what it is intended to measure. In addition to scientific quality criteria, quick and easy implementation (test economy) of the test procedures is similarly important for use in everyday rehabilitation. Similar to a screening, shorter test procedures can provide valuable initial information about the rehabilitation patients. Data collected using quantitative test procedures (e.g., physical function tests or questionnaires) can be supplemented by qualitative procedures (e.g., initial interviews). This also allows for more space for rehabilitation patients' personal perspectives and self-assessments.

Target group: All rehabilitants.

Contents and methods for assessment

Movement-based therapy assessment usually follows an initial medical examination. This is a prerequisite for the safe use of movement as part of movement-based therapy. An initial medical examination provides information on the appropriate amount of exercise for each individual (e.g., suitable intensity, frequency, regularity) and identifies risk factors and contraindications for specific forms of physical activity.

Physical activity behavior: Assessing preexisting physical activity behavior is the starting point for targeted measures to promote physical activity and for setting individual goals (see recommendation C2). Various tools are available for assessing physical activity behavior (e.g., questionnaires or pedometers).

To evaluate a person's activity level, two short questions can serve as a basis for assessment: (a) "On average, how many days per week do you engage in moderate or vigorous physical activity, such as brisk walking?" and (b) "On average, how many minutes do you spend on this activity on those days?" (Lobelo et al. 2018). This screening to determine compliance with physical activity recommendations

is known internationally as Physical Activity as a Vital Sign (see Table 2). Further measurement tools for physical activity can be found in Table 2.

In general, questionnaires on physical activity behavior provide valuable details on various activities, such as sports, leisure, transportation, work, or housework and gardening. However, questionnaires often carry the risk of "overreporting," meaning that people overestimate their physical activity. Device-based measurement methods (pedometer, accelerometer, etc.) provide more accurate values on the extent and intensity of physical activity, but less information on the context in which the activities took place. For inpatient rehabilitation, device-based measurement of current physical activity in the individual's natural environment is difficult, making the use of questionnaires preferential.

In addition to physical activity behavior, assessment should take into account select physical, psychological, social, and environmental factors that influence regular physical activity. In line with the International Classification of Functioning, Disability, and Health (ICF), the assessment for targeted physical activity promotion therefore refers to body functions and structures, activities, and participation, as well as personal and environmental context factors.

Bodily aspects: Measuring physical function and performance is common in movement-based therapy. As a rule, the more active a person is, the better their physical fitness. In addition to physical activity behavior, directly testing aspects of bodily function and performance can be helpful in planning exercise and training in movement-based therapy. Depending on the disease and individual medical condition, this can be linked to cardiorespiratory, morphological, metabolic, and muscular components (Pelliccia et al. 2021). Motor tests are commonly used to assess physical function and performance, such as the 6-minute walk test, manual muscle function testing, or the one-repetition maximum. Instructions, standards, and references for the physical test procedures recommended for individual indications can be found, for example, in the "Guidelines for Exercise Testing and Prescription" of the American College of Sports Medicine (ACSM) (Riebe et al. 2018) or in the Handbook of Motor Tests (Bös 2017).

Psychological aspects: Various psychological factors influence physical activity behavior. International concepts for promoting physical activity in exercise therapy (Elvén et al. 2015; Lein et al. 2017) recommend assessing the following modifiable aspects related to physical activity: readiness to change, attitudes, expectations, perceived barriers, and self-efficacy. Movement-related goals, motives, and preferences, the degree of intrinsic motivation, and skills for implementing health-enhancing exercise are similarly important. Table 2 provides examples and suggestions for suitable assessment tools.

Environmental factors: Physical activity behavior is also influenced by environmental factors (Bauman et al. 2012). Environmental conditions can either enable and encourage physical activity or hinder and discourage it (Michie et al. 2011). Social environment is important for physical activity, fostering others' encouragement, prompts, reminders, assistance, and the direct availability of exercise partners (Fuchs 1997), while the structural or physical environment is similarly influential, e.g., the accessibility of places suitable for physical activity such as swimming pools or parks. In order to identify the environmental factors that influence a person's physical activity, they can be asked after their perceptions and assessments of their environment in terms of physical activity.

To date, there is no generally accepted standard for behavior-oriented, biopsychosocial assessment for movement-based therapy. However, research has identified a number of validated instruments and questionnaires that have been proven effective. The following table lists measurement instruments that can be used to record relevant behavioral determinants of physical activity. Reference is also made to the questionnaire for assessing PAHCO (Carl et al. 2020; Sudeck et al. 2023), which can be used to assess the prerequisites for independent health-enhancing physical activity. In

addition, the table contains references to practical recommendations C2-C9, which describe specific content and methods for targeting the possible aspects of an assessment.[4]

Tab. 2: Examples for Assessment Instruments

Features	Potential measurement instruments	Associated practical recommendations
**Section: Physical activity behavior**		
Current physical activity behavior (Questionnaire)	- Physical activity as vital sign (Short screening to reach movement recommendations) (Golightly et al. 2017)<br>- BSA questionnaire (detailed measurement of physical activity with information on sports, leisure activities, transportation, occupation, housework, garden work, sitting time; Fuchs et al. 2015)<br>- German-PAQ-50+ (Detailed measurement of physical activity with information on sports, leisure activities, transportation, occupation, housework, garden work, and free time; Huy 2011).	Is-Analysis of current movement behavior as a general basis for promoting physical activity C2
Current physical activity behavior (device-supported)	- Pedometer<br>- Accelerometer (e.g. Actigraph)<br>- Smartphone-based Apps (e.g. Google fit, Pacer, Fitbit, Accupedo, Activitytracker)	
**Section: Physical aspects (cross-indication)**		
Motor function status and movement-related skills	*Examples of motor function tests*<br>- 6-Minute walking test (Bösch and Criée 2020)<br>- PWC endurance test (Rost and Hollmann 1982)<br>- Timed Up and Go Test (Freund 2017)<br>- Berg-Balance-Skala (Berg 1989)<br>- Stand-and-Reach Test (Bös 2017)<br>- Manual muscle function test (Smolenski et al. 2020)	C3, C4
	Questionnaires<br>- Questionnaire to assess physical-activity-related health competence (PAHCO; movement competence area, Carl et al. 2021)<br>- Questionnaire to assess motor function status<br>- (FFB-Mot, Bös et al. 2002)<br>- Questionnaire to assess physical functioning in everyday life (e.g., subscale from IRES-24, Wirtz et al. 2005)	

[4] It should be noted here that the listed assessment methods originate primarily from research contexts and are therefore usually very comprehensive and multifaceted. A multi-layered survey of the listed influencing factors is therefore usually time-consuming and only of limited practical use for routine application in everyday rehabilitation. Until a manageable assessment battery is available, practitioners in rehabilitation are recommended to use selected assessment instruments from Table 2.

Section: Psychological aspects		
Motivation and Volition	- Questionnaire to assess willingness to change (FEVER; Hasler et al. 2003)<br>- 1-item assessment of intention to engage in physical activity (Göhner et al. 2009)<br>- Affective and cognitive attitudes toward physical activity (Brand 2006)<br>- Berner Motive and Goal Inventory for Leisure and Health Exercise (BMZI; Lehnert et al. 2011)<br>- Intrinsic motivation for physical activity (Seelig and Fuchs 2006)	C2, C4, C6, C7,
Movement-related self-regulation skills	- Questionnaire to assess physical-activity-related health competence (PAHCO; self-regulation skills) (Sudeck and Pfeifer 2016)<br>- Questionnaire on motivational skills for movement and exercise (Schorno et al. 2021)	C7, C8
Barriers	- Exercise-related situational barriers (Krämer and Fuchs 2009)	C8
Body perception	- FBeK questionnaire for assessing one's own body (Brähler et al. 2000)<br>- Scale for body perception from the questionnaire on physical-activity-related health competence (Carl et al. 2021)	C4
Movement-related self-efficacy	- Questionnaire scale for task-specific self-efficacy (scale from PAHCO questionnaire, ebd.)<br>- Questionnaire on stage-specific self-efficacy (Göhner et al. 2009)	C4<br><br>C8
Movement-related control competency	- Questionnaire to assess physical-activity-related health competence (PAHCO ; area of control competence) (Sudeck and Pfeifer 2016)	C5
Affective core experiences with exercise	- Questionnaire on affective movement experiences (AFFEXX questionnaire); (Ekkekakis et al. 2021; Brand et al. 2023)	C6, C5
**Sector: Environment**		
Conditions in the (living) environment that promote or hinder physical activity	- „Assessing Levels of Physical Activity" (ALPHA; Paulsen et al. 2022)	C2, C8, C9
Conditions in the social environment that promote or hinder physical activity	- Exercise-related social support (Family, friends and acquaintances; Fuchs 1997)	C2, C8, C9

C2: Physical activity-related therapy goals

(C2) *Physical activity-related therapy goals should be developed collaboratively and through dialogue with rehabilitation clients. The degree to which these goals are achieved should be reviewed and reflected upon together with the clients.*

Background

Setting individual rehabilitation goals is considered an important basis for managing the rehabilitation process in a manner that is as needs-based and goal-oriented as possible (Dibbelt und Greitemann 2011; Glattacker et al. 2015). Individual rehabilitation goals relate in particular to the period during and after medical rehabilitation, such that the respective goals can be further pursued (see www.reha-ziele.de; Glattacker et al. 2015) (see also recommendation A4).

It is important to differentiate and define movement-related therapy goals in order to establish motivation for participation in movement-based therapy and to strengthen motivation for continuing physical activity after the rehabilitation phase. Movement-related therapy goals can be formulated as short-term goals for the immediate period of medical rehabilitation or as medium- and long-term goals for the period after rehabilitation.

Rehabilitation patients should be actively involved in setting therapy goals (participatory decision-making). This promotes a person-centered approach and enables greater autonomy, creates more transparency, and can be conducive to the successful pursuit of individual rehabilitation goals (see also recommendations A3 and A4).

A handful of basic principles define the formulation of motivating rehabilitation goals and exercise-related therapy goals (Glattacker et al. 2015):

- Clear and unambiguous. Goals should be clear and unambiguous such that the desired outcome or behavior can be described in concrete terms.
- Aspirational goals over avoidance goals. Aspirational goals (e.g., “I want to maintain my independence in everyday life and leisure activities,” “I want to improve my back muscles”) are considered to have a higher and more sustainable motivational potential than avoidance goals due to their positive orientation.
- Relevant to everyday life. Goals should be relevant to the everyday life of the people undergoing rehabilitation. They should be in line with the individual's own values and preferences and thus be subjectively meaningful and attractive (also referred to as self-determined goals).
- Not too difficult and not too easy. Goals should neither underchallenge nor overchallenge the individual in order, enabling the potential to motivate the person undergoing rehabilitation while also being realistically achievable.
- Relevancy to the present and the self. To increase commitment, it is recommended that goals be formulated in the first person and in the present tense, that they be written down, and that they be discussed with others (e.g., other rehabilitation patients or therapists) (Rauen 2021).

In accordance with the recommendations in the Reha-Ziele (Rehabilitation Goals) workbook (Glattacker et al. 2015), it is important to define the time frame for the goals (short, medium, or long term). In addition, individual goals can be differentiated according to whether they are formulated as movement-related health goals or behavioral goals. For those who have been historically relatively inactive, individual learning goals that relate to movement-related knowledge, skills, processes, and

strategies that need to be developed as prerequisites for independent movement can be particularly important at first (see Swann et al. 2021). Learning goals are distinguished from performance goals, which relate to specific behaviors or health consequences (see Table 3). Such individual learning goals can be particularly relevant as short-term goals for enabling health-enhancing physical activity (Strohacker et al. 2024).

Table 3: Examples of goal formulations for different types of goals

	Short-term<br>(e.g. during rehabilitation)	Medium- and long-term<br>(after rehabilitation)
health goals	I want to be able to breathe better.	I want to improve my quality of life and be physically active to achieve this.
	I want to have fewer health issues.<br>*(avoidance goal)*	I want to be more resilient at work.
	I want to sleep better and be able to concentrate better during the day.	
	I want to strengthen my muscles, especially in my back and stomach area.	
	I want to improve my physical endurance.	
behavior goals		I would like to regularly do a combination of endurance activities and muscle strengthening (twice a week).
		I want to be able to practice my sport again without any discomfort.
		I want to get into the habit of walking regularly.
		I would like to ride my bike to work.
learning goals	I want to find a good way to deal with physical stress and relaxation.	
	I want to feel safer when I exercise.	
	I would like to learn about ways in which I can exercise effectively despite my health limitations.	
	I would like to find a physical activity that suits me, that I enjoy, and that I can imagine doing in the long term.	

The formulation of specific goals has proven successful in bringing about lasting changes in physical activity behavior (Garstang et al. 2024). However, the pursuit of individual goals does not end with the completion of medical rehabilitation, as the examples in Table 1 clearly show. Therefore, the involvement of rehabilitation patients should also aim to enable them to set their own health and behavioral goals after rehabilitation (if necessary) and to use these goals to regulate their own physical activity - something particularly important in the event of relapses into a severe lack of physical activity or phases of highly fluctuating physical activity patterns.(see recommendation A2: Development of movement-specific self-regulation skills; see recommendation C8 Volition).

Target group: Rehabilitation patients who are not regularly active and who intend to start or continue regular physical activity; rehabilitation patients who need to adapt their physical activity due to their health circumstances.

Contents and methods for developing and formulating goals

A number of working materials - already extensively used in research and rehabilitation practice - are now available for developing and formulating movement-related therapy goals. This includes the

methods and application examples from the Reha-Ziele (Rehabilitation Goals) workbook (see www.reha-ziele.de; Glattacker et al. 2015), which can provide guidance, as well as a number of manualized procedures for movement therapy (see Table 4).

The procedures provide a structure for formulating goals. As a rule, criteria are specified that can be used to optimize the formulation of goals in line with the basic principles outlined above. A well-known model is the S.M.A.R.T. goal set (Bovend'Eerdt et al. 2009; Dibbelt und Greitemann 2011), which has the following characteristics:

- **S**pecific: Is there a concrete description of the target state or desired behavior such that achievement goals are clear?
- **M**easurable: Is it possible to verify the extent to which the goal has been achieved?
- **A**ttractive: Is the goal worthwhile from the perspective of the treating professionals and those being treated? Is the goal considered relevant by the people undergoing rehabilitation due to its importance in their everyday life, for example?
- **R**ealistic: Is the goal achievable from the perspective of the treating professionals and those undergoing rehabilitation?
- **T**erminable: What is the time frame for the goal and when should it be achieved (e.g., in the short term at the end of rehabilitation or in the long term within the first six months after rehabilitation)?

In all application examples from the field of movement-based therapy (see Table 4), written documentation is used to support the formulation of movement-related therapy goals. These are often combined with elements of goal review or further steps in treatment and coping planning (see Recommendation C8). The organizational forms vary, with individual goal setting appearing feasible in groups of up to approximately 12 people; combinations with individual counseling are also planned. In some cases, the programs are supported by digital applications for goal setting, documentation, and review.

Table 4: Program examples from practice of the concrete implementation of content and methods with regard to goal setting and goal review

**Program example**	**Elements of goal setting and goal review, as well as other content and methods related to other practical recommendations**	**Organizational structure**	**Sources:**
MoVo-LiSA	- Formulation of health goals (associated with movement)<br>- Formulation of behavioral goals<br>- Review of the following criteria: appropriate, practical, precise, and effective (3pw check)<br>- Treatment and coping plan (see recommendation C8)	- Combination of group sessions, individual consultations, and follow-up contact by telephone or in writing	Göhner & Fuchs (2007) https://www.movo-konzept.de/
Movement and exercise in everyday life (VIN-CET, INOP)	- Formulation of behavioral goals<br>- Treatment and coping plans (see recommendation C8)<br>- Observation of the achievement of behavioral goals for the period following rehabilitation	- Integration into (closed) group therapy (up to a maximum of 12 people)<br>- Connection with exit interviews (INOP)	reha-ziele.de, Sudeck (2006)

ImPuls - strong psyche through movement and motivation	- Individual definition of S.M.A.R.T. goals for health and symptoms (with potential association to movement)<br>- Review and reflection on goal achievement (NRS)	- Closed groups of 6-8 people, follow-up contact by telephone	Wolf et al. (2020b)
Coach-to-Move	- Review and reflection on goal achievement (NRS)<br>- Individual definition of S.M.A.R.T. goals in relation to physical activity (use of Motivational Interviewing and consideration of Shared Ddecision Making)<br>- Collaborative barrier management (see recommendation C8)	- Closed groups of 6-8 people, follow-up contact by telephone<br>- Optimization of regular physiotherapy treatments (individual sessions)<br>- First treatment approx. 90 minutes, followed by 30-minute sessions	de Vries et al. (2015)
Curriculum Generic self-management modules (SelMA)	- Clarify personal goals<br>- Plan steps to achieve goals<br>- Anticipate difficulties in achieving goals<br>- Check progress toward goals and adjust actions and goals as necessary	- Closed group (maximum 15 people)<br>- Three modules, each lasting 60 minutes	Deutsche Rentenversicherung Bund (2021)

Contents and methods for reviewing and reflecting on goals

For short-term therapy goals, goal achievement can be reviewed during rehabilitation. Reviewing longer-term rehabilitation goals is desirable, but is not yet a widely established standard in rehabilitation practice.

Reviewing the extent to which goals have been achieved enables rehabilitation patients and the rehabilitation team to share their experiences of movement-based therapy, providing a direct opportunity for feedback on experiences during the therapy process. This can also lead to individual movement-related goals being adjusted and specified in more detail for the period following rehabilitation.

Depending on the objective, various methods can be used to monitor therapy and review goals (see recommendation C1). Objective assessment and measurement methods can be used, for example, to assess physical functions or motor skills and abilities. In addition, standardized procedures can be used to record rehabilitation patients' individual perceptions on the degree to which they have achieved their goals (see www.reha-ziele.de; Glattacker et al. 2015). Examples include:

- Numerical rating scales (NRS): Determination of the perceived degree of target achievement by means of queries on a scale from 0 to 100 or from 0 to 10.
- Goal Attainment Scaling (Zwingmann, 2003): Assessment of individual goal progress based on a scale ranging from -2 (much less than expected), -1 (less than expected), 0 (expected result), +1 (more than expected) to +2 (much more than expected).

Guidelines for person-oriented communication and dialogue-oriented goal setting

The principle of person-oriented care is of great importance for setting goals. Appreciative and active communication and an active and participatory role for those undergoing rehabilitation are conducive to successful goal setting (see Recommendation A3).

Discussions in groups and one-on-one situations are designed to help participants agree on common goals ("agree") rather than simply offering recommendations from an expert perspective ("advise"). Rethorn et al. (2022). refer to this as a shift in roles from expert to coach. In this role, therapists support rehabilitation patients in formulating and setting their own goals, offering information, advice, and solutions as needed. In this context, basic attitudes and techniques for motivational interviewing are important (Miller 2014), including techniques for asking open questions, active listening, and offering information and advice (Messner 2018) - all of which are applied on the basis of an empathetic therapeutic attitude and the acceptance of the participant, as well as the elicitation of motivators and resources. In contrast, there are less suitable techniques; patronizing communication, paternalistic attitude, controlling language ("You must..., you should..." etc.) accompanying clear expert instructions for goal setting (Rethorn et al. 2022).

C3: Improve physical and motor prerequisites

(C3) *In movement-based therapy, the physical and motor prerequisites for health-enhancing physical activity should be optimized.*

Background

Improving motor skills and abilities is a key objective of exercise therapy in medical rehabilitation. Exercise and training methods designed to improve motor skills and abilities are traditionally core components or constituent elements of movement-based therapy and its various applications in sport therapy and physical therapy (as well as in related fields such as recreational therapy and, to some extent, ergotherapy). Accordingly, a variety of exercise forms and training methods belong to the basic repertoire of movement-based therapists. The design of exercise and training processes in medical rehabilitation initially follows the principles known from training science (e.g. Hohmann et al. 2020) and movement science (e.g. Hossner und Künzell 2022). However, as is customary in the practice of movement-based therapy, the respective individual's previous experience and health restrictions must be taken into account (e.g. Radlinger et al. 1998; Schüle and Huber 2012; Froböse et al. 2022).

Specific forms of exercise and training are designed to induce structural and physiological adaptations (musculoskeletal, sensorimotor, cardiopulmonary, hematological, metabolic, endocrinological, immunological, etc.) (see recommendation B1). The aim is to improve or, depending on the individual's starting point, optimize the body structures and functions relevant to human motor skills and independent physical activity (as key determinants of motor abilities such as endurance, strength, speed, agility, and coordination). These are an important prerequisite for the ability to cope with the immediate motor demands of everyday physical activities (e.g., climbing stairs, cycling) and sporting activities (e.g., Nordic walking, jogging, muscle-strengthening exercises, ball games) (see recommendation A2 Movement competence).

Movement-based therapy in medical rehabilitation involves using exercises and training methods that can achieve initial adaptation effects within a limited period of just a few weeks. This includes, for example, initial endurance training effects such as improving cardiovascular function, improving intra- and intermuscular coordination during strength exercises, or improving sensorimotor control to stabilize the knee or hip joints or the spine during coordination tasks (e.g., climbing stairs or lifting a load).

In addition, exercises from the field of motor learning serve to teach and improve motor skills, thereby expanding the range of possibilities for independent physical activity (see recommendation B1). Lastly, the program aims to maintain and further strengthen the effects of the exercises and training achieved during the rehabilitation stay. This includes, for example, optimizing movement technique in (Nordic) walking and running, learning or improving swimming techniques, learning or relearning cycling, or even teaching techniques from the field of recreational sports (e.g., table tennis or badminton).

In the model of physical-activity-related health competence (PAHCO), adequate body and movement awareness is also understood as a component of *physical and motor prerequisites for health-enhancing physical activity* (see recommendation A2, movement competence). Body and movement awareness is considered important for competently executing motor tasks and tolerating and controlling the demands of physical activity, assuming that people with good awareness of conditions and changes in, for example, muscle tension and joint positions during postures and movement sequences (e.g. range

and speed of movement or use of force in various movements) also have adequate prerequisites for mastering varying motor requirements (e.g., in balance tasks) and for learning forms of movement relevant to everyday motor skills or health-related physical activity. The corresponding ability to perceive the body and movement (and also to consciously reflect on this) helps to reduce uncertainty when moving and to strengthen confidence in one's own movement capabilities (task- and behavior-related self-efficacy; see recommendation C4). In addition, it is assumed that people with good body awareness are more likely to be able to appropriately regulate the intensity of exercise and physical training and thus promote well-being (control competence, see recommendations A2 and C5).

Target group: Individuals undergoing rehabilitation who are affected by physical deconditioning due to an inactive lifestyle or specific disease-related limitations, or who have low physical fitness.

Contents and methods for improving physical and motor skills:

To improve physical and motor skills, exercises and training methods can be selected from the wide range available in the field of movement-based therapy, based on the individual's starting point and goals. Specific guidelines can be found in the relevant sources on exercise and movement-based therapy or on training in therapy, for example in the guidelines on physical activity for the treatment of cardiovascular diseases (Bjarnason-Wehrens et al. 2009). A detailed list and explanation of these sources is beyond the scope of these practical recommendations. Regarding the goal of promoting physical activity, these practical recommendations focus on how the specific training and exercise methods known to exercise professionals can be embedded in an overall movement-based therapy concept.

C4: Strengthen confidence

(C4) *Movement-based therapy should strengthen confidence in the independent realization of health-enhancing physical activity. This includes the perception of one's own motor abilities and skills, as well as the enhancement of self-efficacy for specific movement tasks and for maintaining regular physical activity behavior .*

Background

People need confidence in their own movement-related abilities and skills needed to cope with the demands of physical activities in everyday life or in (health) exercise. As an example, only when people believe that they can walk up stairs safely and quickly or ride a bike will they actually carry out these activities, utilizing them to strengthen their motor functioning. This confidence is known as task-related self-efficacy. Task-related self-efficacy is expressed as the belief that you can successfully master the movement-related requirements of a particular physical activity in everyday life and/or sport using your own abilities and skills.

Movement-related self-efficacy is closely linked to body and movement awareness skills (Gothe et al. 2022). Movement-related perceptions play an important role in coping with motor demands and tolerating the strain of physical activity. At the same time, however, it is also relevant for the design and orientation of physical activity to promote health and well-being. Body and movement perception therefore influence both movement competence and control competence (Pfeifer und Sudeck 2020). For example, people with good perception of movement sequences (e.g., amplitude, speed, use of force in various movements) are also well equipped to learn health-related activities. People with good body awareness are, for example, more likely to be able to regulate movement intensity appropriately and thus stay within their physical limits. When movements are successfully learned and individually challenging movement tasks are mastered as a result of adequate body awareness, this promotes a high level of self-efficacy.

In addition to task-related self-efficacy, behavioral self-efficacy is also necessary for regular physical activity. This refers to a person's belief that they can engage in regular physical activity - even when conditions are unfavorable.

Strengthening both aspects of movement-related self-efficacy plays an important role in approaches promoting physical activity. Self-efficacy has been shown to be significant for the selection of one's own physical activities and for their regular performance, even after failures or undesirable results (McAuley et al. 1999). (Movement-related) self-efficacy plays an important role in promoting physical activity, as it is directly linked to motivational aspects (Ashford et al. 2010). People with higher movement-related self-efficacy are more likely to be physically active on a regular basis (Dutton et al. 2009; Darker et al. 2010; Higgins et al. 2014).

In the model of physical-activity-related health competence (Pfeifer and Sudeck 2016), all three competence facets (movement, control, and self-regulation) are driven by self-efficacy beliefs. Movement-related self-efficacy strengthens confidence a) in the ability to use one's own motor skills and abilities to cope with physical activities in an adequate and circumstance-appropriate manner (movement competence), b) in independently designing physical activities in such a way that they have a positive effect on health and well-being (control competence) and c) in the consistent pursuit of physical activity despite resistance and obstacles (self-regulation competence).

Target group: Rehabilitation patients with little confidence in their ability to independently implement health-enhancing physical activity. The recommended content and methods can help motivate individuals with low motivation levels and reinforce the confidence of highly motivated individuals who nevertheless engage in irregular physical activity to start regular activity and maintain it long-term.

Content and methods for increasing self-efficacy

Select an *appropriate difficulty level* for the movement program. This means designing physical activities such that participants are not overwhelmed (nor underchallenged) and can successfully master the movement-related requirements.

- Becoming familiar with and learning challenging yet independently achievable movement tasks
- Becoming familiar with and learning independently performable exercises and forms of movement for health-enhancing exercise; e.g., functional gymnastics exercises or skills for endurance training (e.g., swimming).

The exchange of experiences amongst rehabilitation patients must be facilitated, allowing those who have successfully completed a movement-related task with similar health conditions and/or those who have managed to integrate physical activity into their everyday lives to share their experiences.

- Towards the end of their rehabilitation, patients can share their movement-related experiences with new patients in group discussions. This can be implemented in group discussions or as partner work.

Provide feedback on current exercise performance

- Performance-related: Specific, positive feedback on current physical performance (e.g., “You performed the strength training exercise correctly.”)
- in relation to a standard: evaluation of a performance test in comparison to a general health-related standard or in comparison to age- or gender-specific norm values (e.g., “Your current endurance performance is currently at 60% of the average for people of your age with your condition. You can improve this value through regular physical activity.”)
- Development-related (individual reference standard): Repeated measurement of health-related fitness with explicit documentation and reflection on performance changes together with the rehabilitant based on the results of the first measurement, followed by concrete, positive feedback on individual performance progress (e.g., “Compared to your performance in the first week, you can already do significantly more today.”)

Express your conviction to the rehabilitation patients that they already possess relevant skills and/or are capable of acquiring them (e.g., “I am convinced that you are capable of walking for 10 minutes at a time” or “I am convinced that by the end of your rehabilitation you will be capable of...”).

Introduce rehabilitation patients to *alternative forms of exercise adapted to their circumstances*.

- Allowing for rehabilitation patients to have an opportunity to learn about and try out different forms of physical activities.

Inform rehabilitation patients about how activities of daily living can be performed with their specific physical impairment, e.g., climbing stairs with a hip prosthesis.

Content and methods for improving body awareness and movement perception in connection with exercise and movement activities

Offer a *wide variety of health-related movement techniques* (e.g., different ranges of motion and contrasting movements) while directing attention to individual technical features.

- To guide awareness, rehabilitation patients can be given contrasting tasks, such as movements with different amplitudes or forms of contraction.
- Example: Nordic walking: The movement therapist demonstrates or verbally instructs the participants to perform one or more movement patterns in different ways, e.g., “Walk 50 meters with your arms straight, then 50 meters with your arms bent at a sharp angle, and then find your own optimal elbow angle over the next 100 meters.” Further variations: with heavy use of poles vs. light use of poles, long strides vs. short strides, with relaxed shoulders vs. raised shoulders, etc. – Questions for the group during or after the exercises, e.g., “What differences do you feel? What has changed? What is more comfortable?”

Focusing attention on physiological aspects/body reactions

- Improved awareness of bodily reactions during exercise can be achieved, for example, by focusing attention on certain physiological aspects (e.g., stress reactions to under- or overexertion such as sweating, heart rate, body heat, changes in breathing rhythm) or on feelings of tension in muscles, tendons, and connective tissue (during contractions or stretching).
- Rehabilitation patients observe their physical stress reactions, e.g., sweating, heart rate, body heat, changes in breathing rhythm, and reflect on them in relation to the perceived effort. This helps rehabilitation patients evaluate normal bodily reactions in a positive fashion.
- Example: Focusing awareness on muscle and tendon tension during gymnastic stretching exercises or strengthening exercises.

Exercises for muscle tension and relaxation to *promote body awareness.* Exercises for muscle tension and relaxation can be used to improve body awareness (similar to the instructions for progressive muscle relaxation from Jacobson, or verbal perception guidance on body regions (e.g., “body scan” from Kabat-Zinn 2003) or exercises with tactile stimuli (perceiving and naming pressure points, massage with a spiky ball) or proprioceptive stimuli (in balance and coordination tasks).

C5: Provide knowledge and foster control competence

(C5) *Movement-based therapy should enable rehabilitation clients to structure their physical activity in a way that optimizes physical and mental health benefits while minimizing health risks. This also includes understanding how physical activity can be individually used to cope with health problems and discomfort.*

Background

Movement-based therapy addresses a wide range of issues related to improving and maintaining body functions and structures as well as activities and participation (see recommendation A1). Recommendation C5 refers to the goal of enabling people undergoing rehabilitation to tailor their physical activity to their individual health benefits in the best manner possible. People undergoing rehabilitation should be supported in maximizing the individual potential of physical activity in positively addressing health problems and coping with illness. In the context of physical activity-related health competence, people undergoing rehabilitation should be enabled to design health-enhancing physical activity (control competence) in such a way that they can optimize physical and mental health effects and minimize health risks (for the basics of physical activity-related health competence, see Recommendation A2).

For individuals with non-communicable diseases, exercise can be expected to offer an overwhelmingly positive balance between benefits and risks (e.g., adverse events such as injuries or overexertion). Nevertheless, physical activity recommendations for people with chronic diseases emphasize accounting for individual health conditions in determining the "right amount" of physical activity and ensuring that physical activity is safe and low-risk (Pfeifer und Geidl 2017). This applies, for example, when starting or resuming exercise programs, during phases of disease progression, or when experiencing side effects from other forms of treatment (e.g., chemotherapy). (see recommendation A1). Rehabilitation facilities offer a suitable setting for promoting exercise within an interdisciplinary rehabilitation team. Here, individuals can find expert support in approaching exercise in relation to health problems, impairments, and discomfort. Accordingly, most risks associated with exercise can be minimized by tailoring the level of exertion and the type of physical activity to the individual's illness and symptoms (ebd.).

The practical implementation of recommendation C5 builds on methods for imparting and applying knowledge (Kok et al. 2016). Specific areas for developing control competence (see recommendation A2) are then added in table form. To this end, content and methods for promoting skills, abilities, and psychological factors relevant to health-competent physical activity behavior are summarized.

Target group: Rehabilitation patients who are not regularly active or who, due to their health circumstances, need to adapt their regular physical activities.

Content and methods for imparting knowledge about the health effects of physical activity ("effect knowledge")

Effect knowledge describes a person's knowledge of the possible short- and long-term effects of physical activity on physical and mental health, as well as the benefits of promoting physical activity and participation (Tiemann 2006). This knowledge/understanding of the health effects of movement is an important basis for the self-determined planning of health-enhancing physical activity. It can also provide important motivation for regular physical activity (see recommendation C6).

Rehabilitation patients should therefore be informed in particular about the health effects of movement in relation to their individual health problems. Roughly speaking, three areas can be highlighted:

- Information about the effects of different types of movement on health and the progression of chronic diseases
- Information about the optimal amount of movement, e.g., based on general recommendations for health-enhancing movement for the population or based on recommendations for people with specific health problems
- Information about the consequences of physical inactivity and its significance for the progression of chronic diseases

In order to promote the comprehension of this information, the following principles and methods for knowledge dissemination should be taken into account (Huber 2012; Kok et al. 2016; Meng und Faller 2020):

- Use activating teaching methods that encourage in-depth engagement with the information and which go beyond a purely descriptive methodology with passively consumptive learners (e.g., frontal lectures accompanied by slides).
- Take into account or activate the prior knowledge of the rehabilitation patients
- Establish (or allow for) a connection to an individual's personal circumstances and take into account the individual needs and experiences of the rehabilitation patients in order to give the communicated information subjective meaning

Various textbooks and manuals, as well as indication-specific manuals, are available for imparting knowledge about the effects of exercise, providing movement-related materials for rehabilitation patients and therapists (z.B. Pfeifer 2009). For general health effects, reference can be made to the brochure "Menschen in Bewegung bringen" (Getting people moving), which was prepared specifically for multipliers (BZgA 2019) to disseminate the National Recommendations for Physical Activity and Physical Activity Promotion (Rütten und Pfeifer 2017). Given the diversity and specificity of indication-specific information materials, the selection and preparation of materials is the responsibility of those professionals in the rehabilitation team who are responsible for designing this educational content that promotes physical activity.

Information on the organizational form: Establishing a close temporal and spatial proximity between the dissemination of knowledge and the actual performance of physical activities is recommended (Huber 2012; Pfeifer und Sudeck 2020). This allows the short-term positive effects of exercise to be experienced directly and increases information comprehension. This can be achieved by integrating knowledge dissemination sequences into group or individual movement therapy sessions. When imparting knowledge in group discussions or lectures, it is important to ensure consistency and repetition of content and materials and a "common language" within the rehabilitation team in promoting physical activity (see recommendation A4).

Content and methods for teaching and applying knowledge for the health-promoting implementation (planning, implementation, management) of physical activity (“action knowledge”)

Action knowledge describes a person's knowledge of how physical activities can be performed and controlled with a specific (health-oriented) goal in mind (Tiemann 2006). The dissemination of action knowledge can initially take the form of presenting information (e.g., a simple explanation of the principles of physical training, see recommendation C3). However, the application of action knowledge also requires physical and movement-related abilities and skills, such as measuring one's own pulse or assessing one's own exertion during exercise using the Borg scale, for example. Confidence in the independent implementation of health-enhancing physical activity also plays an important role here (see recommendation C4). Therefore, the mere presentation of information is not sufficient to adequately promote control competence. Rather, a combination of physical practice and training with elements of (cognitive and motor) learning is necessary (see recommendation B1). For the didactic and methodological organization of movement-based therapy, this means that the self-realization of forms of movement is directly combined with the communication of relevant information on the effects and implementation of movement. Reflection on content and discussion within the group also have a supportive effect.

The teaching of action knowledge and the development of control competence for performing health-enhancing physical activity are generally characterized by the following features (e.g. Huber 2012):

- The subject matter is presented clearly and with its purpose in mind and can be recognized as a single unit combining knowledge dissemination and practical movement sequences.
- Practical movement tasks are initiated and guided, encouraging participants to experience movement and reflect on their experiences, and providing opportunities for feedback and discussion.
- Opportunities are thus created for intensive engagement with subject matter that go beyond purely practical movement, e.g., through reflection tasks, group discussions, partner work, or accompanying materials.
- Body and movement awareness is incorporated, which is relevant for health-competent control of physical activity. Psychophysical states (e.g., relaxation or activation) can be targeted through variations or contrasts in movement tasks. These states of being can then be used as a basis for reflection and the development of skills and abilities.
- Strategies for implementing what has been learned in everyday life are taught.

Table 5 below contains selected examples of content and methods for developing control competence for health-enhancing physical activity. Specific areas that may be particularly important for rehabilitation patients are presented. Since the transfer of effect and action knowledge is fundamental to the development of control competence, the above information also applies to the selected, more specific content and methods that are specified below.

Table 5: Examples of content and methods for developing control competence for health-enhancing physical activity

Areas	Examples	Sample sources
**Promotion of individual exertion perception and control**		
Use of methods that illustrate the connection between physical exertion and physical reactions and show how physical exertion can be controlled individually	Knowledge dissemination and training in body awareness with a focus on bodily reactions, e.g., heart rate, breathing, muscle tension, and fatigue under different stress stimuli to discuss optimal exertion, overexertion, and under-exertion	(Pfeifer et al. 2009; Thiel et al. 2020)
	Combination of assessments of subjective effort (e.g., using the Borg scale) with objective measurement of physical reactions (heart rate) during physical exertion	(Pfeifer et al. 2009; Thiel et al. 2020)
	Training on how to conduct strength training with different loads, number of repetitions, and assessment of subjective exertion	(Pfeifer et al. 2009)
**Health-promoting design of physical training or everyday physical activities, particularly taking into account indication-specific characteristics**		
Teaching skills for independent planning of strength, endurance, coordination, and balance training	Explanation of relevant principles regarding training load norms for goal-oriented training (scope, duration, intensity, breaks, repetitions)	(ebd.)
	Use of materials for planning and documenting training that can be used and tested by rehabilitation patients during rehabilitation and that can potentially also be used independently after rehabilitation	(ebd.)
Conveying knowledge on the optimal type and dosage of health-enhancing physical activity; raising awareness of individual adjustments for people with health impairments	Information on how to engage in health-enhancing physical activity, e.g., based on evidence-based physical activity recommendations for people with specific health problems; explanations of how to use or adapt physical activity and training recommendations to individual needs	(ebd.)
Teaching indication-specific characteristics for performing everyday physical movements (e.g., climbing stairs for people with hip replacements) and developing individual strategies for optimal planning and design of movement in everyday life, at work, and during leisure time	Information on how to perform everyday movements in a low-impact, joint-friendly fashion, or on movement sequences and postures that may pose a risk to body functions and structures from a biomechanical perspective; targeted practice of motor movement sequences (from reflective practice to automation)	

**Coping with physical discomfort and flexibly adapting exercise to individual health circumstances**		
Use of methods that raise awareness of changes in physical condition and well-being. Methods that raise awareness for the need to ensure appropriate physical activity or an adequate level of physical activity (including a reduction if temporarily required for health reasons)	Sensitization to the recognition of changing symptoms and states of health that are relevant to physical activity (e.g., knowledge of "red flags" that indicate when consultation with medical or therapeutic professionals is advisable)	(Wiskemann and Scharhag-Rosenberger 2014)
	Mindfulness-based methods for promoting conscious self-awareness of bodily signals and psychophysical states of well-being	(Weiss et al. 2023)
	Clarification of the interplay between physical exertion, rest, and symptoms of illness; development of individual strategies for achieving an optimal balance between physical exertion and rest (pacing, energy management with a view to planning and organizing everyday life, work, and leisure time)	(DVGS 2022)
Teaching typical coping and behavior patterns in connection with certain diseases (e.g., fear avoidance/endurance in people with chronic pain)	Knowledge dissemination and sensitization to typical coping and behavior patterns that can have adverse effects on the chronicity of health problems when applied over a longer period of time (e.g., fear of movement, avoidance or endurance beliefs in chronic pain)	(Pfeifer et al. 2007; Semrau et al. 2015)
	Mindfulness training for coping and behavior patterns; if necessary, work toward reevaluating critically experienced situations or develop alternative strategies for physical activation when necessary (e.g., in group discussions)	(Pfeifer et al. 2007; Semrau et al. 2015)
**Regulation and enhancement of psychophysical well-being during and through movement**		
Use of methods to enable individuals to experience changes in their current state of well-being before, during, and after activity, as well as regarding to the type of activity (intensity, type, duration, organizational forms, etc.)	Information on immediate changes in well-being through physical activity; Independent observation and reflection on one's own changes in well-being through physical activity, e.g., through the use of well-being scales before, during, and after physical activity and a targeted variation of type, duration, intensity, rhythm, or organizational forms and settings (e.g., social interaction, experiencing nature, music)	(Pfeifer et al. 2009; Wolf et al. 2020a); (Jones and Zenko 2021)

	Familiarization with strategies for affect-based control of physical activity, e.g., with the help of a so-called “feeling scale,” which is used in addition to body signals (heart rate) or subjective perception of exertion to control exercise intensity	(ebd.)
Use of methods that focus on physical activity as a means of stress management and encourage reflection on the individual recreational potential of physical activity	Teaching about the role of physical activity in stress reactions and stress management	(Fuchs and Gerber 2018)
	Experience stress regulation and relaxation potential by observing physical and psychological reactions to physical activity	(ebd.)

C6: Support motivation through positive experiences with physical activity

(C6) *Movement-based therapy should promote rehabilitation clients' motivation for self-determined, health-enhancing physical activity. In particular, it should enable and raise awareness of positive movement experiences*.

Background

When it comes to continuing physical activity independently after medical rehabilitation, movement-based therapists often notice motivational problems among rehabilitation patients (Deprins et al. 2019). In order to promote movement in movement-based therapy, it is important to take into account the so-called stages of behavior change. Depending on the stage of behavior change, different priorities can be set in the process of behavioral change. Based on the transtheoretical model (Prochaska und DiClemente 1982), a distinction can be made between:

- Individuals who have not yet considered changing their behavior and are generally negative inclined to do so (stage of precontemplation),
- Individuals who are considering behavioral change, but have not yet formed an intention to do so (intentional stage, contemplation, awareness) and
- Individuals who have already formed an intention to change their behavior and need to take further steps to initiate it (preparation stage).

Furthermore, individuals may be sufficiently motivated and already physically active on a regular basis (action stage) or may already be doing so over a longer period of time as a kind of habit (maintenance stage).

In order to progress from the stage of lack of intention (precontemplation) through intention formation and preparation, and finally to taking action, it is essential to determine the extent to which self-efficacy expectations can be built up during rehabilitation (see recommendation C4), positive attitudes and outcome expectations towards physical activity can be developed, and positive affective exercise experiences can be facilitated (see recommendation B2). Together, a solid motivational basis and a strong intention to continue physical activity can be achieved.

For the following content and methods for the practical implementation of the recommendations, it is important to note that intentions can be based on different qualities of motivation, with the degree of self-determination being particularly important (Göhner und Fuchs 2007). While intrinsic motivation is based on incentives directly related to movement (e.g., enjoyable movement execution, aesthetic experiences, positive body experiences), extrinsic incentives can be associated with a variety of outcomes and consequences of physical activity (e.g., social support). Successful motivation aims to increasingly "internalize" the motives for an individual's own physical activity behavior. This means that, for example, it is not only the opinions of others (so-called external motivation mode) or a "guilty conscience" (so-called introjected motivation mode) serving as guiding factors; but that rehabilitants identify with the motives (e.g. "this suits me and is good for me") and that it is really their personal intention to be physically active (so-called identified motivation mode.

In order to achieve increasingly intrinsic motivation and intentions, the basic psychological needs for autonomy, competence, and social integration must be satisfied (Teixeira et al. 2020). Individuals

evaluate exercise experiences as positive when a) the content aligns with their values and ideas and they feel they are involved in the decision-making process (autonomy), b) they feel competent and able to complete the tasks set, and c) they feel socially valued, e.g., through their relationship with members of the therapeutic team or other rehabilitation patients.

Within this context, the following information is provided regarding the recommended pairing of specific target groups with specific content and methods. These contents and methods aim to promote the motivational components of movement-related self-regulation skills (see Recommendation A2).

Target groups: People undergoing rehabilitation who are in the stage of unintentionality (precontemplation) or in the stage of forming intentions (contemplation), as well as those who are in the stage of preparation and whose intrinsic motivation should be strengthened.

Content and methods for educating people about the health risks of physical inactivity (raising awareness of the problem and of risk perceptions)

Initial motivation can come from information about the negative consequences of a lack of physical activity. Essentially, this involves conveying knowledge about the effects of physical activity and the consequences of physical inactivity (see recommendation C5: Knowledge about effects).

- *General risk communication:* Typical content may include information about the links between physical activity and the development of diseases and increased mortality rates. Such elements of knowledge transfer can be aimed at raising awareness of the problems associated with physical inactivity.
- *Personalized risk communication:* Individual information on previous activity behavior (e.g., daily step count or weekly exercise time at least at moderate intensity) or on body functions (e.g., blood pressure, laboratory diagnostics, motor function tests) can also be used to illustrate personal risk. This can be used to stimulate motivation, for example, by addressing discrepancies between current physical activity habits and recommendations on health-enhancing physical activity or by comparing individual results from physical function tests with standardized values.

Methods for raising problem awareness and risk perception can provide initial motivation for people who have not yet shown any intention to increase their physical activity (precontemplation stage) or reinforce the intentions of people who are in the intention-forming phase (contemplation stage).

However, it is also known that sustainable behavioral changes cannot usually be achieved on this rather extrinsic basis of motivation alone (Segar et al. 2016). Nevertheless, they can be used as a starting point for counseling on behavioral change and then combined with strategies to increase self-efficacy and problem solving (Kok et al. 2016).

Contents and methods for changing (cognitive) attitudes and outcome expectations

The intention to regularly engage in physical activity is more probable when the individually perceived health benefits exceed the perceived “costs” (e.g., time, effort, financial costs). Various strategies can be used to influence corresponding (cognitive) attitudes and outcome expectations:

Decision balance (considering advantages and disadvantages). Rehabilitation patients are given the opportunity to discuss and record their own views on the benefits of physical activity (e.g., health benefits, regaining abilities and activities, social participation in sports and exercise groups).

- If positive results from physical activity are closely linked to behavior (e.g., short-term activation or pain relief), incentives for behavioral change are more likely than with (very) long-term expected results (e.g., slowing the progression of a disease).
- Messaging is more motivational when it highlights the benefits of physical activity (e.g., increased mobility in everyday life and at work, or reduced perceived stress) as opposed to only referring to the reduction of risks or the avoidance of possible losses (e.g., prevention of falls or physical deterioration) (Williamson et al. 2021).
- Negative expectations of outcomes ("costs") can be an obstacle to promoting physical activity. These may include fears of physical injury from exercise or the perception of a significant organizational or financial investment. In the rehabilitation process, it is important that negative expectations of outcomes are reduced or put into perspective. This can be achieved, for example, by discussing relevant health information or the potential personal benefits of physical activity (e.g., with regard to low-risk forms of exercise; see recommendation C5 Effect and action knowledge).
- The perception of costs can also be reduced by messaging or statements that make it seem easier to achieve health benefits (principle of low threshold). Examples include statements such as "Every step away from physical inactivity is important and promotes health" from the national exercise recommendations (Pfeifer et al. 2016) or "Doing some physical activity is better than doing none" from the WHO physical activity recommendations (WHO 2020).

Methods that support an individual assessment of the pros and cons of physical activity (decision balance) can clarify one's own motivation and support the development of motivation. Essentially, this is a cognitive-rational strategy for building motivation (see recommendation B2). It can be implemented in individual or group therapy, whereby individual work materials as well as structured group discussions in group therapy with an educational focus can be used.

In cases of strongly negative expectations and perceived barriers, it is important to develop individual solutions for dealing with these barriers to regular physical activity (see recommendation C8: Coping strategies) or for dealing with discomfort (see recommendation C5).

Emphasizing the positive consequences and results of one's own behavior. Strategies that raise awareness of the positive experiences and consequences of physical activity can be seen as an extension of the cognitive-rational approach.

- Therapists create opportunities in which they address (e.g., through feedback) results achieved to date, e.g. regarding health goals (see recommendation C2), and promote satisfaction with what has been achieved. For example, the result of improved physical functioning (e.g., "I can do more repetitions," "I was able to walk 2 km at a stretch") may be directly linked to the physical training of the rehabilitation patients.

These motivational strategies can also be implemented in conjunction with assessment and realized through therapy monitoring and individual feedback (e.g., positive feedback for improved movement technique during strength or coordination training) (see recommendation C1). In communication with rehabilitants, therapists can create opportunities for perceiving positive effects. Rehabilitants' perception should be directed toward the positive results of their own behavior.

Content and methods for facilitating positive (affective) movement experiences (activity-related)

Positive movement experiences can be achieved and made clear directly while performing and experiencing movement. It is therefore less about the medium- or long-term results and consequences of behavior, and more about the activity itself and its immediate, tangible consequences. Often, not only the movement itself, but also the social context is of great importance in experiencing physical activity.

Instruction on the self-observation of immediate emotional consequences. Therapists use methods that highlight changes in well-being immediately after physical activity (e.g., by recording well-being before and after the activity). Immediately experienced positive states of well-being after activities or positive changes in well-being as a result of activities can be strong sources of motivation for future physical activity (see recommendation C5, Table 5).

Strategies for facilitating positive affective-emotional responses to physical activity. Positive reactions to physical activity are not a given, especially for people who were previously physically inactive or who have health impairments. Evidence suggests that certain strategies can increase the likelihood of positive affective-emotional responses (Jones und Zenko 2021, 2023):

- *Outdoor activities* in an environment that is as natural as possible are more likely to lead to positive changes in well-being; therefore, outdoor activities should be incorporated into movement-based therapy programs wherever possible.
- *Utilizing music* can promote a positive affective experience of physical activity; recommendations for music selection are available (Karageorghis et al. 2012).
- *The independent choice of the intensity of physical activity* is associated with more positive affective reactions. The experience of autonomy among rehabilitation patients is essential here; this refers to the subjective feeling of being in control of one's own actions and decisions. This form of stress management can be increasingly incorporated over the course of the rehabilitation process.
- *Enabling experiences of self-efficacy* (e.g., through positive, encouraging feedback) can satisfy the need to experience a sense of competence and thus also promote positive affective responses to physical activity (see recommendation C4 Self-efficacy).
- *The use of new digital technologies* can also be helpful in generating positive experiences with physical activity. Immersive virtual realities can enable a positive affective experience of physical activity; provided that digital options are available, positive affective responses to physical activity can be made tangible.

The strategies described can make positive affective experiences more likely for rehabilitation patients on average. With regard to the goal of enabling independent physical activity, these design options can also be combined with the promotion of control skills for health-enhancing physical activity (see recommendation C5 Control skills).

Strategies for directing attention to the execution of movements (activity-related, body-related). Content and methods that direct attention to the movement itself can be associated with a positive affective experience of movement for some rehabilitation patients. These include:

- Activities that involve “flowing” controlled movements and allow for “immersion” in the movement activity (e.g., qi gong). They are also recognized as mindfulness-based practices in the classification of therapeutic services.

- Activities that involve rhythmic movement patterns (e.g., moving in time to music).
- Group activities that, when performed together, create added value for the experience of the activity (e.g., simple movement sequences in a group) (Molnar-Szakacs et al. 2011).

These strategies can be helpful for rehabilitants in experiencing intrinsic incentives for future physical activity. The perception of these incentives can vary greatly from person to person. In any case, a prerequisite is a sufficient sense of competence in the activities. Building on this, content and methods that are geared toward promoting motivational competence can be implemented (see Recommendation A2, Self-regulation competence, and C8, Finding individual fit, motivational competence).

Play-based approaches in movement-based therapy groups can promote interest in movement tasks and prevent boredom. The playful integration of movement tasks can shift attention away from physical exertion, allowing movement to be viewed more positively. In addition, social interactions can be arranged in a playful manner, which could counteract preexisting negative social experiences in exercise contexts. Examples of suitable playful approaches include:

- "Small games" that require little prior physical and motor experience and no knowledge of the rules of "big games" (such as volleyball, basketball, etc.) (e.g. Moosmann 2017).
- Movement tasks in groups where cooperation is required to complete the tasks.
- Playfully integrated coordination tasks.

For play-based approaches, it is important to ensure that each individual's skills are developed to the best of their ability and that the cooperative nature of the games is emphasized. A competitive nature can be perceived ambivalently in movement-based therapy groups.

Overall, when designing movement-based therapy, it is important to promote a sense of social belonging (Teixeira et al. 2020) and to ensure that the movements performed are free from stigmatization and discrimination based on health impairments or limited previous experience with movement (Thiel et al. 2020).

C7: Align physical activity with individual motives, preferences and prerequisites

(C7) *Movement-based therapy should enable rehabilitation clients to appraise, choose, and engage in forms of physical activity that align with their motives, preferences, and individual capabilities.*

Background

Recent physical activity recommendations suggest that people should choose an activity that they enjoy and find pleasurable (Ladwig and Ekkekakis 2017; Smith et al. 2019). Similarly, movement-based therapists often cite "conveying the joy of movement" as a key focus when asked about important therapeutic elements for successful, sustainable physical activity promotion (Geidl et al. 2019). To achieve this goal, the use of a variety of so-called "small games" (see recommendation C6) is often discussed, which enable shared exercise experiences and social interaction during exercise in a group setting. Another way of implementing the "fun" approach is to introduce rehabilitants to a variety of different forms of movement. Building on this, rehabilitants should be better able to find out for themselves what kind of physical activity they can imagine doing in the future.

Empowering rehabilitants to select and engage in forms of movement consistent with their motives, preferences, and abilities can be structured in such a way as to increase their motivational competence for exercise and movement. Following Rheinberg & Engeser (2010), this means that individuals (a) can identify their own exercise and movement-related motivations and goals, (b) can assess the intrinsic and extrinsic incentives of different activities, and, based on this, (c) can identify and select physical activities that correspond to their own motivations and goals (Schorno et al. 2021). This facet of competence can be understood as a motivational aspect of movement-specific self-regulation competence (see Recommendation A2).

With regard to the respective life circumstances of the rehabilitants, it is helpful to take into account the personal and environmental context factors of each individual. For example, an optimal fit must also be reconciled with the social and spatial conditions in the living environment as well the factual sport and physical activity opportunities close to the home. The latter aspect is also the subject of recommendation C9.

Target group: Rehabilitants who intend to take up or continue physical activity but are unsure which exercise and movement forms appeal to them. Rehabilitants who, due to a change in their health status, are unsure which sports and movement forms are suitable for them or how these could be adapted to their individual capabilities.

Contents and methods for identifying individual motives, goals, and preferences

Rehabilitants examine their own motives, goals, and preferences, for example by completing a corresponding standardized questionnaire and receiving feedback on their motive and goal profile (e.g., discussion of motives and goals that go beyond purely health-related motives) (see Recommendation C1, Assessment: Recording motives, goals, and preferences; e.g., Berner Motive and Goal Inventory for Leisure and Health Sports).

- Therapists discuss previous experiences with exercise/movement as well as preferences and dislikes in an initial consultation and/or assessment.

- Rehabilitants are made aware of the importance of matching individual motives and goals with appropriate forms of exercise and movement. A good match promotes well-being during and as a result of physical activity. This, in turn, is an important prerequisite for sticking with a program.

Content and methods for promoting, testing, and reflecting on diverse movement experiences

- Generally, a large proportion of rehabilitants are introduced to various forms of exercise and movement (e.g., Nordic walking, strength training, aqua gymnastics, etc.) during rehabilitation, and/or they gain a wide range of movement experiences. On this basis, therapists encourage targeted reflection and discussion about the experiences rehabilitants have with different forms of movement and how they "typically" experience them.
- During rehabilitation, rehabilitants are encouraged to reflect on the various forms of exercise and movement in light of their own motives, goals, and preferences, enabling them to identify the physical activities that are right for them.
- Once individual goals, motives, and preferences have been determined, specific therapy options and suitable forms of movement can be recommended for testing and/or selected for the rehabilitation period.

The methods and content mentioned are closely related to the formulation of behavioral goals in Recommendation C2 (they also create a prerequisite for further planning of courses of action (see Recommendation C8) as well as consultations and direct preparations (e.g., contacting local providers) for continuing physical activity after medical rehabilitation or in rehabilitation aftercare (see Recommendation C9).

The above-mentioned content and methods can be implemented in a structured manner in movement therapy group programs. Implementation is facilitated by supporting work materials for rehabilitants and therapists (e.g. Schmid et al. 2024). Within the group, individual diagnostics of motives, goals, and preferences as well as feedback on the results of the diagnostics can be integrated for the rehabilitants. In some cases, the content and methods are also implemented with a slightly greater time investment through a combination of group offerings and individual counseling (Schmid et al. 2023).

C8: Support action planning and coping planning

(C8) *Movement-based therapy should enable rehabilitation clients to translate their intentions regarding physical activity into actual, regular behavior. This includes overcoming internal and external barriers and shielding their physical activity intentions from competing activities.*

Background

A common phenomenon in physical activity behavior is the failure to put intentions into practice. This phenomenon is described in health behavior research as the "intention-behavior gap." Various studies on physical activity behavior report that around 50% of people who have set themselves an physical activity-related goal have not achieved this goal after several weeks or months (Rhodes and Bruijn 2013). Although these individuals have made progress in the motivational processes involved in setting goals, a lack of success in the volitional processes involved in pursuing goals is evident. Either the implementation of the set goal has not yet begun, or the behavior begins to fluctuate again after initial successful initiatives, or is discontinued altogether. A distinction is made here between volitional problems of initiation and stabilization of physical activity behavior (Sudeck 2006).

Individuals often fail to overcome situational barriers that prevent them from engaging in physical activity, or are unable to shield their intentions from competing alternatives. These can be physical barriers (e.g., not feeling fit or experiencing pain) or psychosocial barriers (e.g., listlessness, stress, many other professional or family obligations) (Krämer und Fuchs 2010). Achieving goals can then be jeopardized by the fact that individuals tend to remain inactive (e.g., resting on the sofa or in front of the TV) or prioritize other activities over physical activity (e.g., work or family activities). In such cases, it may not be possible to reconcile the time required for physical activity with other commitments.

A number of research findings are now available on the effectiveness of so-called *volitional strategies* for supporting goal pursuit (Geidl 2014; Jung 2024). They show that content and methods for action and coping planning as well as for self-observation of behavior or behavioral outcomes are promising. Rehabilitants can be supported in strengthening volitional processes through,

- therapists using and teaching certain volitional techniques for behavioral change during rehabilitation (e.g., discussing and jointly recording specific planning steps for behavioral implementation).
- enabling rehabilitants to use strategies that are suitable for them in a manner appropriate to their individual circumstances, even after rehabilitation (see recommendation A2, movement-specific self-regulation skills)

Target group: Rehabilitants who are already motivated to change their behavior and can formulate initial physical activity-related goals and intentions. This recommendation is closely related to recommendation C2, in that it now involves supporting the implementation of a medium- to long-term behavioral goal.

Contents and methods for action planning and coping strategies

Action and coping planning strategies comprise methods for supporting the actual implementation of objectives ("from intention to implementation") (e.g., Krämer and Göhner 2020).
The core of action planning is the specification of movement-related goals, upon which an action plan is outlined. The "5 W questions" are helpful for this purpose: What? Where? When? With whom? and How? (Göhner und Fuchs 2007). Therapists can guide rehabilitants in drawing up their own action

plans. Suitable physical activity should be identified and selected. A concrete plan should then be formulated as to when, where, and how the specified physical activity is to be carried out. The action plan should also consider the period after the rehabilitation stay.

Coping planning is related to what is known as barrier management (Krämer and Fuchs 2010). To be able to effectively implement the action plan formulated above, potential barriers should be identified and concrete coping strategies developed to overcome them. In many cases, these are situational barriers that rehabilitants are familiar with from past experiences. Coping planning includes:

- *Acute strategies*, i.e., when the individual finds themselves in a "risk situation" for an inactive alternative instead of a planned physical activity. For example, re-motivating oneself by reminding oneself of the benefits of exercise, reinterpreting difficult situations as challenges ("I would be quite proud of myself if I could manage this now.") or directing attention specifically to information that promotes the desired behavior (e.g., focusing on your own exercise plans and blocking out information that may interfere with implementation, such as TV programs or social media use).
- *Preventive strategies* aimed at preemptively avoiding such risk situations (e.g., through reminders such as entries in one's own calendar, through preparations such as taking sports equipment to work, or through social integration by making arrangements with friends to exercise or participating in group activities).

For movement-based therapy, there are some working materials available that can be used for action and coping planning for and with rehabilitants (e.g., Pfeifer 2009). These are often integrated into more comprehensive programs designed to support behavioral change (see Table 2 in Recommendation C2). In these application examples, written working documents are used to support the action and coping planning process. The forms of organization vary, with individual action and coping planning appearing feasible in groups of up to 10-12 individuals. For the practical implementation of the programs mentioned, closed groups are usually provided for, and in some cases combinations with individual counseling or (telephone) follow-up contacts are also implemented. In some cases, the programs are supported by digital tools.

Contents and methods for self-observation, self-assessment, and self-reward

In the process of changing movement behavior, self-observation methods are usually used based on goal formulation and the development of action and coping plans. Self-observation methods aim to compare actual implementation (current status) with intended plans (target status). If this comparison yields a positive assessment, it can strengthen the motivation to pursue the goal (e.g., due to pride and satisfaction with what has been achieved). More negative assessments of this comparison can be a starting point for intensifying efforts. If necessary, more negative assessments can also lead to adjustments in goals, action plans, and coping strategies.

Self-observation of behavior. Methods of self-observation of behavior include activity diaries and training documentation. They can also be integrated into electronic systems (e.g., fitness trackers and smartphones) as applications. These are used to compare goals and action plans with actual behavior and encourage reflection on one's own behavioral implementation. Exercise therapists can familiarize rehabilitants with these methods during rehabilitation and highlight the potential for successfully pursuing goals independently. Methods for self-observation of behavior are also frequently integrated

into working materials or digital tools designed to support behavioral change in exercise and movement-based therapy (e.g. Pfeifer 2009).

- *Self-reward.* Positive self-evaluations when successfully pursuing (partial) goals can lead to reinforced behavior, as emotions such as pride and satisfaction are perceived as rewarding. In addition, rehabilitants can be made aware that so-called extrinsic self-rewards can also be useful for staying on track. These are rewards that you set for yourself and "treat" yourself to when you have achieved certain (sub)goals (e.g., a purchase for your favorite hobby).
- *Dealing with setbacks, relapse prevention.* Given the well-known intention-behavior gap, "lapses" (Marlatt, G.A., Gorden, J.R. 1985) and relapses into inactive episodes are part of a longer-term process of behavioral change. Therapists should raise awareness that temporary illnesses, vacations, stressful phases, etc. can lead to such situations and that a temporary relapse into a phase of less activity is not unusual. Self-observation is the basis for investigating the causes in a more targeted manner and taking appropriate countermeasures. In these phases, conscious action and coping planning can contribute to a proactive approach to dealing with failures (e.g., slip-ups as a reason for increased mindfulness instead of resignation; Krämer and Göhner 2020).

Further content and methods to support behavioral change

- *Encourage social support.* Behavioral change can benefit in many ways from social support from the rehabilitant's environment. Such elements of social support have already been addressed in the content *and* methods for action and coping planning (e.g., selection of individuals for cooperative physical activity as a preventive coping strategy or as an aspect of action planning). Given its relevance for behavioral change, therapists should raise awareness of the potential of social support and encourage its use.
- *Create commitment with behavior contracts.* In consultation with and under the guidance of therapists, rehabilitants can commit in writing to achieving independently formulated activity goals for a specific period of time. A "contractual agreement" can be used for this purpose (Geidl et al. 2014; Vries et al. 2015; Ma et al. 2019; Willett et al. 2021). However, it should be borne in mind that some individuals may perceive a behavioral contract as restrictive and negative. Therapists should therefore inquire about individuals' willingness to enter into such a behavioral contract.

C9: Facilitate continuation after rehab

(C9) *In movement-based therapy, rehabilitation clients should receive comprehensive counseling about physical activity opportunities for the period following their medical rehabilitation. This includes information on follow-up rehabilitation programs, participation in health enhancing physical activity programs, as well as other suitable physical activity options*

Background

Rehabilitation aftercare services are extremely important for maintaining the effects achieved in medical rehabilitation and for the long-term pursuit of individually formulated goals (see recommendation C2). This is particularly important in order to build on the forms of physical and sporting activity - as well as exercise and training forms - learned and practiced during rehabilitation, and to continue to support those activities after the stay in the rehabilitation facility. For this reason, rehabilitants should also be comprehensively informed and advised about the possibilities for continuing their physical activity from the perspective of movement-based therapy.

Target Group: All rehabilitants.

Movement activities and opportunities

At the end of the stay in the rehabilitation facility, the following individual or group movement therapy programs are particularly recommended:

- the aftercare services provided by the German pension insurance system, such as IRENA and T-RENA, and the possibilities of digital rehabilitation aftercare. Together with the rehabilitants, services close to their place of residence can be identified via the platform www.nachderreha.de.
- The wide range of rehabilitation exercise and functional training programs, which are implemented in accordance with the joint framework recommendations of the Federal Working Group for Rehabilitation (www.bar-frankfurt.de). The framework recommendations, information for insurance holders and contact addresses for providers can be found on the following website: https://www.deutsche-rentenversicherung.de/DRV/DE/Reha/Reha-Nachsorge/Reha-Sport/reha-sport_node.html
- The wide range of health-related exercise programs offered by sports clubs, particularly those that have been awarded the SPORT PRO GESUNDHEIT quality seal by the German Olympic Sports Confederation (DOSB), see https://service-sportprogesundheit.de/ . Offers close to home can be found together with rehabilitants using the DOSB's exercise map: https://bewegungslandkarte.de/.
- The wide range of services offered by commercial fitness and health facilities. These can be particularly suitable for continuing independent exercise if professionally qualified support is available from individuals with expertise in exercise (sports science, physiotherapy). Rehabilitants can be advised to look for appropriate qualified support or to inquire about this before or when visiting a facility. Quality seals from recognized institutions can also provide information about quality-assured procedures.
- So-called digital health applications are also increasingly available, which can be helpful in promoting movement and maintaining physical activity. Such applications can be found on the platform of the Federal Institute for Drugs and Medical Devices (https://diga.bfarm.de/de). Their suitability for rehabilitation aftercare can then be assessed on the basis of a professional

evaluation by movement-based therapists (together with other members of the rehabilitation team, if necessary) and in consultation with the rehabilitants.

After medical rehabilitation and depending on the individual's health status, the widespread health courses and preventive services offered by health insurance companies under Section 20 of the German Social Code (SGB V) may also be a way to obtain further support for continuing independent exercise. Many health insurance companies have search portals on their websites where you can find relevant offers. In addition, various self-help organizations also offer advice on exercise or contact with exercise providers.

Literature

Baldus A, Huber G, Pfeifer K, Schüle K, 2012. ICF-Orientierung in der Sport- und Bewegungstherapie: Neue Versorgungspfade für die Rehabilitation. B&G Bewegungstherapie und Gesundheitssport, 28 (02), 85–89.

Barker J, Smith Byrne K, Doherty A, Foster C, Rahimi K, Ramakrishnan R, Woodward M, Dwyer T, 2019. Physical activity of UK adults with chronic disease: cross-sectional analysis of accelerometer-measured physical activity in 96 706 UK Biobank participants. Int J Epidemiol, 48 (4), 1167–1174.

Bauman AE, Reis RS, Sallis JF, Wells JC, Loos RJF, Martin BW, 2012. Correlates of physical activity. Why are some people physically active and others not? Lancet, 380 (9838), 258–271.

Berg K, 1989. Measuring balance in the elderly: preliminary development of an instrument. Physiotherapy Canada, 41 (6), 304–311, https://utpjournals.press/doi/10.3138/ptc.41.6.304

Bjarnason-Wehrens B, Schulz O, Gielen S, Halle M, Dürsch M, Hambrecht R, Lowis H, Kindermann W, Schulze R, Rauch B, 2009. Leitlinie körperliche Aktivität zur Sekundärprävention und Therapie kardiovaskulärer Erkrankungen. Clinical Research in Cardiology Supplements, 4 (S3), 1–44.

Bös K (Hrsg.), 2017. Handbuch Motorische Tests. Sportmotorische Tests, motorische Funktionstests, Fragebögen zur körperlich-sportlichen Aktivität und sportpsychologische Diagnoseverfahren (3., überarbeitete und erweiterte Auflage). Hogrefe, Göttingen, 899 S.

Bös K, Abel T, Woll A, Niemann S, Tittlbach S, Schott N, 2002. Der Fragebogen zur Erfassung des motorischen Funktionsstatus (FFB-Mot). Diagnostica, 48 (2), 101–111.

Bösch D, Criée C-P, 2020. 6-Minuten-Gehtest. In: Lungenfunktionsprüfung. Springer Berlin Heidelberg, Berlin, Heidelberg, S. 199–204.

Bovend'Eerdt TJH, Botell RE, Wade DT, 2009. Writing SMART rehabilitation goals and achieving goal attainment scaling: a practical guide. Clinical rehabilitation, 23 (4), 352–361.

Brähler E, Strauß B, Hessel A, Schumacher J, 2000. Normierung des Fragebogens zur Beurteilung des eigenen Körpers (FBeK) an einer repräsentativen Bevölkerungsstichprobe. Diagnostica, 46 (3), 156–164.

Brand R, 2006. Die affektive Einstellungskomponente und ihr Beitrag zur Erklärung von Sportpartizipation. Z Sportpsychol, 13 (4), 147–155.

Brand R, Ekkekakis P, 2018. Affective–Reflective Theory of physical inactivity and exercise. German Journal of Exercise and Sport Research, 48 (1), 48–58.

Brand R, Sudeck G, Ekkekakis P, 2023. Entwicklung und Validierung einer deutschsprachigen Fassung des ‚Affective Exercise Experiences Questionnaire' (AFFEXX-G). https://osf.io/29jhe/.

Brawner CA, Churilla JR, Keteyian SJ, 2016. Prevalence of physical activity is lower among individuals with chronic disease. Medicine and science in sports and exercise, 48 (6), 1062–1067.

Brüggemann S, Sewöster D, Kranzmann A, 2018. Bewegungstherapeutische Versorgung in der medizinischen Rehabilitation der Rentenversicherung – eine Analyse auf Basis quantitativer Routinedaten. Die Rehabilitation, 57 (1), 24–30.

BZgA, 2019. Menschen in Bewegung bringen. https://shop.bioeg.de/menschen-in-bewegung-bringen-60640104/.

Carl J, Sudeck G, Geidl W, Schultz K, Pfeifer K, 2021. Competencies for a Healthy Physically Active Lifestyle-Validation of an Integrative Model. Research quarterly for exercise and sport, 92 (3), 514–528.

Carl J, Sudeck G, Pfeifer K, 2020. Competencies for a healthy physically active lifestyle-reflections on the model of Physical Activity-Related Health Competence. J Phys Act Health, 17 (7), 688–697.

Darker CD, French DP, Eves FF, Sniehotta FF, 2010. An intervention to promote walking amongst the general population based on an 'extended' theory of planned behaviour: a waiting list randomised controlled trial. Psychology & health, 25 (1), 71–88.

Dean E, 2009. Physical therapy in the 21st century (Part I): toward practice informed by epidemiology and the crisis of lifestyle conditions. Physiother Theory Pract, 25 (5-6), 330–353.

Dean E, Al-Obaidi S, Andrade AD de, Gosselink R, Umerah G, Al-Abdelwahab S, Anthony J, Bhise AR, Bruno S, Butcher S, Fagevik-Olsén M, Frownfelter D, Gappmaier E, Gylfadóttir S, Habibi M, Hanekom S, Hasson S, Jones A, LaPier T, Lomi C, Mackay L, Mathur S, O'Donoghue G, Playford K, Ravindra S, Sangroula K, Scherer S, Skinner M, Wong WP, 2011. The first physical therapy summit on global health. implications and recommendations for the 21st century. Physiother Theory Pract, 27 (8), 531–547.

Deprins J, Geidl W, Streber R, Pfeifer K, Sudeck G, 2019. Konzeptionelle Grundlagen der Bewegungstherapie in der medizinischen Rehabilitation. Ergebnisse einer bundesweiten Bestandsaufnahme. Die Rehabilitation.

Deutsche Rentenversicherung, 2014. Klassifikation therapeutischer Leistungen in der medizinischen Rehabilitation (KTL) (Ausgabe 2015), 27.03.2018, https://www.deutsche-rentenversicherung.de/Allgemein/de/Inhalt/3_Infos_fuer_Experten/01_sozialmedizin_forschung/downloads/sozmed/klassifikationen/dateianhaenge/KTL/ktl_2015_pdf.pdf?__blob=publicationFile&v=8

Deutsche Rentenversicherung Bund, 2009. Rahmenkonzept zur medizinischen Rehabilitation in der gesetzlichen Rentenversicherung. Abgerufen am 31.08.2017, http://www.deutscherentenversicherung.de/cae/servlet/contentblob/207034/publicationFile/2130/rahmenkonzept_lta_datei.pdf

Deutsche Rentenversicherung Bund, 2021. Curriculum Generische Selbstmanagementmodule (SelMa). Standardisiertes Gruppenprogramm, https://www.deutsche-rentenversicherung.de/SharedDocs/Downloads/DE/Experten/infos_reha_einrichtungen/gesundheitstraining/generische_Selbstmanagementmodule_SelMa.pdf?__blob=publicationFile&v=1

Deutsche Rentenversicherung Bund, 2023. Leitfaden Berufsgruppen in der medizinischen Rehabilitation und ihre interprofessionelle Zusammenarbeit, https://www.deutsche-rentenversicherung.de/SharedDocs/Downloads/DE/Experten/infos_fuer_aerzte/veranstaltungen/leitfaden_Berufsgruppen_IZ.html

Dibbelt S, Greitemann B, 2011. Multimodale Rehabilitation bei chronischen Rückenschmerzen: Konzepte, Wirksamkeit und Umsetzung. Public Health Forum, 19 (4), 9–10.

Dibben GO, Gardiner L, Young HM, Wells V, Evans RA, Ahmed Z, Barber S, Dean S, Doherty P, Gardiner N, Greaves C, Ibbotson T, Jani BD, Jolly K, Mair FS, McIntosh E, Ormandy P, Simpson SA, Ahmed S, Krauth SJ, Steell L, Singh SJ, Taylor RS, Begum S, DeBarros C, Davies F, Sterniczuk K, Kumar R, Longley R, Freeman A, Lalseta J, Ashby P, van Grieken M, Grace Elder D, 2024. Evidence for exercise-based interventions across 45 different long-term conditions: an overview of systematic reviews. eClinicalMedicine, 102599.

Dutton GR, Tan F, Provost BC, Sorenson JL, Allen B, Smith D, 2009. Relationship between self-efficacy and physical activity among patients with type 2 diabetes. Journal of behavioral medicine, 32 (3), 270–277.

DVGS (Deutscher Verband für Gesundheitssport und Sporttherapie), 2022. Factsheet. Bewegung und körperliches Training nach Covid-19, https://dvgs.de/images/2022/06/DVGS_09_Long_Covid_Factsheet_Finalversion_Web.pdf

Ekkekakis P, Barker JL, Zenko Z, Werstein KM, 2024. Exercise in Obesity from the Perspective of Dual-Process Theories. In: Razon S, Sachs ML (Hrsg.). Applied Exercise Psychology. Routledge, New York, S. 337–371.

Ekkekakis P, Zenko Z, Vazou S, 2021. Do you find exercise pleasant or unpleasant? The Affective Exercise Experiences (AFFEXX) questionnaire. Psychol Sport Exerc, 55, 101930.

Elsman EB, Leerlooijer JN, Beek J ter, Duijzer G, Jansen SC, Hiddink GJ, Feskens EJ, Haveman-Nies A, 2014. Using the intervention mapping protocol to develop a maintenance programme for the SLIMMER diabetes prevention intervention. BMC public health, 12.

Elvén M, Hochwälder J, Dean E, Söderlund A, 2015. A clinical reasoning model focused on clients' behaviour change with reference to physiotherapists. its multiphase development and validation. Physiother Theory Pract, 31 (4), 231–243. Abgerufen am 18.01.2018.

Farin E, 2014. Patientenorientierung in der Rehabilitation. Zeitschrift für Rheumatologie, 73 (1), 35–41. Abgerufen am 03.06.2014.

Focht BC, Brawley LR, Rejeski WJ, Ambrosius WT, 2004. Group-mediated activity counseling and traditional exercise therapy programs: Effects on health-related quality of life among older adults in cardiac rehabilitation. Annals of behavioral medicine : a publication of the Society of Behavioral Medicine, 28 (1), 52–61, https://academic.oup.com/abm/article/28/1/52-61/4633684

Freund H, 2017. Geriatrisches Assessment und Testverfahren: Grundbegriffe - Anleitungen - Behandlungspfade (3., erweiterte und aktualisierte Auflage). Verlag W. Kohlhammer, Stuttgart, 283 S.

Froböse I, Wilke C, Abel R (Hrsg.), 2022. Trainingstherapie in der Rehabilitation. Konzepte, Therapie und Übungen. Elsevier, München, 564 S.

Fuchs R, 1997. Psychologie und körperliche Bewegung. Grundlagen für theoriegeleitete Interventionen. Hogrefe Verl. für Psychologie, Göttingen, 332 S.

Fuchs R, Gerber M (Hrsg.), 2018. Handbuch Stressregulation und Sport. Springer Berlin Heidelberg, Berlin, Heidelberg.

Fuchs R, Klaperski S, Gerber M, Seelig H, 2015. Messung der Bewegungs- und Sportaktivität mit dem BSA-Fragebogen. Zeitschrift für Gesundheitspsychologie, 23 (2), 60–76. Abgerufen am 21.10.2016.

Garstang KR, Jackman PC, Healy LC, Cooper SB, Magistro D, 2024. What effect do goal setting interventions have on physical activity and psychological outcomes in insufficiently active adults? A systematic review and meta-analysis. Journal of physical activity & health, 21 (6), 541–553.

Geidl W, Abu-Omar K, Weege M, Messing S, Pfeifer K, 2020a. German recommendations for physical activity and physical activity promotion in adults with noncommunicable diseases. Int J Behav Nutr Phys Act, 17 (1), 12.

Geidl W, Schlesinger S, Mino E, Miranda L, Pfeifer K, 2020b. Dose–response relationship between physical activity and mortality in adults with noncommunicable diseases: a systematic review and meta-analysis of prospective observational studies. International Journal of Behavioral Nutrition and Physical Activity, 17 (1), 1.

Geidl W, Semrau J, Pfeifer K, 2014. Health behaviour change theories: contributions to an ICF-based behavioural exercise therapy for individuals with chronic diseases. Disability and Rehabilitation, 36 (24), 2091–2100.

Geidl W, Sudeck G, Wais J, Pfeifer K, 2022. Bewegungsförderliche Bewegungstherapie in der medizinischen Rehabilitation: Konsequenzen der bundesweiten Bestandsaufnahme für die Qualitätsentwicklung [Physical Activity Promotion in Exercise Therapy in Medical Rehabilitation: Consequences of the Nationwide Survey for Quality Development]. Rehabilitation (Germany), 61 (5), 336–343.

Geidl W, Wais J, Fangmann C, Demisse E, Pfeifer K, Sudeck G, 2019. Physical activity promotion in daily exercise therapy: the perspectives of exercise therapists in German rehabilitation settings. BMC Sports Sci Med Rehabil, 11, 28. Abgerufen am 17.12.2019.

Glattacker M, Farin-Glattacker E, Quaschning K, Dibbelt S, 2015. Arbeitsbuch Reha-Ziele. Zielbvereinbarungen in der medizinischen Rehabilitation.

Göhner W, Fuchs R, 2007. Änderung des Gesundheitsverhaltens. MoVo-Gruppenprogramme für körperliche Aktivität und gesunde Ernährung. Hogrefe, Göttingen, viii, 179.

Göhner W, Seelig H, Fuchs R, 2009. Intervention Effects on Cognitive Antecedents of Physical Exercise: A 1-Year Follow-Up Study. Applied Psychology: Health and Well-Being, 1 (2), 233–256.

Golightly YM, Allen KD, Ambrose KR, Stiller JL, Evenson KR, Voisin C, Hootman JM, Callahan LF, 2017. Physical activity as a vital sign. A systematic review. Preventing Chronic Disease, 14, E123. Abgerufen am 20.03.2019.

Gothe NP, Erlenbach E, Engels H-J, 2022. Exercise and self-esteem model: Validity in a sample of healthy female adolescents. Current Psychology, 41 (12), 8876–8884.

Härter M, Dirmaier J, 2022. Interaktion und Kommunikation. In: Meyer, T., Bengel, J., Wirtz, A. (Hrsg.). Lehrbuch der Rehabilitationswissenschaften. Hogrefe-Verlag, Bern.

Hasler G, Klaghofer R, Buddeberg C, 2003. Der Fragebogen zur Erfassung der Veränderungsbereitschaft (FEVER) - Testung der deutschen Version der University of Rhode Island Change Assessment Scale (URICA) -. Psychotherapie, Psychosomatik, medizinische Psychologie, 53 (9-10), 406–411.

Higgins TJ, Middleton KR, Winner L, Janelle CM, 2014. Physical activity interventions differentially affect exercise task and barrier self-efficacy: a meta-analysis. Health psychology : official journal of the Division of Health Psychology, American Psychological Association, 33 (8), 891–903.

Hohmann A, Lames M, Letzelter M, Pfeiffer M, 2020. Einführung in die Trainingswissenschaft (7., überarbeitete Auflage). Limpert Verlag, Wiebelsheim, 424 S.

Hossner E-J, Künzell S, 2022. Einführung in die Bewegungswissenschaft. Limpert Verlag, Wiebelsheim, 502 S.

Howlett N, Trivedi D, Troop NA, Chater AM, 2018. Are physical activity interventions for healthy inactive adults effective in promoting behavior change and maintenance, and which behavior change techniques are effective? A systematic review and meta-analysis. Translational behavioral medicine, 9 (1), 147–157.

Huber G, 2012. Zur pädagogischen Dimension der Sporttherapie. In: Schüle K, Huber G (Hrsg.). Grundlagen der Sport- und Bewegungstherapie. Prävention, ambulante und stationäre Rehabilitation. (3., vollständig überarb. und erw. Aufl.). Deutscher Ärzte-Verlag, Köln, S. 122–133.

Huy C, 2011. German-PAQ-50+ - German-PAQ-50+ Fragebogen zur Erfassung der körperlichen Aktivität, unveröffentlicht.

Jones L, Zenko Z, 2021. Strategies to facilitate more pleasant exercise experiences. In: Zenko Z, Jones L (Hrsg.). Essentials of exercise and sport psychology: An open access textbook. Society for Transparency, Openness, and Replication in Kinesiology, S. 242–270.

Jones L, Zenko Z, 2023. A systematic narrative review of extrinsic strategies to improve affective responses to exercise. Front Sports Act Living, 5, 1186986.

Jung A, Geidl W, Matting L, Hoessel L-M, Siemens W, Sudeck G, Pfeifer K, 2024. Efficacy of physical activity promoting interventions in physical therapy and exercise therapy for persons with noncommunicable diseases: an overview of systematic reviews. Physical Therapy, 104 (7).

Jung A, Matting L, Sudeck G, Geidl W, Pfeifer K, 2023. Physical activity promotion by physical and exercise therapists: A protocol for a scoping review and content analysis, 10.17605/OSF.IO/AXZSJ.

Kabat-Zinn J, 2003. Mindfulness-based interventions in context: Past, present, and future. Clinical Psychology: Science and Practice, 10 (2), 144–156.

Karageorghis CI, Terry PC, Lane AM, Bishop DT, Priest D, 2012. The BASES Expert Statement on use of music in exercise. Journal of sports sciences, 30 (9), 953–956.

Kayes NM, Papadimitriou C, 2023. Reflecting on challenges and opportunities for the practice of person-centred rehabilitation. Clinical Rehabilitation, 37 (8).

Kok G, Gottlieb NH, Peters G-JY, Mullen PD, Parcel GS, Ruiter RAC, Fernández ME, Markham C, Bartholomew LK, 2016. A taxonomy of behaviour change methods: an Intervention Mapping approach. Health psychology review, 10 (3), 297–312.

Kolb DA, 2015. Experiential learning. Experience as the source of learning and development (Second edition). Pearson Education LTD, Upper Saddle River, New Jersey.

Körner M, 2022. Reha-Team. In: Meyer, T., Bengel, J., Wirtz, A. (Hrsg.). Lehrbuch der Rehabilitationswissenschaften. Hogrefe-Verlag, Bern.

Krämer L, Fuchs R, 2009. Skalen zu den sportbezogenen situativen Barrieren und dem sportbezogenen Barrierenmanagement. Abgerufen am 08.05.2014.

Krämer L, Fuchs R, 2010. Barrieren und Barrierenmanagement im Prozess der Sportteilnahme. Zeitschrift für Gesundheitspsychologie, 18 (4), 170–182.

Krämer L, Göhner W, 2020. Handlungsplanung, Barrieren und Barrierenmanagement. In: Bengel J, Mittag O (Hrsg.). Psychologie in der medizinischen Rehabilitation. Springer Berlin Heidelberg, Berlin, Heidelberg.

Ladwig M, Ekkekakis P, 2017. Affect-based exercise prescription. An idea whose time has come? ACSM's Health Fitness J, 21 (5).

Laitakari J, Asikainen T-M, 1998. How to promote physical activity through individual counseling—A proposal for a practical model of counseling on health-related physical activity. Patient education and counseling, 33, S13-S24.

Lehnert K, Sudeck G, Conzelmann A, 2011. BMZI – Berner Motiv- und Zielinventar im Freizeit- und Gesundheitssport. Diagnostica, 57 (3), 146–159.

Lein DH, Clark D, Graham C, Perez P, Morris D, 2017. A model to integrate health promotion and wellness in physical therapist practice: development and validation. Physical therapy, 97 (12).

Lobelo F, Rohm Young D, Sallis R, Garber MD, Billinger SA, Duperly J, Hutber A, Pate RR, Thomas RJ, Widlansky ME, McConnell MV, Joy EA, 2018. Routine assessment and promotion of physical activity in healthcare settings. a scientific statement from the American Heart Association. Circulation, 137 (18), e495-e522.

Ma JK, West CR, Martin Ginis KA, 2019. The effects of a patient and provider co-developed, behavioral physical activity intervention on physical activity, psychosocial predictors, and fitness in individuals with spinal cord injury: a randomized controlled trial. Sports Medicine, 49 (7), 1117–1131.

Marks-Vieveen JM, Uijtdewilligen L, Motazedi E, Stijnman DPM, van den Akker-Scheek I, Bouma AJ, Buffart LM, Groot V de, Hollander E de, Jelsma JGM, Jong J de, van Keeken HG, Krops LA, van der Leeden M, Loer SA, van Mechelen W, van Nassau F, Nauta J, Verhagen E, Wendel-Vos W, van der Woude LHV, Zwerver J, Dekker R, van der Ploeg HP, 2024. Physical Activity Levels, Correlates, and All-Cause Mortality Risk in People Living With Different Health Conditions. Journal of physical activity & health, 21 (4), 394–404.

Marlatt, GA, Gorden, JR, 1985. Relapse prevention: Maintenance strategies in the treatment of addictive behaviors. Guilford, New York.

Matting, L, Pfeifer, K, Sudeck, G, Jung, A, Langhirt, F, Geidl, W Physical activity promotion in physical therapy, exercise therapy and other movement-based therapies: A scoping review and content analysis of interventional concepts [im Review]

McAuley E, Katula J, Mihalko SL, Blissmer B, Duncan TE, Pena M, Dunn E, 1999. Mode of physical activity and self-efficacy in older adults: a latent growth curve analysis. The journals of gerontology. Series B, Psychological sciences and social sciences, 54 (5), P283-92.

McEwan D, Harden SM, Zumbo BD, Sylvester BD, Kaulius M, Ruissen GR, Dowd AJ, Beauchamp MR, 2016. The effectiveness of multi-component goal setting interventions for changing physical activity behaviour: a systematic review and meta-analysis. Health psychology review, 10 (1), 67–88. Abgerufen am 27.09.2017.

McGrane N, Galvin R, Cusack T, Stokes E, 2015. Addition of motivational interventions to exercise and traditional physiotherapy: a review and meta-analysis. Physiotherapy, 101 (1), 1–12.

Meng K, Faller H, 2020. Patientenschulung und Gesundheitskompetenz. In: Bengel J, Mittag O (Hrsg.). Psychologie in der medizinischen Rehabilitation. Springer Berlin Heidelberg, Berlin, Heidelberg, S. 149–160.

Messner T, 2018. Motivational Interviewing in der Sport- und Bewegungstherapie – ein Ansatz zur Förderung der intrinsischen Motivation. B&G Bewegungstherapie und Gesundheitssport, 34 (04), 186–193.

Michie S, van Stralen MM, West R, 2011. The behaviour change wheel: a new method for characterising and designing behaviour change interventions. Implementation Science, 6, 42.

Miller WR, 2014. Motivierende Gesprächsführung (4. Aufl.). Lambertus Verlag, s.l.

Molnar-Szakacs I, Assuied VG, Overy K, 2011. Shared affective motion experience (SAME) and creative, interactive music therapy. In: Hargreaves D, Miell D, MacDonald R (Hrsg.). Musical ImaginationsMultidisciplinary perspectives on creativity, performance and perception. Oxford University Press, S. 313–331.

Moore CL, Kaplan SL, 2018. A framework and resources for shared decision making: opportunities for improved physical therapy outcomes. Physical Therapy, 98 (12), 1022–1036. Abgerufen am 1.

Moosmann K (Hrsg.), 2017. Das große Limpert-Buch der kleinen Spiele. Bewegungsspaß für Jung und Alt (4., erweiterte Auflage). Limpert Verlag, Wiebelsheim, 461 S.

Paulsen L, Benz L, Vonstein C, Bucksch J, 2022. Erhebungsinstrumente zur Erfassung der kommunalen Bewegungsumwelt älterer Menschen – eine systematische Betrachtung. Prävention und Gesundheitsförderung, 17 (2), 200–207.

Pedersen BK, Saltin B, 2015. Exercise as medicine - evidence for prescribing exercise as therapy in 26 different chronic diseases. Scand J Med Sci Sports, 25 Suppl 3, 1–72.

Pelliccia A, Sharma S, Gati S, Bäck M, Börjesson M, Caselli S, Collet J-P, Corrado D, Drezner JA, Halle M, Hansen D, Heidbuchel H, Myers J, Niebauer J, Papadakis M, Piepoli MF, Prescott E, Roos-Hesselink JW, Graham Stuart A, Taylor RS, Thompson PD, Tiberi M, Vanhees L, Wilhelm M, 2021. 2020 ESC Guidelines on sports cardiology and exercise in patients with cardiovascular disease. European heart journal, 42 (1), 17–96.

Pfeffer I, Wagner M, 2020. Modelle zur Erklärung der Veränderung von Gesundheitsverhalten und körperlicher Aktivität. In: Schüler J, Wegner M, Plessner H (Hrsg.). Sportpsychologie. Grundlagen und Anwendung. Springer Berlin Heidelberg, Berlin, Heidelberg.

Pfeifer K, 2009. Entwicklung evidenzgesichterter Konzepte für die Bewegungstherapie, osf.io/52r9z

Pfeifer K, Geidl W, 2017. Bewegungsempfehlungen für Erwachsene mit einer chronischen Erkrankung – Methodisches Vorgehen, Datenbasis und Begründung. Gesundheitswesen, 79 (S 01), S29-S35.

Pfeifer K, Geidl W, W., 2016. Bewegungsempfehlungen für Erwachsene mit einer chronischen Erkrankung - Methodisches Vorgehen, Datenbasis und wissenschaftliche Begründung. Gesundheitswesen, 78 (S01).

Pfeifer K, Geidl W, Zopf E, Baumann F, Schöne D, Hendrich S, Hofmann J, 2009. Entwicklung evidenzgesichterter Konzepte für die Bewegungstherapie, https://osf.io/52r9z/metadata/osf

Pfeifer K, Hänsel F, Heinz B, 2007. Rückengesundheit. Grundlagen und Module zur Planung von Kursen ; mit 8 Tabellen. Dt. Ärzte-Verl., Köln, x, 116 S.

Pfeifer K, Sudeck G, 2016. Körperliche Aktivität. In: Bengel J, Mittag O (Hrsg.). Psychologie in der medizinischen rehabilitation. Ein lehr- und praxishandbuch. Springer, Berlin, S. 215–228.

Pfeifer K, Sudeck G, 2020. Körperliche Aktivität. In: Bengel J, Mittag O (Hrsg.). Psychologie in der medizinischen Rehabilitation. Springer Berlin Heidelberg, Berlin, Heidelberg, S. 249–264.

Pfeifer K, Sudeck G, 2022. Sport- und Bewegungstherapie – Zielsetzungen, Betrachtungsperspektiven, Entwicklungsmöglichkeiten. In: Meyer, T., Bengel, J., Wirtz, A. (Hrsg.). Lehrbuch der Rehabilitationswissenschaften. Hogrefe-Verlag, Bern.

Pfeifer K, Sudeck G, Geidl W, Tallner A, 2013. Bewegungsförderung und Sport in der Neurologie – Kompetenzorientierung und Nachhaltigkeit. Neurol und Rehabilitation, 19 (1), 7–19.

Prochaska JO, DiClemente CC, 1982. Transtheoretical therapy: Toward a more integrative model of change. Psychotherapy: Theory, Research & Practice, 19 (3), 276–288.

Radlinger L, Bachmann W, Homburg J, Leuenberger U, Thaddey G, 1998. Rehabilitatives Krafttraining. Theoretische Grundlagen und praktische Anwendungen : 379 Abbildungen, 471 Einzeldarstellungen, 135 Tabellen. Georg Thieme Verlag, Stuttgart, New York, 366 S.

Rauen C (Hrsg.), 2021. Handbuch Coaching (4., vollständig überarbeitete und erweiterte Auflage). Hogrefe, Göttingen, 722 S.

Rethorn ZD, Bezner JR, Pettitt CD, 2022. From expert to coach: health coaching to support behavior change within physical therapist practice. Physiother Theory Pract, 38 (13), 2352–2367.

Rheinberg F, Engeser S, 2010. Chapter 18 Motive Training and Motivational Competence. In: Schultheiss O, Brunstein J (Hrsg.). Implicit Motives. Oxford University PressNew York, S. 510–548.

Rhodes RE, Bruijn G-J de, 2013. How big is the physical activity intention-behaviour gap? A meta-analysis using the action control framework. British Journal of Health Psychology, 18 (2), 296–309. Abgerufen am 15.02.2021.

Rhodes RE, Kates A, 2015. Can the affective response to exercise predict future motives and physical activity behavior? A systematic review of published evidence. Annals of behavioral medicine : a publication of the Society of Behavioral Medicine, 49 (5), 715–731.

Rhodes RE, McEwan D, Rebar AL, 2019. Theories of physical activity behaviour change: a history and synthesis of approaches. Psychol Sport Exerc, 42, 100–109.

Riebe D, Ehrman JK, Liguori G, Magal M (Hrsg.), 2018. ACSM's guidelines for exercise testing and prescription (Tenth edition). Wolters Kluwer, Philadelphia, Baltimore, New York, London, Buenos Aires, Hong Kong, Sydney, Tokyo, 472 S.

Rost R, Hollmann W, 1982. Belastungsuntersuchungen in der Praxis: Grundlagen, Technik und Interpretation ergometrischer Untersuchungsverfahren ; 16 Tabellen. Thieme, Stuttgart New York, 164 S.

Rütten A, Pfeifer K (Hrsg.), 2017. Nationale Empfehlungen für Bewegung und Bewegungsförderung. Bundeszentrale für gesundheitliche Aufklärung (BZgA), Köln, 88 S. https://www.bundesgesundheitsministerium.de/fileadmin/Dateien/5_Publikationen/Praevention/Broschueren/Bewegungsempfehlungen_BZgA-Fachheft_3.pdf.

Schmid J, Gut V, Schorno N, Sudeck G, Conzelmann A, 2024. Welcher Sport für wen? Motivationspsychologische Perspektiven zur Passung von Person und Sportaktivität. Hogrefe AG, Bern, 184 S.

Schmid J, Schorno N, Groux A, Giachino D, Zehetner J, Nett P, Nakas CT, Herzig D, Bally L, 2023. Fostering physical activity-related health competence after bariatric surgery with a multimodal exercise programme: A randomised controlled trial. Journal of behavioral medicine, 46 (5), 709–719.

Schön DA, 2017. The Reflective Practitioner. Routledge.

Schorno N, Sudeck G, Gut V, Conzelmann A, Schmid J, 2021. Choosing an activity that suits: development and validation of a questionnaire on motivational competence in exercise and sport. German Journal of Exercise and Sport Research, 51 (1), 71–78.

Schüle K, Huber G (Hrsg.), 2012. Grundlagen der Sport- und Bewegungstherapie. Prävention, ambulante und stationäre Rehabilitation (3., vollständig überarb. und erw. Aufl.). Deutscher Ärzte-Verlag, Köln, xvi, 522.

Seelig H, Fuchs R, 2006. Messung der sport- und bewegungsbezogenen Selbstkonkordanz. Zeitschrift für Sportpsychologie, 13 (4), 121–139.

Segar ML, Guérin E, Phillips E, Fortier M, 2016. From a Vital Sign to Vitality: Selling Exercise So Patients Want to Buy It, 15 (4).

Semrau J, Hentschke C, Buchmann J, Meng K, Vogel H, Faller H, Bork H, Pfeifer K, 2015. Long-term effects of interprofessional biopsychosocial rehabilitation for adults with chronic non-specific low back pain: a multicentre, quasi-experimental study. PloS one, 10 (3), e0118609.

Smith B, Kirby N, Skinner B, Wightman L, Lucas R, Foster C, 2019. Infographic. Physical activity for disabled adults. British journal of sports medicine, 53 (6), 335–336.

Smolenski UC, Buchmann J, Beyer L, Harke G, Seidel W, Pahnke J, Janda V, 2020. Janda Manuelle Muskelfunktionsdiagnostik: Theorie und Praxis (6., aktualisierte Auflage). Elsevier, München, 309 S.

Stevens A, Köke A, van der Weijden T, Beurskens A, 2018. The development of a patient-specific method for physiotherapy goal setting: a user-centered design. Disability and Rehabilitation, 40 (17), 2048–2055.

Strohacker K, Sudeck G, Keegan R, Ibrahim AH, Beaumont CT, 2024. Contextualising flexible nonlinear periodization as a person-adaptive behavioral model for exercise maintenance. Health psychology review, 18 (2), 285–298.

Sudeck G, 2006. Motivation und Volition in der Sport- und Bewegungstherapie. Konzeptualisierung und Evaluierung eines Interventionskonzepts zur Förderung von Sportaktivitäten im Alltag. Czwalina, Hamburg, 315 S.

Sudeck G, Geidl W, Abu-Omar K, Finger JD, Krauß I, Pfeifer K, 2021. Do adults with non-communicable diseases meet the German physical activity recommendations? German Journal of Exercise and Sport Research.

Sudeck G, Pfeifer K, 2016. Physical activity-related health competence as an integrative objective in exercise therapy and health sports – conception and validation of a short questionnaire. German Journal of Exercise and Sport Research, 46 (2), 74–87.

Sudeck G, Rosenstiel S, Carl J, Pfeifer K, 2020. Bewegungsbezogene Gesundheitskompetenz – Konzeption und Anwendung in Gesundheitsförderung, Prävention und Rehabilitation. In: Rathmann K, Dadaczynski K, Okan O, Messer M (Hrsg.). Gesundheitskompetenz. Springer Berlin Heidelberg, Berlin, Heidelberg, S. 1–12.

Sudeck G, Rosenstiel S, Carl J, Pfeifer K, 2023. Bewegungsbezogene Gesundheitskompetenz – Konzeption und Anwendung in Gesundheitsförderung, Prävention und Rehabilitation. In:

Rathmann K, Dadaczynski K, Okan O, Messer M (Hrsg.). Gesundheitskompetenz. Springer Berlin Heidelberg, Berlin, Heidelberg, S. 33–44.

Swann C, Rosenbaum S, Lawrence A, Vella SA, McEwan D, Ekkekakis P, 2021. Updating goal-setting theory in physical activity promotion: a critical conceptual review. Health psychology review, 15 (1), 34–50.

Teixeira PJ, Marques MM, Silva MN, Brunet J, Duda JL, Haerens L, La Guardia J, Lindwall M, Lonsdale C, Markland D, Michie S, Moller AC, Ntoumanis N, Patrick H, Reeve J, Ryan RM, Sebire SJ, Standage M, Vansteenkiste M, Weinstein N, Weman-Josefsson K, Williams GC, Hagger MS, 2020. A classification of motivation and behavior change techniques used in self-determination theory-based interventions in health contexts. Motivation Science.

Thiel A, John JM, Carl J, Thedinga HK, 2020. Weight Stigma Experiences and Physical (In)activity: A Biographical Analysis. Obesity facts, 13 (3), 386–402.

Tiemann M, 2006. Handlungswissen und Effektwissen. In: Bös K, Brehm W (Hrsg.). Handbuch Gesundheitssport. (2., vollständig neu bearbeitete Auflage). Hofmann, Schorndorf, S. 357–368.

U.S. Department of Health and Human Services, 2018. 2018 Physical activity guidelines advisory committee scientifc report. Abgerufen am 16.01.2022, https://health.gov/sites/default/files/2019-09/PAG_Advisory_Committee_Report.pdf

Vries N de, van Ravensberg D, Hobbelen J, van der Wees P, Olde R, 2015. The Coach2Move approach: development and acceptability of an individually tailored physical therapy strategy to increase activity levels in older adults with mobility problems. Journal of Geriatric Physical Therapy, 38 (4), 169–182.

Weiss H, Harrer ME, Dietz T, 2023. Das Achtsamkeitsbuch. Grundlagen, Übungen, Anwendungen (11., aktualisierte Auflage). Klett-Cotta, Stuttgart, 346 S.

Werle J, Woll A, Tittlbach S, 2006. Gesundheitsförderung: Körperliche Aktivität und Leistungsfähigkeit im Alter. Kohlhammer, Stuttgart.

WHO, 2001. International Classification of Functioning, Disability and Health: ICF. World Health Organization.

WHO, 2020. WHO guidelines on physical activity and sedentary behaviour. Abgerufen am 25.02.2022, https://www.who.int/publications/i/item/9789240015128

Willett M, Greig C, Fenton S, Rogers D, Duda J, Rushton A, 2021. Utilising the perspectives of patients with lower-limb osteoarthritis on prescribed physical activity to develop a theoretically informed physiotherapy intervention. BMC musculoskeletal disorders, 22 (1), 155, https://bmcmusculoskeletdisord.biomedcentral.com/articles/10.1186/s12891-021-04036-8

Williamson C, Baker G, Tomasone JR, Bauman A, Mutrie N, Niven A, Richards J, Oyeyemi A, Baxter B, Rigby B, Cullen B, Paddy B, Smith B, Foster C, Drummy C, Vandelanotte C, Oliver E, Dewi FST, McEwen F, Bain F, Faulkner G, McEwen H, Mills H, Brazier J, Nobles J, Hall J, Maclaren K, Milton K, Olscamp K, Campos LV, Bursle L, Murphy M, Cavill N, Johnston NJ, McCrorie P, Wibowo RA, Bassett-Gunter R, Jones R, Ruane S, Shilton T, Kelly P, 2021. The Physical Activity Messaging Framework (PAMF) and Checklist (PAMC): International consensus statement and user guide. The international journal of behavioral nutrition and physical activity, 18 (1), 164.

Wirtz M, Farin E, Bengel J, Jäckel WH, Hämmerer D, Gerdes N, 2005. IRES-24 Patientenfragebogen. Diagnostica, 51 (2), 75–87.

Wiskemann J, Scharhag-Rosenberger F, 2014. Nebenwirkungsorientierte Behandlungspfade für die bewegungstherapeutische Betreuung onkologischer Patienten. B&G Bewegungstherapie und Gesundheitssport, 30 (04), 146–150.

Wolf S, Zeibig J, Hautzinger M, Sudeck G, 2020a. Psychische Gesundheit durch Bewegung. Beltz, Weinheim, 200 S.

Wolf S, Zeibig J, Hautzinger M, Sudeck G, 2020b. Psychische Gesundheit durch Bewegung. ImPuls - ein sport- und bewegungstherapeutisches Programm für Menschen mit psychischen Erkrankungen. Mit E-Book inside und Arbeitsmaterial. Beltz, Weinheim, 200 S.
Worringen U, Geidl W, Reusch A, Prodinger B, Redzewsky L, Salzwedel A, Reudelsterz C, Herbold D, Zimmer J-M, Thomas A, Körner M, 2024. Ziele und Aufgaben der Arbeitsgruppe „Interprofessionelle Zusammenarbeit" in der DGRW. Die Rehabilitation, 63 (02), 131–137.
